# Are school‐based physical activity interventions effective and equitable? A meta‐analysis of cluster randomized controlled trials with accelerometer‐assessed activity

**DOI:** 10.1111/obr.12823

**Published:** 2019-01-09

**Authors:** Rebecca Love, Jean Adams, Esther M. F. van Sluijs

**Affiliations:** ^1^ Centre for Diet and Activity Research (CEDAR), MRC Epidemiology Unit University of Cambridge Cambridge UK

**Keywords:** children and adolescents, meta‐analysis, physical activity, systematic review

## Abstract

The prevalence of childhood obesity is increasing at epidemic rates globally, with widening inequalities between advantaged and disadvantaged groups. Despite the promise of schools as a universal context to access and influence all children, the potential of school‐based interventions to positively impact children's physical activity behaviour, and obesity risk, remains uncertain. We searched six electronic databases to February 2017 for cluster randomized trials of school‐based physical activity interventions. Following data extraction, authors were sent re‐analysis requests. For each trial, a mean change score from baseline to follow‐up was calculated for daily minutes of accelerometer‐assessed moderate‐to‐vigorous physical activity (MVPA), for the main effect, by gender, and by socio‐economic position (SEP). Twenty‐five trials met the inclusion criteria; 17 trials provided relevant data for inclusion in the meta‐analyses. The pooled main effect for daily minutes of MVPA was nonexistent and nonsignificant. There was no evidence of differential effectiveness by gender or SEP. This review provides the strongest evidence to date that current school‐based efforts do not positively impact young people's physical activity across the full day, with no difference in effect across gender and SEP. Further assessment and maximization of implementation fidelity is required before it can be concluded that these interventions have no contribution to make.

## 1 INTRODUCTION

The worldwide prevalence of childhood obesity has increased tenfold over the past four decades.^1^ Obesity in childhood increases the risk of noncommunicable diseases in adulthood, which are estimated to cause 71% of the world's deaths.^2^ The lifetime health care and productivity costs of childhood obesity have been estimated at €149,206 per child.^3^ Physical inactivity is a key contributor to childhood obesity,^4^ and international guidelines recommend that young people aged 5–18 years accumulate “at least 60 minutes of moderate‐to‐vigorous physical activity (MVPA) daily”.^5^ However, globally 81% of adolescents do not meet these guidelines.^6^ Furthermore, physical inactivity is socially patterned, contributing to inequalities in associated health outcomes.^7^ In particular, children who are socioeconomically disadvantaged and girls are more likely to be physically inactive than children who are more advantaged and boys.^8^, ^9^ Promoting health equity by reducing inequalities in health behaviours is increasingly a priority for national and supranational bodies.^10^

Governments worldwide are prioritizing obesity prevention and health equity promotion through, amongst other things, increasing physical activity in young people. As schools offer a context to reach the majority of young people irrespective of background characteristics, they provide an obvious intervention setting. However, evidence for the effectiveness of school‐based physical activity interventions is mixed,^11^ with positive effects proving challenging to maintain over the long term.^12^ Furthermore, it is unclear whether population subgroups benefit equally from current efforts. There is theoretical and empirical evidence that public health interventions can exacerbate existing inequalities via differential effects between population subgroups.^13^ Inequitable effects have been demonstrated in some school‐based physical activity interventions,^14^ but there is an overall scarcity of evidence on this possibility.^15^ Even a null effect overall may mask differential effects between population subgroups.

The majority of early evidence on school‐based physical activity interventions showed positive effects, but used self‐report measures,^16^, ^17^ which have limited validity and differential bias across population subgroups.^18^ Whilst more recent reviews are restricted to objective measures, they commonly combine data from a variety of tools (eg, accelerometers and pedometers),^19^ measurement periods (eg, recess only and whole day) and outcomes (eg, MVPA and average activity intensity).^20^, ^21^ The potential impact of this is exemplified by one trial in which the effect estimate on accelerometer‐assessed activity during school (when children were directly exposed to the intervention) was more than four times the effect across the full day (*z* Scores: 0.92 vs. 0.21, respectively).^22^ Given that most school‐based interventions are designed to affect total activity across the day, and that total MVPA is most strongly associated with different health benefits,^23^ the most rigorous evaluation of the overall and equitable impact of school‐based physical activity interventions requires a focus on whole day MVPA.

Our recent scoping review of physical activity interventions in young people revealed an overall scarcity of published evidence on equity effects.^15^ However, it identified that substantial relevant unpublished data were available—particularly in terms of gender and socioeconomic position (SEP) in relation to school‐based interventions. Here, we therefore aimed to systematically review and meta‐analyse data on the overall effectiveness of school‐based physical activity interventions on accelerometer‐assessed daily minutes of MVPA, and investigate if this effect varies by gender or SEP.

## 2 METHODS

This systematic review and meta‐analysis is reported according to the PRISMA guidelines. The protocol was registered with PROSPERO (ref CRD42017062565) and is included as Supplementary information (S1). There were no substantive changes to protocol.

### 2.1 Search strategy and selection criteria

The literature search was conducted in six electronic databases (ERIC, EMBASE, OVID MEDLINE, PsycINFO, Scopus, and SPORTDiscus), originally in May 2016 (scoping review), and updated for the current review in February 2017. The search aimed to identify controlled trials of physical activity promotion in young people that used objective measures of physical activity. The search strategies were prepiloted with no restrictions by publication year, geographic location, or other socio‐demographic indicators. The search strategy as conducted in Medline is included as S2. Additionally, published systematic reviews from the field were searched to check for any missing studies.^16^, ^17^, ^20^, ^24^

In order to focus on a homogeneous pool of trials and enable in‐depth exploration of equity effects, and on the basis of our assessment of data availability, the inclusion criteria from the scoping review were made more restrictive for the current review. We limited inclusion to interventions conducted primarily in schools (84/113 trials included in scoping review), and to cluster‐randomized (at the school or classroom level) controlled trials, which used accelerometers to assess activity across the whole day. The full inclusion criteria are outlined in Table 1.

**Table 1:** Study inclusion and exclusion criteria for systematic review and meta‐analysis of school‐based physical activity interventions

	Included	Excluded
Population	•school‐aged children and adolescents, 6‐18 y of age at baseline	•preschool populations of children (5 y of age and younger)
		•children selected on the basis of having a specific disease or special needs (including obesity at a 95 percentile cut off point)
Intervention	•school‐based single or multicomponent interventions of at least 4 wk duration aimed at increasing physical activity	•interventions with a duration less than 4 wk
		•interventions implemented solely within community and home environments
Study design	•cluster‐randomized (at the classroom or school level) controlled trials	•interventions randomized at the individual level
		•interventions described as pilot or feasibility studies
Comparator	•trials with a minimal intervention or no intervention comparison group	•trials comparing two active intervention arms
Outcomes	•Acclerometery‐assessed physical activity across the whole day at baseline and follow‐up, in the same participants	•subjectively measured physical activity outcomes (eg, self‐report questionnaires)
		•nonaccelerometer forms of objective physical activity outcomes (eg, pedometers and heart rate)
		•physical activity outcome data not collected in the same children at baseline and follow up
		•physical activity outcomes examining only part of the day activity (eg, recess or breaktime)
Publication type	•peer reviewed journal article	•conference abstract, study protocol, report, dissertation, and book

Following deduplication, title, and abstract screening removed papers clearly outside of the scoping review inclusion criteria. The selection was performed by one reviewer, with a 15% random sample double checked by a second reviewer (coder agreement rate was 98%). Full text screening was performed independently by the same two reviewers. All discrepancies were resolved through discussion.

Intervention characteristics were extracted from included trials using a prepiloted data extraction form. Data extraction was performed in duplicate by two reviewers, and included baseline descriptives, study name and design, intervention and outcome characteristics, reported intervention main effect, and effects across gender and SEP (see S3 for a complete list of items).

Quality assessment was performed independently by two reviewers using the Cochrane Collaboration's risk of bias tool. Studies were assessed across each of the five domains of bias (selection, performance, attrition, detection, and reporting) and classified as presenting a low, high, or unclear risk of bias. In the case of disagreement on data extraction or quality assessment, consensus was determined by consulting the third reviewer.

None of the included trials reported sufficient relevant data for the planned analyses, and thus all authors were contacted to obtain further information. Corresponding authors of the main trial publications were contacted in May 2017 by email. Data request forms were precompleted as far as possible from published papers and authors requested to further complete these. Requested data included sample size (N), mean, and standard deviation (SD) of daily minutes of MVPA at baseline and all follow‐ups for both intervention and control groups, for the main intervention effect, and stratified both by gender, and SEP. If possible, we requested authors categorize SEP into three groups (low, middle, and high, as defined by the author). Where this was not possible, two groups representing low and high SEP were accepted. As there are many possible measures of SEP, we provided authors with a preference hierarchy: (1) parental education (maternal preferable to paternal), (2) area‐based markers of deprivation (eg, Index of Multiple Deprivation or postal code‐based indices), and (3) household income equivalized for household composition. This hierarchy was developed based on research evaluating the importance of measures of socioeconomic status in child and adolescent populations.^25^, ^26^ The decision was also pragmatically based on data availability as assessed in data extraction. The full request details and data extraction form is included as S4.

### 2.2 Data analysis

To assess overall and differential intervention effects on MVPA, mean change scores from baseline to follow‐up were calculated for intervention and control groups. For each analysis the post‐intervention follow‐up time closest to intervention end point was utilized. Intervention effects were calculated by dividing the between group difference of mean change in minutes of MVPA from baseline by the pooled SD of change in MVPA for the intervention and control group, assuming a correlation of *r* = 0.5 between baseline and follow‐up (see S5 for full formula).^27^

Effect sizes were calculated using Hedges' g and utilized in meta‐analyses. Random effects meta‐analyses were chosen as heterogeneity was expected given differences in study populations and interventions. Differences in effect by gender and SEP were tested statistically by performing meta‐regressions on the stratifying variable in a meta‐analysis model pooling the individual subgroups for that characteristic.

Statistical heterogeneity was assessed visually using forest plots and quantified using the χ^2^ and I^2^ statistics. By convention, I^2^ values of 25% were consider low, 50% moderate, and 75% high. The potential for publication bias was assessed visually using funnel plots and Egger's test for funnel plot asymmetry. Since the use of random effect models may overestimate treatment effects, fixed effect models (which produce more conservative estimates) were also conducted and compared as a sensitivity analysis (see results presented in S9).

Preplanned subgroup meta‐analyses and a series of meta‐regression were performed to examine if selected intervention characteristics explained heterogeneity in effect sizes (if I^2^ ≥ 50%). Three continuous variables (intervention duration, sample size, and mean participant age) were tested in meta‐regressions through multivariable random effects models. To consider between‐trial variance, a method of moments, random effects meta‐analysis was utilized. Subgroup analyses were then run to investigate if heterogeneity could be explained by categorical characteristics of interest (intervention components, behavioural approach, intervention setting, and risk of bias summary score).

## 3 RESULTS

**Figure 1:**
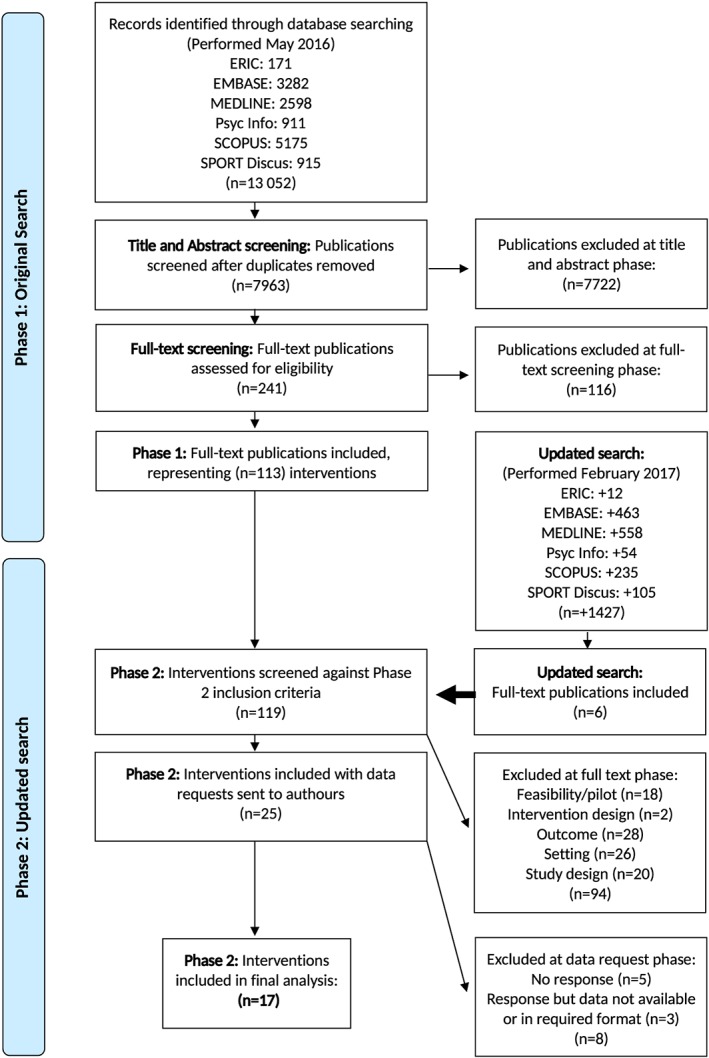
PRISMA flow chart of study selection for meta‐analysis of school‐based physical activity interventions [Colour figure can be viewed at wileyonlinelibrary.com]

Figure 1 shows the PRIMSA flow chart for the entire review process. Twenty‐five trials met the inclusion criteria for this review. The reasons for exclusion at the full text phase (n = 119) are outlined in S6. Eight trials were excluded from meta‐analyses following data requests (n = 25) because of: no response (n = 5), data being unavailable (n = 1), or data not provided in the required format (n = 2) (See S7). Characteristics of the final 17 trials included in the meta‐analyses are summarized in Table 2 and S8. 22, 28, 29, 30, 31, 32, 33, 34, 35, 36, 37, 38, 39, 40, 41, 42, 43

**Table 2:** Characteristics of trials included in meta‐analysis of school‐based physical activity interventions (n = 17)

Country of implementation (no [%])	
Australia	4 (23.5%)
Northern Europe	5 (29.5)
Western Europe	5 (29.4%)
Central Europe	1 (5.9%)
North America	1 (5.9%)
South America	1 (5.9%)
Level of randomization	
School	13 (76.0%)
Classroom	4 (24.0%)
Intervention components^a^	
Educational	14 (82.3%)
Social environment	17 (100.0%)
Physical environment	3 (17.6%)
Intervention setting	
School plus afterschool/community components	13 (76.5%)
School only	4 (23.5%)
Behavioural approach	
Targeting PA only	10 (58.8%)
Targeting PA alongside other health behaviours	7 (41.2%)
Mean baseline sample size	464 (median: 436; interquartile range [IQR]: 178‐700)
Mean number of schools per trial	20 (median: 14; IQR: 12‐18)
Mean intervention duration	9 months (median: 6; IQR: 5‐12)
Mean age	10·6 years (median: 11·2; IQR: 9·5‐2·0)

*Note*. PA: physical activity.

a Categories are not mutually exclusive.

The mean baseline sample size of included trials was 464 participants (median: 436; inter‐quartile range (IQR): 178‐700). The duration of interventions ranged from 1.5 to 24 months, with a median of 6 months (IQR: 5‐12). The majority of included trials were conducted in Europe (65%) followed by Australasia (23.5%), North America (5.9%), and South America (5.9%). Overall, 53% of trials presented a high‐risk of bias summary score, 18% low and 29% unclear.

### 3.1 Main intervention, gender, and SEP intervention effects

**Figure 2:**
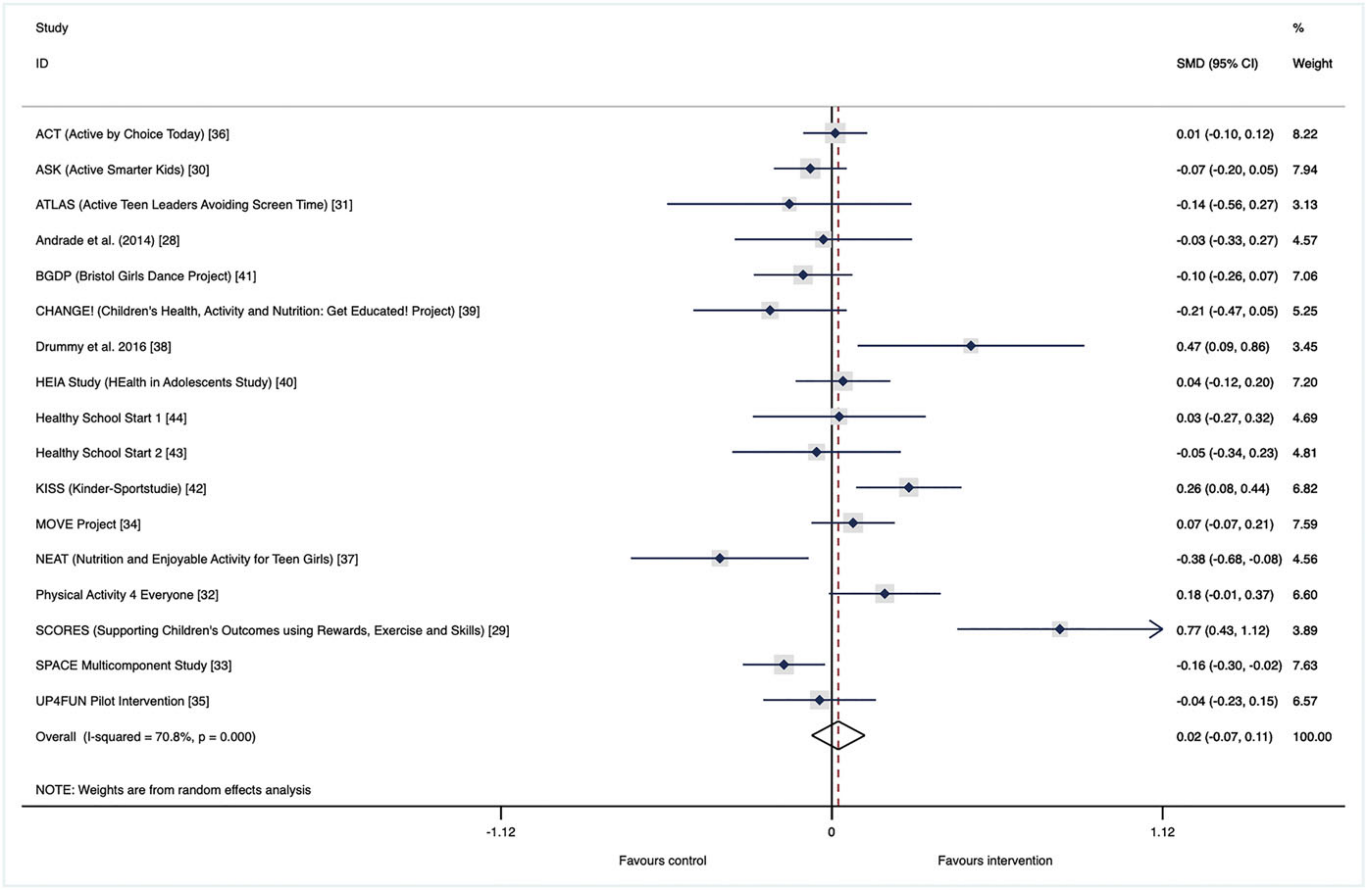
Main effect. Forest plot of standardized mean difference of change in physical activity between intervention and control groups of school‐based physical activity interventions (study name [reference]) [Colour figure can be viewed at wileyonlinelibrary.com]

The main effect meta‐analysis showed a nonexistent (SMD: 0.02) and nonsignificant (95% CI, −0.07‐0.11) pooled effect of interventions on daily minutes of MVPA (Figure 2).

**Figure 3:**
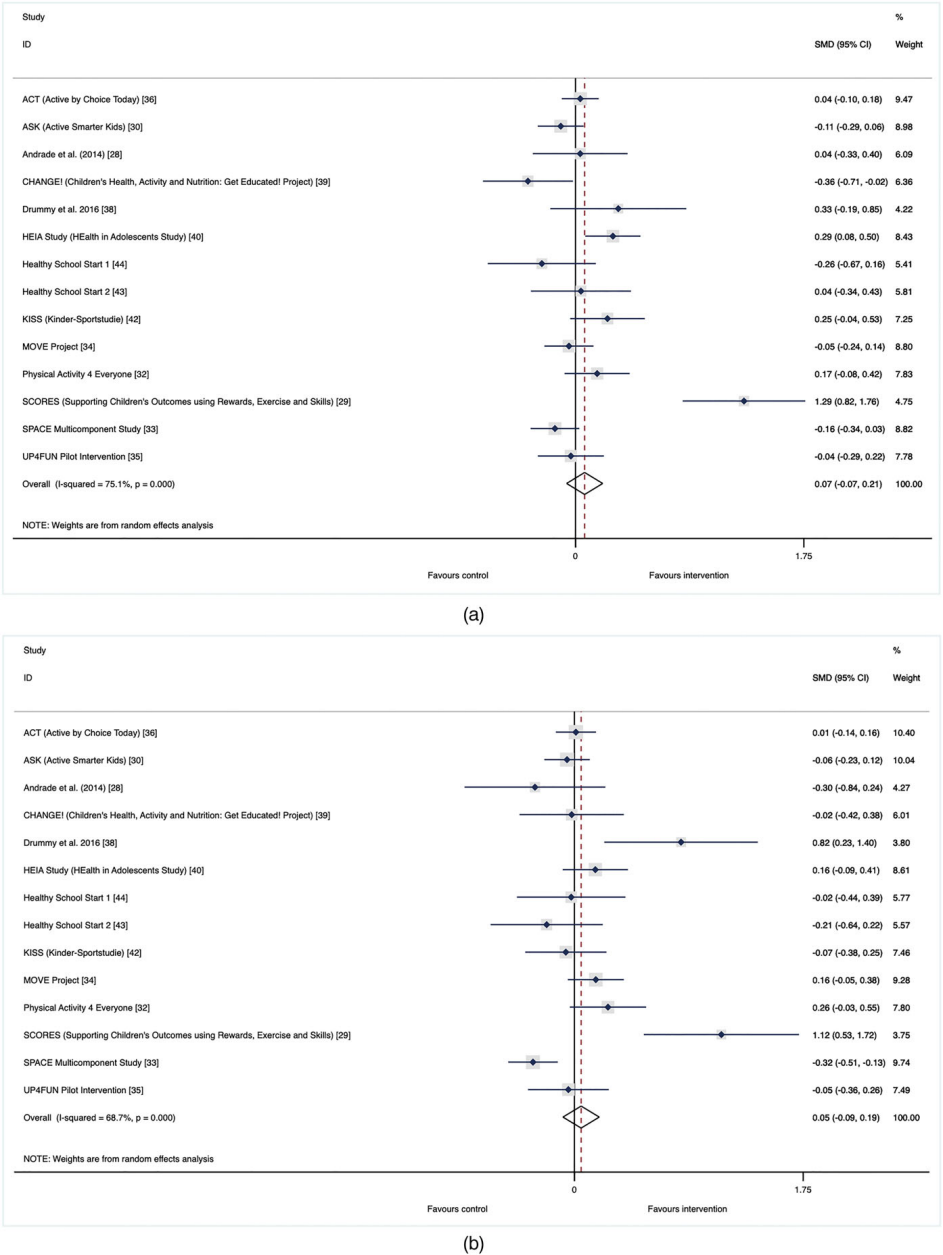
(a,b). Gender effect. Forest plots of standardized mean difference of change in physical activity for (a) girls and (b) boys between intervention and control groups of school‐based physical activity interventions (study name [reference]) [Colour figure can be viewed at wileyonlinelibrary.com]

Figure 3 outlines the intervention effects by gender. The girls' meta‐analysis indicated a trivial (SMD: 0.07), but nonsignificant effect (95% CI, −0.07‐0.21). Similar findings were seen for boys (SMD: 0.05; 95% CI, −0.09‐0.19). There was also no evidence of a statistically significant difference in intervention effect between girls and boys (*P*‐value: 0.97).

**Figure 4:**
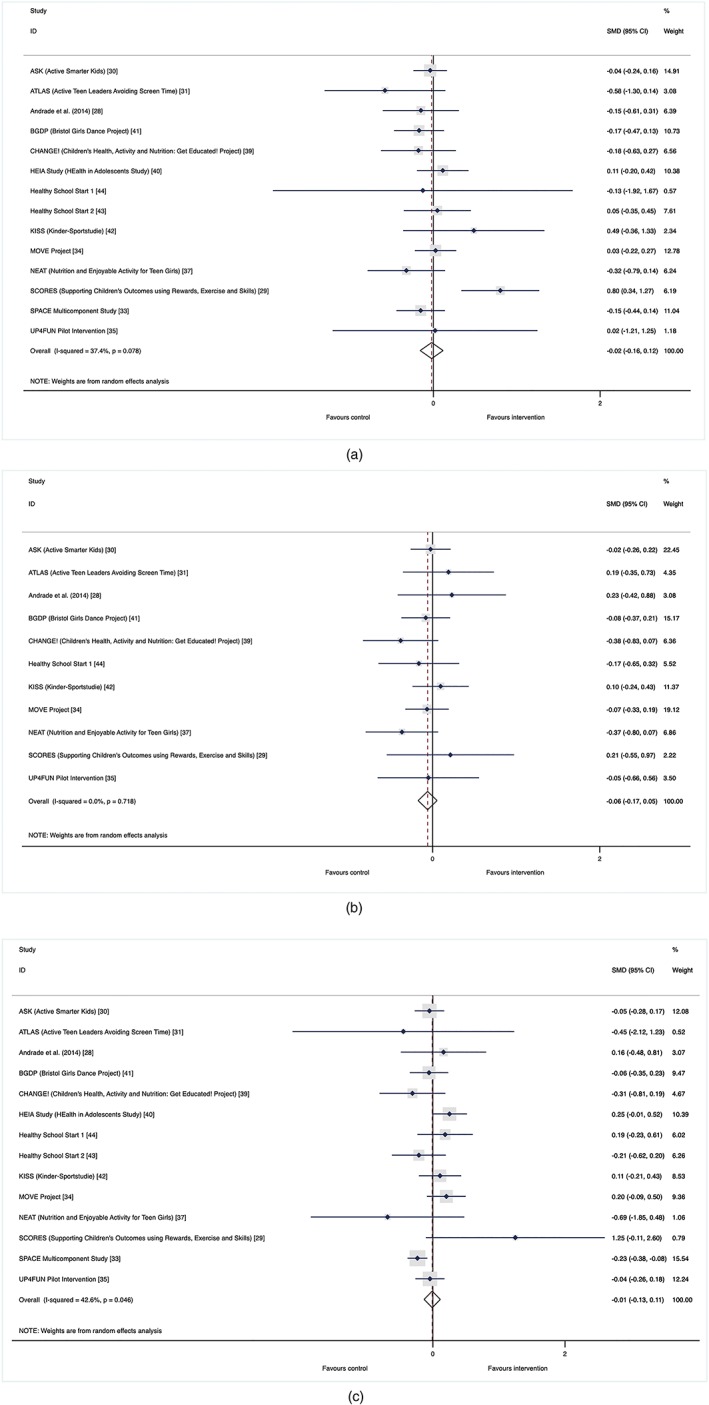
(a‐c). Socioeconomic position (SEP) effect. Forest plots of standardized mean difference of change in physical activity by tertiles of SEP for (a) low SEP, (b) middle SEP, and (c) high SEP between intervention and control groups of school‐based physical activity interventions (study name [reference]) [Colour figure can be viewed at wileyonlinelibrary.com]

Similarly, there was no evidence of differential intervention effect by SEP. Figure 4 outlines the effect on children from low SEP (SMD: −0.02, 95% CI, −0.16‐0.12), middle SEP (SMD: −0.06, 95% CI, −0.17−0.05) and high SEP (SMD: −0.01, 95% CI, −0.13‐0.11) backgrounds. There was no evidence of a statistical difference in intervention effectiveness by SEP (*P*‐value: 0.68).

### 3.2 Publication bias

Eggers test for asymmetry of the funnel plot, was not significant (Coef: −0.08, *P*‐value: 0.49), indicating no evidence of publication bias (See S9).

### 3.3 Exploration of heterogeneity: Meta‐regressions and subgroup analyses

Meta‐regressions revealed no evidence of heterogeneity by sample size (*P*‐value: 0.57), intervention duration (*P*‐value: 0.98), age (*P*‐value: 0.12) (See S9). There was a nonsignificant trend towards a decrease in SMD with increasing mean participant age.

Subgroup meta‐analyses by intervention characteristics of interest (behavioural approach, intervention setting, and risk of bias summary score) revealed no significant differences in effect estimates (See S9). There was insufficient heterogeneity in intervention components (social environment, physical environment, and educational components) to enable subgroup analyses.

## 4 DISCUSSION

This systematic review and meta‐analyses provide the strongest collated evidence to date on the effectiveness school‐based physical activity interventions. We found that when restricted to cluster‐randomized controlled evidence utilizing accelerometer‐measured outcomes, school‐based interventions in children and adolescents are not effective in increasing minutes spent in MVPA across the full day, and this did not differ by gender or SEP.

To our knowledge, this is the first meta‐analysis in young people's physical activity promotion to pool accelerometer data with comparable outcome metrics. To rigorously answer our research questions, we collated mean daily minutes of MVPA measured by accelerometer. This decision was made in consideration of: a need for objective measurements that are equally valid across population subgroups,^18^ the importance of full day activity change for health benefit,^23^ and evidence of differential health benefits related to different physical activity intensities.^44^ Whilst accelerometers have been shown to provide valid and reliable estimates of physical activity in children, they have inherent limitations including an inability to classify behaviour, detect certain activities (eg, cycling and swimming), upper body movements, or changes in terrain.^45^ Successful author re‐analysis requests enabled, for the first time, the pooling of intervention accelerometer data with comparable outcome metrics. Moreover, standardized and complete outcome data (N, mean, and SD), permitted the utilization of mean change effect estimates, an approach that strengths the robustness of the findings by accounting for group baseline differences.^46^ However, the analyses included only a subset of relevant available data (n = 8 were excluded because of inadequate or unavailable data). Calculation of Rosenthal's failsafe number, representing the number of studies that would be required to refute the main effect meta‐analytic conclusion, indicates low potential for biased conclusions.^47^ We estimate that at least 20 further trials, all with significant and positive intervention effects, would be needed to alter the main findings (see S10). Moreover, no evidence of publication bias was observed, even in the subset of studies included in the analyses. Lastly, whilst it is concerning that 53% of included trials had an overall high risk of bias score, a subgroup meta‐analysis by risk of bias was not significant. High risk of bias scores were primarily driven by attrition and lack of clarity regarding how missing data were handled within the analyses (S11).

The major strengths of this review compared with previous work are the pooling of comparable accelerometer‐based outcome measures of full‐day MVPA and assessment of equity effects. In contrast, previous reviews used either self‐reported outcomes,^16^, ^17^ or pooled effects of incomparable outcomes derived from objective tools.^20^, ^21^ We restricted inclusion to objective measurements given evidence of poor validity and reliability of self‐report and observational methods.^18^ Additionally, given growing evidence of differences in activity intensities and patterning between subgroups of children,^48^ we restricted inclusion to trials for which we could obtain accelerometer assessed minutes of MVPA across the full day. Our scoping review,^15^ identified that asking authors to conduct re‐analysis was the only way to obtain relevant data on equity effects by gender and SEP. Thus, in addition to providing a pool of comparable data, these author requests allowed us to exploit the potential of much data that had been collected, but not previously reported on. Restricting inclusion to a homogeneous group of school‐based trials limits the generalizability of our findings to school‐based efforts to promote physical activity. However, this represents the majority of the available evidence and maximized the reliability and robustness of our conclusions.

Whilst a lack of an overall effect could mask opposing effects in different population subgroups, we found no evidence of an effect in any gender or SEP subgroups. This suggests either that the intervention components are not effective or that they are not reaching target populations, rather than they are effective in some groups but not others. Substantial effort is commonly devoted to intervention theory and development, as demonstrated by the included KISS and CHANGE! trials.^49^, ^50^ We suggest that similar attention is now required to understand the intervention implementation process of these complex interventions and how this can be optimized in different contexts. The complex and multicomponent nature of most school‐based physical activity interventions may make them particularly vulnerable to poor implementation fidelity.^51^ Prior evaluations have demonstrated considerable differences in intervention intensities between classes and schools.^52^ Process evaluations are critical to understanding implementation success and the contextual factors that influence how an intervention works. However, on the basis of the pool of studies included in this review, process evaluations are rare: only 24% (n = 4) of included trials conducted a process evaluation.^53^, ^54^, ^55^, ^56^ Three of these process evaluations assessed the issue of intervention fidelity, each concluding wide variance in implementation of the program across schools and settings. Beyond determining if the intervention “worked”, outcome evaluations do little to inform future theory development, or context‐specific policy and practice. Robust evaluations of interventions known to be delivered with maximum possible implementation fidelity are required to confirm that school‐based interventions are not effective in changing physical activity. Until then, we recommend that school‐based activity promotion interventions are only implemented in research contexts and that investigators make substantial efforts to maximize, measure, and understand the impact of implementation fidelity across the intervention process.

Despite the promise of schools as a universal context to influence health behaviours, our review and emerging trial evidence,^57^ suggest that current efforts are not having an impact. It is unlikely that we will make substantial changes to population levels of, and inequities in, physical inactivity and obesity in children by focusing our collective efforts on only one setting, such as schools, when the wider environments are insufficiently supportive for behaviour change.^58^, ^59^ This is exemplified by some trials reporting positive effects during school hours, which are attenuated when assessing activity across the whole day as analysed here.^22^ Multidimensional intervention strategies across settings are likely required to achieve sustained effects across the whole day. In evaluations, the contribution of different components within such strategies needs to be carefully considered and assessed to maximize cost‐effectiveness.

This review focused on a subset of the literature on physical activity promotion in young people: school‐based interventions. We also restricted our assessment of equity effects to gender and SEP. Our scoping review revealed sufficient RCTs in school settings utilizing objective physical activity measures across the full day, however limited data on equity characteristics beyond gender and SEP.^15^ There is, thus, a need for further primary research in different intervention contexts using high‐quality outcome measures, and reporting outcomes both overall and across a range of different equity subgroups. This may require coordinated effort towards fewer, high‐quality studies, powered to detect subgroup differences. Given theoretical and empirical evidence that interventions can be differentially effective across population subgroups,^13^ it is critical that relevant equity characteristics are assessed. Whilst it may not be possible to power all studies to address equity questions, consistently collecting these data will enable future meta‐analyses like ours. It may also be timely to consider the standardization of outcome reporting in physical activity trials. In 35% of trials included in this review, published conclusions of positive effects were not confirmed in our re‐analysis using the a‐priori established outcome measure of accelerometer‐derived minutes of MVPA across the whole day.^34^, ^36^, ^39^, ^40^, ^60^, ^61^ All interventions included in this review were hypothesized to change activity across the whole day and whilst individual trials may have had different primary outcomes for good reason, it is important not to lose sight of the overarching aim of physical activity promotion—to improve health outcomes. This requires a focus on full day behaviour, and an increased understanding of effectiveness across times and settings.^62^ We further encourage, at a minimum that authors are accommodating to re‐analysis requests. Working towards more broad availability of data would further facilitate transparent evidence synthesis.

## 5 CONCLUSION

This systematic review and meta‐analysis demonstrate that school‐based physical activity interventions have not been effective at increasing children's accelerometer‐measured daily time spent in MVPA. This null effect is equitable across gender and SEP. These null results may be due to well‐designed interventions not reaching the target populations as intended, or effects not maintained across the day. Further assessment and maximization of implementation fidelity is required before it can be concluded that school‐based activity promotion interventions have no contribution to make to reducing physical inactivity and obesity in children. We recommend that for now, further school‐based activity promotion interventions should continue to be conducted in a research context.

## AUTHORS' CONTRIBUTIONS

R.L, E.v.S., and J.A designed the study. R.L. performed the literature searches. R.L. and E.v.S. conducted the title, abstract, and full text screening, R.L. and J.A. conducted data extraction and risk of bias assessments, R.L. drafted the manuscript. All authors contributed to the interpretation of the results and critically reviewed the manuscript. All authors read and approved the final manuscript. R.L. is the guarantor and responsible for the overall content.

## CONFLICT OF INTEREST

Dr Adams reports grants from the Medical Research Council during the conduct of the study. Dr van Sluijs reports grants from the MRC, ESCR, and Wellcome Trust outside of the submitted work. R. Love was nothing to disclose.

## PROSPERO REGISTRATION

PROSPERO 2017: CRD4201706256

## Supporting information

Data S1. SI: PROSPERO Registration
S2: Medline Search Strategy
S3: Data extracted
S4: Template of data request form utilized
S5: Formula for imputing the standard deviation of the change
S6: Trials excluded in full text screening
S7: Email request responses
S8: Characteristics of included studies
S9: All figures 9.1 – 9.51
S10: Failsafe ratio of included trials
S11: Risk of Bias assessment of included studies
S12: PRISMA Checklist
