# Supplementary material for: Are school‐based physical activity interventions effective and equitable? A meta‐analysis of cluster randomized controlled trials with accelerometer‐assessed activity
Source: Obes Rev. 2019 Jan 9;20(6):859–70. doi: 10.1111/obr.12823 (PMC6563481; doi:10.1111/obr.12823)
Supplement: Supplementary file 1 — Data S1. SI: PROSPERO Registration S2: Medline Search Strategy S3: Data extracted S4: Template of data request form utilized S5: Formula for imputing the standard deviation of the change S6: Trials excluded in full text screening S7: Email request responses S8: Characteristics of included studies S9: All figures 9.1 – 9.51 S10: Failsafe ratio of included trials S11: Risk of Bias assessment of included studies S12: PRISMA Checklist [file OBR-20-859-s001.pdf]

Supplementary Files

Contents

SI: PROSPERO Registration 2
S2: Medline Search Strategy 6
S3: Data extracted 8
S4: Template of data request form utilized 10
S5: Formula for imputing the standard deviation of the change 13
S6: Trials excluded in full text screening 14
S7: Email request responses 25
S8: Characteristics of included studies 26
S9: All figures 9.1 – 9.51 32
S10: Failsafe ratio of included trials 60
S11: Risk of Bias assessment of included studies 60
S12: PRISMA Checklist 63

SI: PROSPERO Registration

PROSPERO
International prospective register of systematic reviews

National Institute for Health Research

Equity effects of children's school-based physical activity interventions across gender and socioeconomic position

Rebecca Love, Esther van Sluijs, Jean Adams

Citation

Rebecca Love, Esther van Sluijs, Jean Adams. Equity effects of children's school-based physical activity interventions across gender and socioeconomic position. PROSPERO 2017 CRD42017062565 Available from:
http://www.crd.york.ac.uk/PROSPERO/display_record.php?ID=CRD42017062565

Review question

Are children's school-based physical activity interventions differentially effective across girls and boys?
Are school-based physical activity interventions are differentially effective across socioeconomic position (SEP) subgroups of children?

Searches

This analysis is the second stage of a scoping review (PROSPERO 2016: CRD42016034020). The original searches will be updated by searching the following databases:
- ERIC
- EMBASE
- Scopus
- PsycINFO
- OVID MEDLINE
- SPORTDiscus

Restrictions:
The following restrictions will be applied to the updated search:
- They must be English language journals;
- Population: must be children and adolescents (6-18 years of age) in school;
- They must be studies which have recruited samples from the general population (children and adolescents selected on the basis of having a specific disease, special needs or defined as obese will be excluded);
- Intervention: studies must include single or multicomponent interventions aimed at increasing children's and adolescents' levels of physical activity, primarily through the school environment;
- Study design: must be cluster randomised controlled trials with a control or minimal intervention control group;
- Outcomes: must be accelerometer-assessed physical activities in the same participants at baseline and follow-up.

Types of study to be included

Inclusion criteria:Only cluster randomised controlled trials (at the classroom or school level) will be included. Exclusion criteria:- Individually randomized controlled trials.- Non-randomized controlled trials. - Trials comparing two active intervention arms.- Interventions described as pilot or feasibility studies.

Condition or domain being studied

Physical activity during childhood and adolescence plays a critical role in promoting health and well-being and reducing future disease risk. Yet, most children and adolescents are not active enough to benefit their health.

Participants/population

Inclusion criteria:
- Children and adolescents (6-18 years of age) in school.

- Study populations recruited from the general population.
Exclusion criteria:
- Pre-school populations of children.
- Children and adolescents selected on the basis of having a specific disease or special needs.
- Studies in which participants are defined as obese, and the sample is restricted to an obese population.

Intervention(s), exposure(s)

Inclusion criteria:
- Single- or multi-component school-based interventions aimed at increasing physical activity in children.
- The intervention must of been delivered centrally through the school setting.
Exclusion criteria:
- Interventions with a duration of less than 4 weeks.
- Interventions implemented centrally in the home, the community or in a primary care setting.

Comparator(s)/control

Inclusion criteria:
- Interventions can have been compared with a control intervention (standard or usual care), or with a minimal intervention control group.
Exclusion criteria:
- Control conditions must not have included any physical activity components beyond the standardized/regular physical education curriculum.

Primary outcome(s)

Trials must have measured physical activity objectively, using accelerometers, at baseline and follow-up in the same participants.
Measurements of full day activity levels (both within and outside of school) must have been attempted.

Secondary outcome(s)

None.

Data extraction (selection and coding)

The following information on the differential effects will be extracted from all articles within the final pool of studies. Data extraction will be performed by RL with 100% being double checked for consistency by EvS. Individual data extraction sheets will be prepared for each included study.
The data to be extracted will include:
- Baseline characteristics (mean participant age, gender and SEP % of baseline sample, school setting); study design (cluster level); intervention characteristics (design, components, setting, behavioural approach, theory basis, duration intensity); accelerometer (brand and type, wear location, cut points, valid days, wear time); outcome measure, follow-up times, main intervention effect (N, mean, SD); gender differential effect (N, mean, SD for boys and girls); SEP differential effect (indicator used, how it was defined).
The authors of trials which do not report on outlined variables will be contacted by email, and re-analysis requested. After three weeks, authors who have not responded will be sent a reminder email, and a cut off point will be set two weeks after this reminder (i.e. five weeks after the initial request).

Risk of bias (quality) assessment

Two reviewers will independently quality assess all included studies using the Cochrane Collaboration's risk of bias tool. Studies will be assessed for the five domains of bias (selection, performance, attrition, detection and reporting) and classified within each as presenting a low, high or unclear risk of bias. Following the assessment of the distribution of risk of bias scores, a subset of low quality/high risk of bias trials will be excluded.

Strategy for data synthesis

Depending on the availability of homogenous data and trials, we plan to run meta-analyses to look at the differences in intervention effects by gender (girls compared to boys) and by socioeconomic position (across

the three tertiles).

Analysis of subgroups or subsets

If the necessary data is available, subgroup analyses will be conducted to investigate whether different types of interventions are driving different intervention effects by gender and socioeconomic status.

Contact details for further information

Rebecca Love
rel54@medschl.cam.ac.uk

Organisational affiliation of the review

MRC Epidemiology Unit & Centre for Diet and Activity Research (CEDAR)

Review team members and their organisational affiliations

Ms Rebecca Love. Centre for Diet and Activity Research (CEDAR), MRC Epidemiology Unit
Dr Esther van Sluijs. Centre for Diet and Activity Research (CEDAR), MRC Epidemiology Unit
Dr Jean Adams. Centre for Diet and Activity Research (CEDAR), MRC Epidemiology Unit

Anticipated or actual start date

01 February 2017

Anticipated completion date

01 November 2017

Funding sources/sponsors

Centre for Diet and Activity Research (CEDAR), a UKCRC Public Health Research Centre of Excellence [RES-590-28-0002]. Funding from the British Heart Foundation, Cancer Research UK, Economic and Social Research Council, Medical Research Council, the National Institute for Health Research, and the Wellcome Trust, under the auspices of the UK Clinical Research Collaboration, is gratefully acknowledged. This work is additionally supported by the Medical Research Council [MC_UU_12015/7].

Conflicts of interest

None known

Language

English

Country

England

Stage of review

Review_Ongoing

Subject index terms status

Subject indexing assigned by CRD

Subject index terms

Cardiorespiratory Fitness; Child; Exercise; Gender Identity; Health Behavior; Health Promotion; Health Status Disparities; Humans; Physical Fitness; School Health Services; Schools; Socioeconomic Factors

Date of registration in PROSPERO

18 May 2017

Date of publication of this version

18 May 2017

Details of any existing review of the same topic by the same authors

This analysis is the second stage of a scoping review (PROSPERO 2016: CRD42016034020).

Stage of review at time of this submission

Stage	Started	Completed
Preliminary searches	Yes	Yes
Piloting of the study selection process	Yes	Yes
Formal screening of search results against eligibility criteria	Yes	No
Data extraction	Yes	No
Risk of bias (quality) assessment	Yes	No
Data analysis	No	No

Versions

18 May 2017

PROSPERO
This information has been provided by the named contact for this review. CRD has accepted this information in good faith and registered the review in PROSPERO. CRD bears no responsibility or liability for the content of this registration record, any associated files or external websites.

S2: Medline Search Strategy

Medline
1. (child* or children or childhood or kids or adolescen* or "young person*" or "young people" or teen* or youth* or boy* or girl* or juvenile).ti,ab.<br>2. exp child/<br>3. exp adolescent/<br>4. 2 or 3<br>5. (child or adolescent).ti,ab.<br>6. 1 or 5<br>7. ("physical* activ*" or "physical activity" or sport* or cycling or bicycling or bicycle* or walk* or "physical education" or "physical training" or exercis* or "energy expenditure" or danc* or "physical inactivity" or "physical fitness" or lifestyle or "active travel" or commut* or "aerobic fitness").ti,ab.<br>8. exp motor activity/<br>9. exp sports/<br>10. exp exercise/<br>11. exp physical exertion/<br>12. exp "physical education and training"/<br>13. 8 or 9 or 10 or 11 or 12<br>14. (motor activity or sports or exercise or physical exertion or "physical education and training").ti,ab.<br>15. 7 or 14<br>16. ("clinical trial" or "control* trial" or controlled or randomi#ation or randomised or randomized or randomization or randomly or randomisation or rct or "randomi#ed controlled trial*" or "randomised controlled trial" or "randomized controlled trial" or "cluster randomized controlled trial" or "group-randomized controlled trial" or "randomized controlled study" or "randomised controlled study" or "random* sample" or trial* or evaluation or effect* or control* or cluster or intervention).ti,ab.<br>17. exp randomized controlled trial/<br>18. exp clinical trial/<br>19. exp randomized controlled trials as topic/<br>20. exp clinical trial as topic/<br>21. 17 or 18 or 19 or 20<br>22. (randomized controlled trial or clinical trial or randomized controlled trials as topic or clinical trial as topic).ti,ab.<br>23. 16 or 22<br>24. ("case study" or "case report" or "abstract report" or letter).ti,ab.<br>25. exp letter/<br>26. exp historical article/<br>27. exp case report/<br>28. 25 or 26 or 27<br>29. (letter or historical article or case report).ti,ab.<br>30. 24 or 29<br>31. 23 not 30<br>32. (accelerometer or accelerometry or accelerometers or accelerometer-assessed or "counts per minute" or CPM or triaxial or Actigraph or Yamax or Actiheart or Omron, sensewear or caltrac or walk4life or ideea or actireg or lifecorder or tritrac or genea or stepwatch or actical or actiwatch or rt3 or activpal or actimarker or dynaport or CSA or MTI or pedometer or "heart rate" or pedometry or pedometers or uniaxial or actigraphy or undimensional or "objectively measur*" or "SenseWear Pro2 Armband" or "motion sensor data" or "activity monitor" or MVPA).ti,ab.<br>33. exp monitoring, ambulatory/<br>34. exp actigraphy/<br>35. 33 or 34<br>36. (monitoring, ambulatory or actigraphy).ti,ab.<br>37. 32 or 36<br>38. 6 and 15 and 31 and 37<br>39. 6 and 15 and 31 and 37

40. limit 39 to English Language

+ Year limitation: 2016 – 2017

S3: Data extracted

- Trial name
- Authors
- Publication year
- Journal of publication
- Country of implementation
- Mean age of participants
- Type of school
- Number of schools total
- Unit of randomization
- Number of clusters (Intervention group)
- Number of clusters (Control group)
- Intervention components (Education, social environment, physical environment)
- Intervention setting (School or school plus other contexts (home, community)
- Behavioural approach (Physical activity only or physical activity and other behaviours)
- Is the intervention theory based?
- What is the proposed theory?
- Duration of intervention (total weeks)
- Duration (number of sessions/week)
- MVPA accelerometer cut point
- Timing of measurements (Time 1 (Baseline), Time 2, Time 3)
- Main effect
    - Time 1: N, mean, SD for intervention and control group
    - Time 2: N, mean, SD for intervention and control group
    - Time 3: N, mean, SD for intervention and control group
- Gender effect - is the intervention targeted by gender?
    - Girls effect:
        - Time 1: N, mean, SD for intervention and control group
        - Time 2: N, mean, SD for intervention and control group
        - Time 3: N, mean, SD for intervention and control group
    - Boys effect:
        - Time 1: N, mean, SD for intervention and control group
        - Time 2: N, mean, SD for intervention and control group
        - Time 3: N, mean, SD for intervention and control group
- Socioeconomic position effect - is the intervention targeted by SEP (If yes by individual, school or community SEP)
    - Low SEP tertile
        - Time 1: N, mean, SD for intervention and control group
        - Time 2: N, mean, SD for intervention and control group
        - Time 3: N, mean, SD for intervention and control group
    - Middle SEP tertile
        - Time 1: N, mean, SD for intervention and control group
        - Time 2: N, mean, SD for intervention and control group
        - Time 3: N, mean, SD for intervention and control group
    - High SEP tertile
        - Time 1: N, mean, SD for intervention and control group
        - Time 2: N, mean, SD for intervention and control group
        - Time 3: N, mean, SD for intervention and control group
    - Two or three groups?
    - Description for SEP indicator
        - Indicator/cut off for low SEP
        - Indicator/cut off for middle SEP

- Indicator/cut off for high SEP

S4: Template of data request form utilized

Study name: ___________________

Corresponding author: _____________

Outlined in the tables below is the information required. We ask all outcomes be in mean minutes of MVPA/day (across all valid days).

Main Effect:

	Mean	N (sample size)	Std. Deviation
**Time 1 (Baseline)**			
**Intervention**			
**Control**			
**Time 2 (Follow-up 1)**			
**Intervention**			
**Control**			
**Time 3 (Follow-up 2)**			
**Intervention**			
**Control**			

Stratified by gender:

Girls

	N	Mean mins MVPA/day	Std. Deviation
**Time 1 (Baseline)**			
**Intervention**			
**Control**			
**Time 2 (Follow-up 1)**			
**Intervention**			
**Control**			
**Time 3 (Follow-up 2)**			
**Intervention**			
**Control**			

Boys

	N	Mean mins MVPA/day	Std. Deviation
**Time 1 (Baseline)**			
**Intervention**			
**Control**			
**Time 2 (Follow-up 1)**			
**Intervention**			
**Control**			
**Time 3 (Follow-up 2)**			

**Intervention**			
**Control**			

Stratified by individual indicator of Socioeconomic Status (SES)

We ask for the outcome to be presented in 3 groups (if this is not feasible, please provide based on 2 groups).

Preferentially, we would like this by indicator of 1) parental education (preferably maternal). If this is not available, we ask for the data by 2) an area-based marker of deprivation (e.g. Index of Multiple Deprivation or other postal code based indices), or alternatively 3) household income equivalised for household composition.

If this is not possible and you have other individual indicators of SES we ask you to get in touch to discuss.

SES indicator Used:
Description of indicator:
Criteria used to assign Group 1 (Low SES)
Criteria used to assign Group 2 (Middle SES)
Criteria used to assign Group 3 (High SES)

Low SES group (Group 1)

	N	Mean mins MVPA/day	Std. Deviation
**Time 1 (Baseline)**			
**Intervention**			
**Control**			
**Time 2 (Follow-up 1)**			
**Intervention**			
**Control**			
**Time 3 (Follow-up 2)**			
**Intervention**			
**Control**			

Middle SES group (Group 2)

	N	Mean mins MVPA/day	Std. Deviation
**Time 1 (Baseline)**			
**Intervention**			
**Control**			
**Time 2 (Follow-up 1)**			
**Intervention**			
**Control**			
**Time 3 (Follow-up 2)**			
**Intervention**			
**Control**			

High SES group (Group 3)

	N	Mean mins MVPA/day	Std. Deviation
**Time 1 (Baseline)**			
**Intervention**			
**Control**			
**Time 2 (Follow-up 1)**			
**Intervention**			
**Control**			
**Time 3 (Follow-up 2)**			
**Intervention**			
**Control**			

S5: Formula for imputing the standard deviation of the change

$$SD_{E,change} = \sqrt{SD^2_{E,baseline} + SD^2_{E,final} - (2 \times Corr \times SD_{E,baseline} \times SD_{E,final})},$$

S6: Trials excluded in full text screening

Trial	Citation	Reason for exclusion
Action 3:30	Jago, R., Sebire, S. J., Davies, B., Wood, L., Edwards, M. J., Banfield, K., … J.E., P. (2014). Randomised feasibility trial of a teaching assistant led extracurricular physical activity intervention for 9 to 11 year olds: Action 3:30. The International Journal of Behavioral Nutrition and Physical Activity, 11(1), no pagination–no pagination. Retrieved from http://www.ijbnpa.org/content/11/1/114	Feasibility/ pilot
Beat the Street	Coombes E, Jones A. Gamification of active travel to school: A pilot evaluation of the Beat the Street physical activity intervention. Heal Place [Internet]. 2016;39:62–9. Available from: http://dx.doi.org/10.1016/j.healthplace.2016.03.001	Feasibility/ pilot
Bristol Girls Feasibility Trial	Jago, R., Edwards, M. J., Sebire, S. J., Tomkinson, K., Bird, E. L., Banfield, K., … J.E., P. (2015). Effect and cost of an after-school dance programme on the physical activity of 11-12 year old girls: The Bristol Girls Dance Project, a school-based cluster randomised controlled trial Jago R. International Journal of Behavioral Nutrition and Physical Activity, 12(1), no pagination–no pagination. http://doi.org/10.1186/s12966-015-0289-y	Feasibility/ pilot
Couch Potatoes to Jumping Beans	Mhurchu, C. N., Maddison, R., Jiang, Y., Jull, A., Prapavessis, H., & Rodgers, A. (2008). Couch potatoes to jumping beans: A pilot study of the effect of active video games on physical activity in children. International Journal of Behavioral Nutrition and Physical Activity, 5(8). http://doi.org/10.1186/1479	Feasibility/ pilot
Crouter (2015)	Crouter, S. E., de Ferranti, S. D., Whiteley, J., Steltz, S. K., Osganian, S. K., Feldman, H. A., & Hayman, L. L. (2015). Effect on physical activity of a randomized afterschool intervention for Inner City Children in 3rd to 5th grade. PLoS ONE, 10(10), e0141584–e0141584. Retrieved from http://ovidsp.ovid.com/ovidweb.cgi?T=JS&PAGE=reference&D=prem&NEWS=N&AN=26510013	Feasibility/ pilot
Dudley (2010)	Dudley, D. A., Okely, A. D., Pearson, P., & Peat, J. (2010). Engaging adolescent girls from linguistically diverse and low income backgrounds in school sport: A pilot randomised controlled trial. Journal of Science and Medicine in Sport, 13(2), 217–224. http://doi.org/10.1016/j.jsams.2009.04.008	Feasibility/ pilot
EASY Minds	Riley, N., Lubans, D. R., Morgan, P. J., & Young, M. (2015). Outcomes and process evaluation of a programme integrating physical activity into the primary school mathematics curriculum: The EASY Minds pilot randomised controlled trial. Journal of Science and Medicine in Sport / Sports Medicine Australia, 18(6), 656–661. Retrieved from http://ovidsp.ovid.com/ovidweb.cgi?T=JS&PAGE=reference&D=prem&NEWS=N&AN=25304445	Feasibility/ pilot

Fit4fun Pilot Study	Eather N, Morgan PJ, Lubans DR. Feasibility and preliminary efficacy of the Fit4Fun intervention for improving physical fitness in a sample of primary school children: a pilot study. Phys Educ Sport Pedagog. 2013;18(4):389–411.	Feasibility/ pilot
Hands (2011)	Hands, B., Larkin, D., Rose, E., Parker, H., & Smith, A. (2011). Can Young Children Make Active Choices? Outcomes of a Feasibility Trial in Seven-Year-Old Children. Early Child Development and Care, 181(5), 625–637. Retrieved from http://search.ebscohost.com/login.aspx?direct=true&db=eric&AN=EJ923980&site=ehost-live&scope=site	Feasibility/ pilot
Healthy Homework pilot study	Duncan, S., McPhee, J. C., Schluter, P. J., Zinn, C., Smith, R., & Schofield, G. (2011). Efficacy of a compulsory homework programme for increasing physical activity and healthy eating in children: The Healthy Homework pilot study. The International Journal of Behavioral Nutrition and Physical Activity, 8, no pagination–no pagination. http://doi.org/10.1186/1479-5868-8-127	Feasibility/ pilot
Maloney (2008)	Maloney, A. E., Bethea, T. C., Kelsey, K. S., Marks, J. T., Paez, S., Rosenberg, A. M., … Sikich, L. (2008). A pilot of a video game (DDR) to promote physical activity and decrease sedentary screen time. Obesity, 16(9), 2074–2080. http://doi.org/10.1038/oby.2008.295	Feasibility/ pilot
Memphis GEMS Pilot Trial	Beech, B. M., Klesges, R. C., Kumanyika, S. K., Murray, D. M., Klesges, L., McClanahan, B., … B., M.-A. M. M.-A. M. (2003). Child- and parent-targeted interventions: the Memphis GEMS pilot study. Ethnicity & Disease, 13(1 Suppl 1), S1–53. Retrieved from http://ovidsp.ovid.com/ovidweb.cgi?T=JS&PAGE=reference&D=emed6&NEWS=N&AN=2003168621	Feasibility/ pilot
Minnesota GEMS Pilot Study	Story, M., Sherwood, N. E., Himes, J. H., Davis, M., Jacobs, D. R., Cartwright, Y., … Rochon, J. (2003). An After-school obesity prevention program for africian-american girls: The Minnesota GEMS Pilot Study. Ethnicity & Disease, 13.	Feasibility/ pilot
Reznik (2015)	Reznik, M., Wylie-Rosett, J., Kim, M., & Ozuah, P. O. (2015). A classroom-based physical activity intervention for urban kindergarten and first-grade students: A feasibility study. Childhood Obesity, 11(3), 314–324. http://doi.org/10.1089/chi.2014.0090	Feasibility/ pilot
Robbins (2012)	Robbins, L. B., Pfeiffer, K. A., Maier, K. S., Lo, Y.-J., & Wesolek, S. M. (2012). Pilot Intervention to Increase Physical Activity Among Sedentary Urban Middle School Girls: A Two-Group Pretest-Posttest Quasi-Experimental Design. Journal of School Nursing, 28(4), 302–315. http://doi.org/10.1177/1059840512438777	Feasibility/ pilot
The EASY Minds pilot RCT	Riley, N., Lubans, D. R., Morgan, P. J., & Young, M. (2015). Outcomes and process evaluation of a programme integrating physical activity into the primary school mathematics curriculum: The EASY Minds pilot randomised controlled trial. Journal of Science and Medicine in Sport / Sports Medicine Australia, 18(6), 656–661. Retrieved from	Feasibility/ pilot

	http://ovidsp.ovid.com/ovidweb.cgi?T=JS&PAGE=reference&D=prem&NEWS=N&AN=25304445	
Walking School Bus - Texas (Mendoza et al. 2011)	Mendoza, J., Watson, K., Baranowski, T., Nicklas, T., Uscanga, D., Hanfling, M. (2011). Pediatrics	Feasibility/ pilot
Wilson (2005)	Wilson, D. K., Evans, A. E., Williams, J., Mixon, G., Sirard, J. R., Pate, R., … J.R., S. (2005). A preliminary test of a student-centered intervention on increasing physical activity in underserved adolescents. Annals of Behavioral Medicine, 30(2), 119–124. http://doi.org/10.1207/s15324796abm3002_4	Feasibility/ pilot
Van Kann et al. (2016)	Van Kann D, Kremers S, de Vries N, de Vries S. The effect of a school-centered multicomponent intervention on daily physical activity and sedentary behavior in primary school children: The Active Living study. Prev Med (Baltim) [Internet]. 2016;89:64–9.	Interventi on design
Prochaska (2004)	Prochaska, J. J., & Sallis, J. F. (2004). A Randomized Controlled Trial of Single Versus Multiple Health Behavior Change: Promoting Physical Activity and Nutrition Among Adolescents. Health Psychology, 23(3), 314–318. http://doi.org/10.1037/0278-6133.23.3.314	Interventi on design
Beets et al. (2016)	Beets, M. W., Weaver, R. G., Turner-McGrievy, G., Huberty, J., Ward, D. S., Pate, R. R., … Beighle, A. (2015). Making policy practice in afterschool programs: A randomized controlled trial on physical activity changes. American Journal of Preventive Medicine, 48(6), 694–706. http://doi.org/10.1016/j.amepre.2015.01.012	Outcome (Not full day)
Cradock et al. (2016)	Cradock, A. L., Barrett, J. L., Giles, C. M., Lee, R. M., Kenney, E. L., deBlois, M. E., … Gortmaker, S. L. (2016). Promoting Physical Activity With the Out of School Nutrition and Physical Activity (OSNAP) Initiative: A Cluster-Randomized Controlled Trial. JAMA Pediatrics, 170(2), 155–162. Retrieved from http://ovidsp.ovid.com/ovidweb.cgi?T=JS&PAGE=reference&D=prem&NEWS=N&AN=26641557	Outcome (Not full day)
Van Kann et al. (2016)	Van Kann DHH, de Vries SI, Schipperijn J, de Vries NK, Jansen MWJ, Kremers SPJ. A Multicomponent Schoolyard Intervention Targeting Children’s Recess Physical Activity and Sedentary Behavior: Effects After One Year. J Phys Act Health [Internet]. 2016;1–28.	Outcome (Not full day)
It's child's play	Engelen, L., Bundy, A. C., Naughton, G., Simpson, J. M., Bauman, A., Ragen, J., … van der Ploeg, H. P. (2013). Increasing physical activity in young primary school children—It’s child's play: A cluster randomised controlled trial. Preventive Medicine: An International Journal Devoted to Practice and Theory, 56(5), 319–325. http://doi.org/10.1016/j.ypmed.2013.02.007	Outcome (Not full day)

Martin et al. (2016)	Martins S, Palmeira A, Minderico C. Longitudinal outcomes of a school-based lifestyle promotion program: Preliminary results [Internet]. Journal of Adolescent Health. Elsevier USA; 2011. p. S79–S79. Available from: http://ovidsp.ovid.com/ovidweb.cgi?T=JS&PAGE=reference&D=emed10&NEWS=N&AN=70352287	Outcome (Not full day)
STAR Programme	Ha, A. S., Burnett, A., Sum, R., Medic, N., & Ng, J. Y. Y. (2015). Outcomes of the Rope Skipping "STAR" Programme for Schoolchildren. Journal of Human Kinetics, 45, 233–240. http://doi.org/10.1515/hukin-2015-0024	Outcome (Not full day)
STOPP	Marcus, C., Nyberg, G., Nordenfelt, A., Karpmyr, M., Kowalski, J., & Ekelund, U. (2009). A 4-year, cluster-randomized, controlled childhood obesity prevention study: STOPP. International Journal of Obesity, 33(4), 408–417. http://doi.org/10.1038/ijo.2009.38	Outcome (Not full day)
Weaver et al. (2016)	Weaver RG, Moore JB, Huberty J, Freedman D, Turner-McGrievy B, Beighle A, et al. Process Evaluation of Making HEPA Policy Practice: A Group Randomized Trial. Health Promot Pract. 2016;17(5):631–47.	Outcome (Not full day)
Wells (2014)	Wells, N. M., Myers, B. M., & Henderson Jr., C. R. (2014). School gardens and physical activity: A randomized controlled trial of low-income elementary schools. Preventive Medicine, 69, Supple, S27–S33. http://doi.org/10.1016/j.ypmed.2014.10.012	Outcome (Not full day)
Aburto (2011)	Aburto, N. J., Fulton, J. E., Safdie, M., Duque, T., Bonvecchio, A., & Rivera, J. A. (2011). Effect of a school-based intervention on physical activity: Cluster-randomized trial. Medicine and Science in Sports and Exercise, 43(10), 1898–1906. http://doi.org/10.1249/MSS.0b013e318217ebec	Outcome (Not via accelerometer)
Eyre (2016)	Eyre, E. L. J., Cox, V. M., Birch, S. L., & Duncan, M. J. (2016). An integrated curriculum approach to increasing habitual physical activity in deprived South Asian children. European Journal of Sport Science, 16(3), 381–390. http://doi.org/10.1080/17461391.2015.1062565	Outcome (Not via accelerometer)
FATaintPHAT	Ezendam, N. P. M., Brug, J., Oenema, A., JJ, R., I, A., PM, G., … I, D. B. (2012). Evaluation of the Web-Based Computer-Tailored FATaintPHAT Intervention to Promote Energy Balance Among Adolescents. Archives of Pediatrics & Adolescent Medicine, 166(3), 248. http://doi.org/10.1001/archpediatrics.2011.204	Outcome (Not via accelerometer)
Fit 'n' fun dudes program (2009)	Hardman, C. A., Horne, P. J., & Lowe, C. F. (2009). A home-based intervention to increase physical activity in girls: The fit "n" fun dudes program. Journal of Exercise Science and Fitness, 7(1), 1–8. http://doi.org/10.1016/S1728-869X(09)60001-0	Outcome (Not via accelerometer)
Fit 'n' fun dudes program (2011)	Hardman, C. A., Horne, P. J., & Lowe, C. F. (2011). Effects of rewards, peer-modelling and pedometer targets on children's physical activity: A school-based intervention study. Psychology and Health, 26(1), 3–21. http://doi.org/10.1080/08870440903318119	Outcome (Not via accelerometer)

Fit4fun Trial	Eather, N., Morgan, P. J., & Lubans, D. R. (2013a). Feasibility and preliminary efficacy of the Fit4Fun intervention for improving physical fitness in a sample of primary school children: a pilot study. Physical Education and Sport Pedagogy, 18(4), 389–411. http://doi.org/10.1080/17408989.2012.690375	Outcome (Not via accelerom eter)
Harder-lauridsen (2014)	Harder-lauridsen, N. M., Birk, N. M., Ried-larsen, M., Juul, A., & Andersen, L. B. (2014). A randomized controlled trial on a multicomponent intervention for overweight school-aged children - Copenhagen, Denmark. BMC Pediatrics, 273(14), 1–14.	Outcome (Not via accelerom eter)
lauft' trial	210. Suchert, V., Isensee, B., Sargent, J., Weisser, B., Hanewinkel, R., & Group, lauft. S. (2015). Prospective effects of pedometer use and class competitions on physical activity in youth: A cluster-randomized controlled trial. Preventive Medicine, 81, 399–404.	Outcome (Not via accelerom eter)
Lee (2012)	Lee, L., Kuo, Y., Fanaw, D., Perng, S., & Juang, I. (2012). The effect of an intervention combining self efficacy theory and pedometers on promoting physical activity among adolescents. Journal of Clinical Nursing, 21(7-8), 914–922. http://doi.org/10.1111/j.1365-2702.2011.03881.x	Outcome (Not via accelerom eter)
Lubans & Morgan (2008)	Lubans, D., & Morgan, P. (2008). Evaluation of an extra-curricular school sport programme promoting lifestyle and lifetime activity for adolescents. Journal of Sports Sciences, 26(5), 519–529. http://doi.org/10.1080/02640410701624549	Outcome (Not via accelerom eter)
MacConnie (1982)	MacConnie, S. E., T.B., G., D.L., G., & A.E., P. I. I. I. (1982). Daily physical activity patterns of prepubertal children involved in a vigorous exercise program. International Journal of Sports Medicine, 3(4), 202–207. Retrieved from http://ovidsp.ovid.com/ovidweb.cgi?T=JS&PAGE=reference&D=emed1a&NEWS=N&AN=1983072947	Outcome (Not via accelerom eter)
McManus (2008)	McManus, A. M., Masters, R. S. W., Laukkanen, R. M. T., Yu, C. C. W., Sit, C. H. P., & Ling, F. C. M. (2008). Using heart-rate feedback to increase physical activity in children. Preventive Medicine, 47(4), 402–8. http://doi.org/10.1016/j.ypmed.2008.06.001	Outcome (Not via accelerom eter)
Physical Activity Leaders (PALS)	Lubans, D. R., Morgan, P. J., Aguiar, E. J., & Callister, R. (2011). Randomized controlled trial of the Physical Activity Leaders (PALs) program for adolescent boys from disadvantaged secondary schools. Preventive Medicine, 52(3-4), 239–246. http://doi.org/10.1016/j.ypmed.2011.01.009	Outcome (Not via accelerom eter)
PLAY	Pangrazi, R. P., Beighle, A., Vehige, T., Vack, C., R.P., P., A., B., & T., V. (2003). Impact of Promoting Lifestyle Activity for Youth (PLAY) on children's physical activity. Journal of School Health, 73(8), 317–321. Retrieved from http://ovidsp.ovid.com/ovidweb.cgi?T=JS&PAGE=reference&D=emed6&NEWS=N&AN=14593948	Outcome (Not via accelerom eter)

Program X	Lubans, D. R., Morgan, P. J., Callister, R., Collins, C. E., & Plotnikoff, R. C. (2010). Exploring the mechanisms of physical activity and dietary behavior change in the program x intervention for adolescents. The Journal of Adolescent Health : Official Publication of the Society for Adolescent Medicine, 47(1), 83–91. http://doi.org/10.1016/j.jadohealth.2009.12.015	Outcome (Not via accelerom eter)
Reza (2014)	Reza, S., Tahir, W. M., Zakaria, W., Agency, M. N., & Agency, N. (2014). Impact of Social-Ecological Intervention on Physical Activity Knowledge and Behaviors of Rural Students. Journal of Physical Activity and Health.	Outcome (Not via accelerom eter)
Schodielf (2005)	Schofield, L., Mummery, W. K., & Schofield, G. (2005). Effects of a controlled pedometer-intervention trial for low-active adolescent girls. Medicine and Science in Sports and Exercise, 37(8), 1414–1420. Retrieved from http://ovidsp.ovid.com/ovidweb.cgi?T=JS&PAGE=reference&D=emed7&NEWS=N&AN=2005390591	Outcome (Not via accelerom eter)
Shore (2014) [HYPPE]	Shore, S. M., Sachs, M. L., DuCette, J. P., & Libonati, J. R. (2014). Step-Count Promotion Through a School-Based Intervention. Clinical Nursing Research, 23(4), 402–420. http://doi.org/10.1177/1054773813485240	Outcome (Not via accelerom eter)
SWITCH what you Do, View and Chew	Gentile, D. A., Welk, G., Eisenmann, J. C., Reimer, R. A., Walsh, D. A., Russell, D. W., … S., S. (2009). Evaluation of a multiple ecological level child obesity prevention program: Switch what you Do, View, and Chew. BMC Medicine, 7, 49. http://doi.org/10.1186/1741-7015-7-49	Outcome (Not via accelerom eter)
AIMFIT Pragmatic Randomiz ed Controlle d Trial	Direito, A., Jiang, Y., Whittaker, R., & Maddison, R. (2015). Apps for IMproving FITness and Increasing Physical Activity Among Young People: The AIMFIT Pragmatic Randomized Controlled Trial. Journal of Medical Internet Research, 17(8), e210–e210. http://doi.org/10.2196/jmir.4568	Setting (not centrally school based)
Backlund (2011)	Bäcklund, C., Sundelin, G., & Larsson, C. (2011). Effects of a 2-year lifestyle intervention on physical activity in overweight and obese children. Advances in Physiotherapy, 13(3), 97–109. http://doi.org/10.3109/14038196.2011.562540	Setting (not centrally school based)
Baranows ki (2011)	Baranowski, T., Baranowski, J., Thompson, D., Buday, R., Jago, R., Griffith, M. J., … Watson, K. B. (2011). Video game play, child diet, and physical activity behavior change: A randomized clinical trial. American Journal of Preventive Medicine, 40(1), 33–38. http://doi.org/10.1016/j.amepre.2010.09.029	Setting (not centrally school based)
Baranows ki (2012)	Baranowski, T., Abdelsamad, D., Baranowski, J., O'Connor, T. M., Thompson, D., Barnett, A., … Chen, T.-A. (2012). Impact of an active video game on healthy children's physical activity. Pediatrics, 129(3), e636–e642. Retrieved from http://ovidsp.ovid.com/ovidweb.cgi?T=JS&PAGE=reference&D=medl&NEWS=N&AN=22371457	Setting (not centrally school based)

BOUNCE	Olvera, N., Bush, J. A., Sharma, S. V, Knox, B. B., Scherer, R. L., & Butte, N. F. (2010). BOUNCE: A community-based mother-daughter healthy lifestyle intervention for low-income Latino families. Obesity, 18(Suppl 1), S102–S104. http://doi.org/10.1038/oby.2009.439	Setting (not centrally school based)
Challenge !	Black, M. M., Hager, E. R., Le, K., Anliker, J., Arteaga, S. S., DiClemente, C., … Wang, Y. (2010). Challenge! Health promotion/obesity prevention mentorship model among urban, Black adolescents. Pediatrics, 126(2), 280–288. http://doi.org/10.1542/peds.2009-1832	Setting (not centrally school based)
Chen (2010)	Chen, J. L., Weiss, S., Heyman, M. B., & Lustig, R. H. (2010). Efficacy of a child-centred and family-based program in promoting healthy weight and healthy behaviors in Chinese American children: A randomized controlled study. Journal of Public Health, 32(2), 219–229. http://doi.org/10.1093/pubmed/fdp105	Setting (not centrally school based)
CPET	Morrison, R., Reilly, J. J., Penpraze, V., Westgarth, C., Ward, D. S., Mutrie, N., … Yam, P. S. (2013). Children, parents and pets exercising together (CPET): exploratory randomised controlled trial. BMC Public Health, 13, 1096. Retrieved from http://ovidsp.ovid.com/ovidweb.cgi?T=JS&PAGE=reference&D=medl&NEWS=N&AN=24279294	Setting (not centrally school based)
Finkelstei n (2013)	Finkelstein, E. A., Y.-T., T., R., M., C.-F., L., S.-S., G., Finkelstein, E. A., … Saw, S.-M. (2013). A cluster randomized controlled trial of an incentive-based outdoor physical activity program. The Journal of Pediatrics, 163(1), 167–72.e1. Retrieved from http://ovidsp.ovid.com/ovidweb.cgi?T=JS&PAGE=reference&D=medl&NEWS=N&AN=23415616	Setting (not centrally school based)
Fit for Life Boy Scouts Program	Jago, R., Baranowski, T., Baranowski, J. C., Thompson, D., Cullen, K. W., Watson, K., & Liu, Y. (2006). Fit for Life Boy Scout badge: Outcome evaluation of a troop and Internet intervention. Preventive Medicine: An International Journal Devoted to Practice and Theory, 42(3), 181–187. http://doi.org/10.1016/j.ypmed.2005.12.010	Setting (not centrally school based)
Gortmake r (2012)	Gortmaker, S. L., Lee, R. M., Mozaffarian, R. S., Sobol, A. M., Nelson, T. F., Roth, B. A., & Wiecha, J. L. (2012). Effect of an after-school intervention on increases in children's physical activity. Medicine and Science in Sports and Exercise, 44(3), 450–457. http://doi.org/10.1249/MSS.0b013e3182300128	Setting (not centrally school based)
Graves (2010)	Graves, L., Ridgers, N., Atkinson, G., Stratton, G. (2010). The Effect of Active Video Gaming on Children's Physical Activity , Behavior Preferences and Body Composition. Pediatric Excercise Science, 22(April 2016), 535–546.	Setting (not centrally school based)
Healthy Dads, Healthy	Morgan, P. J., Lubans, D. R., Callister, R., Okely, A. D., Burrows, T. L., Fletcher, R., & Collins, C. E. (2011). The Healthy Dads, Healthy Kids randomized controlled trial: Efficacy of a healthy lifestyle program for overweight fathers and their children. International Journal of Obesity	Setting (not centrally

Kids (2011)	(2005), 35(3), 436–447. Retrieved from http://ovidsp.ovid.com/ovidweb.cgi?T=JS&PAGE=reference&D=med5&NEWS=N&AN=20697417	school based)
Healthy Dads, Healthy Kids (2014)	Morgan, P. J., Collins, C. E., Plotnikoff, R. C., Callister, R., Burrows, T., Fletcher, R., … Lubans, D. R. (2014). The "Healthy Dads, Healthy Kids" community randomized controlled trial: A community-based healthy lifestyle program for fathers and their children. Preventive Medicine: An International Journal Devoted to Practice and Theory, 61, 90–99. http://doi.org/10.1016/j.ypmed.2013.12.019	Setting (not centrally school based)
Laukkanen (2015)	Laukkanen, A., Juhani Pesola, A., Heikkinen, R., Kaarina Sääkslahti, A., & Finni, T. (2015). Family-based cluster randomized controlled trial enhancing physical activity and motor competence in 4-7-year-old children. PLoS ONE, 10(10), no pagination–no pagination. http://doi.org/10.1371/journal.pone.0141124	Setting (not centrally school based)
Lifestyle triple P	Gerards, S. M. P. L., Dagnelie, P. C., Gubbels, J. S., van Buuren, S., Hamers, F. J. M., Jansen, M. W. J., … Kremers, S. P. J. (2015). The effectiveness of lifestyle triple P in the Netherlands: a randomized controlled trial. PloS One, 10(4), e0122240–e0122240. Retrieved from http://ovidsp.ovid.com/ovidweb.cgi?T=JS&PAGE=reference&D=medl&NEWS=N&AN=25849523	Setting (not centrally school based)
Memphis GEMS	Klesges, R., Obarzanek, E., Kumanyika, S., Murray, D., Klesges, L., Relyea, G., … Slawson, D. L. (2014). The Memphis Girls' health Enrichment Multi-site Studies (GEMS). Archives of Pediatrics and Adolescent Medicine, 164(11), 1007–1014.	Setting (not centrally school based)
Nereu program	Serra-Paya, N., Ensenyat, A., Castro-Vinuales, I., Real, J., Sinfreu-Bergues, X., Zapata, A., … Teixido, C. (2015). Effectiveness of a Multi-Component Intervention for Overweight and Obese Children (Nereu Program): A Randomized Controlled Trial. PloS One, 10(12), e0144502–e0144502. Retrieved from http://ovidsp.ovid.com/ovidweb.cgi?T=JS&PAGE=reference&D=prem&NEWS=N&AN=26658988	Setting (not centrally school based)
PACE+	Patrick, K., KJ, C., GJ, N., & al, et. (2006). Randomized controlled trial of a primary care and home-based intervention for physical activity and nutrition behaviors: Pace+ for adolescents. Archives of Pediatrics & Adolescent Medicine, 160(2), 128–136. http://doi.org/10.1001/archpedi.160.2.128	Setting (not centrally school based)
Roemmich (2004)	Roemmich, J. N., Gurgol, C. M., & Epstein, L. H. (2004). Open-Loop Feedback Increases Physical Activity of Youth. Medicine & Science in Sports & Exercise, 36(4), 668–673. http://doi.org/10.1249/01.MSS.0000121947.59529.3B	Setting (not centrally school based)
Roemmich (2012)	Roemmich, J. N., Lobarinas, C. L., Barkley, J. E., White, T. M., Paluch, R., & Epstein, L. H. (2012). Use of an open-loop system to increase physical activity. Pediatric Exercise Science, 24(3), 384–398. Retrieved from	Setting (not centrally

	http://ovidsp.ovid.com/ovidweb.cgi?T=JS&PAGE=reference&D=medl&NEWS=N&AN=22971555	school based)
School-Community Partnerships	Madsen, K., Thompson, H., Adkins, A., & Crawford, Y. (2013). School-Community Partnerships: A Cluster-Randomized Trial of an After-School Soccer Program. JAMA Pediatrics, 167(4), 321. http://doi.org/10.1001/jamapediatrics.2013.1071	Setting (not centrally school based)
Straker (2013)	Straker, L. M., Abbott, R. A., & Smith, A. J. (2013). To remove or to replace traditional electronic games? A crossover randomised controlled trial on the impact of removing or replacing home access to electronic games on physical activity and sedentary behaviour in children aged 10-12 years. BMJ Open, 3(6), no pagination–no pagination. http://doi.org/10.1136/bmjopen-2013-002629	Setting (not centrally school based)
The Family Project	32. Coppins, D. F., Margetts, B. M., Fa, J. L., Brown, M., Garrett, F., & Huelin, S. (2011). Effectiveness of a multi-disciplinary family-based programme for treating childhood obesity (the Family Project). European Journal of Clinical Nutrition, 65(8), 903–909. http://doi.org/10.1038/ejcn.2011.43	Setting (not centrally school based)
Web ABC study	Chen, J. L., Weiss, S., Heyman, M. B., Cooper, B., & Lustig, R. H. (2011). The efficacy of the web-based childhood obesity prevention program in Chinese American adolescents (Web ABC study). Journal of Adolescent Health, 49(2), 148–154. http://doi.org/10.1016/j.jadohealth.2010.11.243	Setting (not centrally school based)
Wilson (2002)	Wilson, D. K., Friend, R., Teasley, N., Green, S., Reaves, I. L., & Sica, D. A. (2002). Motivational versus social cognitive interventions for promoting fruit and vegetable intake and physical activity in African American adolescents. Annals of Behavioral Medicine : A Publication of the Society of Behavioral Medicine, 24(4), 310–319. Retrieved from http://ovidsp.ovid.com/ovidweb.cgi?T=JS&PAGE=reference&D=med4&NEWS=N&AN=12434942	Setting (not centrally school based)
Meinhardt (2013)	Meinhardt, U., Witassek, F., Petrò, R., Fritz, C., & Eiholzer, U. (2013). Strength training and physical activity in boys: a randomized trial. Pediatrics, 132(6), 1105–1111. http://doi.org/10.1542/peds.2013-1343	Study design (not individually randomised)
Action Schools! BC	Naylor, P.-J., Macdonald, H. M., Warburton, D. E. R., Reed, K. E., McKay, H. A., H.M., M., … K.E., R. (2008). An active school model to promote physical activity in elementary schools: Action schools! BC Naylor P.-J. British Journal of Sports Medicine, 42(5), 338–343. http://doi.org/10.1136/bjsm.2007.042036	Study design (not RCT)
APPLE	Taylor, R. W., Mcauley, K. A., Barbezat, W., Strong, A., Williams, S. M., & Mann, J. I. (2007). APPLE Project: 2-y findings of a community-based obesity prevention program in primary school – age children, (1).	Study design (not RCT)

Boston Active School Day policy	Cradock, A. L., Barrett, J. L., Carter, J., McHugh, A., Sproul, J., Russo, E. T., … Gortmaker, S. L. (2014). Impact of the Boston Active School Day policy to promote physical activity among children. American Journal of Health Promotion : AJHP, 28(3 Supplement), S54–S64. http://doi.org/10.4278/ajhp.130430-QUAN-204	Study design (not RCT)
Carson (2014)	Carson, R. L., Castelli, D. M., Pulling Kuhn, A. C., Moore, J. B., Beets, M. W., Beighle, A., … Glowacki, E. M. (2014). Impact of trained champions of comprehensive school physical activity programs on school physical activity offerings, youth physical activity and sedentary behaviors. Preventive Medicine, 69(S), S12–S19. http://doi.org/10.1016/j.ypmed.2014.08.025	Study design (not RCT)
Copenhagen School Child Intervention Study	Bugge, A., El-Naaman, B., Dencker, M., Froberg, K., Holme, I. M. K., McMurray, R. G., & Andersen, L. B. (2012). Effects of a three-year intervention: the Copenhagen School Child Intervention Study. Medicine and Science in Sports and Exercise, 44(7), 1310–1317. Retrieved from http://ovidsp.ovid.com/ovidweb.cgi?T=JS&PAGE=reference&D=medl&NEWS=N&AN=22297806	Study design (not RCT)
D'Haese (2015)	D'Haese, S., Van Dyck, D., De Bourdeaudhuij, I., Deforche, B., & Cardon, G. (2015). Organizing "Play Streets" during school vacations can increase physical activity and decrease sedentary time in children. The International Journal of Behavioral Nutrition and Physical Activity, 12(1), 171. http://doi.org/10.1186/s12966-015-0171-y	Study design (not RCT)
Dimitriou (2011)	Dimitriou, M., Michalopoulou, M., Gourgoulis, V., & Aggelousis, N. (2011). Participation in community-based sport skills learning programmes, physical activity recommendations and health-related fitness for children in Greece. Sport Sciences for Health, 6(2-3), 93–102. http://doi.org/10.1007/s11332-011-0103-4	Study design (not RCT)
Erwin (2011)	Erwin, H. E., Abel, M. G., Beighle, A., & Beets, M. W. (2011). Promoting children's health through physically active math classes: a pilot study. Health Promotion Practice, 12(2), 244–251. http://doi.org/10.1177/1524839909331911	Study design (not RCT)
Great Activity Programme	Morris, J. G., Gorely, T., Sedgwick, M. J., Nevill, A., Nevill, M. E., J.G., M., … A., N. (2013). Effect of the Great Activity Programme on healthy lifestyle behaviours in 7-11 year olds. Journal of Sports Sciences, 31(12), 1280–1293. Retrieved from http://ovidsp.ovid.com/ovidweb.cgi?T=JS&PAGE=reference&D=emed11&NEWS=N&AN=23656302	Study design (not RCT)
GreatFun2Run	Gorely, T., Morris, J. G., Musson, H., Brown, S., Nevill, A., & Nevill, M. E. (2011). Physical activity and body composition outcomes of the GreatFun2Run intervention at 20 month follow-up. International Journal of Behavioral Nutrition and Physical Activity, 8, no pagination–no pagination. http://doi.org/10.1186/1479-5868-8-74	Study design (not RCT)

IDEFICS	Verbestel, V., De Henauw, S., Barba, G., Eiben, G., Gallois, K., Hadjigeorgiou, C., … De Bourdeaudhuij, I. (2015). Effectiveness of the IDEFICS intervention on objectively measured physical activity and sedentary time in European children. Obesity Reviews : An Official Journal of the International Association for the Study of Obesity, 16 Suppl 2, 57–67. http://doi.org/10.1111/obr.12348	Study design (not RCT)
McMinn (2012)	McMinn, D., Rowe, D. A., Murtagh, S., & Nelson, N. M. (2012). The effect of a school-based active commuting intervention on children's commuting physical activity and daily physical activity. Preventive Medicine, 54(5), 316–318. http://doi.org/10.1016/j.ypmed.2012.02.013	Study design (not RCT)
Mebane on the Move Interventi on	Benjamin Neelon SE, Namenek Brouwer RJ, Østbye T, Evenson KR, Neelon B, Martinie A, et al. A Community-Based Intervention Increases Physical Activity and Reduces Obesity in School-Age Children in North Carolina. Child Obes [Internet]. 2015;11(3):297–303. Available from: http://online.liebertpub.com/doi/10.1089/chi.2014.0130	Study design (not RCT)
Sigmund (2012)	Sigmund, E., El Ansari, W., & Sigmundová, D. (2012). Does school-based physical activity decrease overweight and obesity in children aged 6-9 years? A two-year non-randomized longitudinal intervention study in the Czech Republic. BMC Public Health, 12(1), 570. http://doi.org/10.1186/1471-2458-12-570	Study design (not RCT)
Sigue la Huella	Pardo, B. M., Bengoechea, E. G., Julián Clemente, J. A., & Lanaspa, E. G. (2014). Empowering adolescents to be physically active: Three-year results of the Sigue la Huella intervention. Preventive Medicine, 66, 6–11. http://doi.org/10.1016/j.ypmed.2014.04.023	Study design (not RCT)
SPARK	Sallis, J. F., McKenzie, T. L., Alcaraz, J. E., Kolody, B., Faucette, N., & Hovell, M. F. (1997). The effects of a 2-year physical education program (SPARK) on physical activity and fitness in elementary school students. Sports, Play and Active Recreation for Kids. American Journal of Public Health, 87(8), 1328–34. http://doi.org/10.2105/AJPH.87.8.1328	Study design (not RCT)
Sport for LIFE	Breslin G, Brennan D, Rafferty R, Gallagher A. The effect of a healthy lifestyle programme on 8-9 year olds from social disadvantage. Arch Dis Child [Internet]. 2012 Jul;97(7):618–24. Available from: http://adc.bmj.com/content/97/7/618.full.pdf+html	Study design (not RCT)
Walking School Bus - Nebraska	Heelan Abbey, B., Donnelly, J., Mayo, M., Welk, G., K. (2009). Evaluation of a Walking School Bus for Promoting Physical Activity in Youth. Journal of Physical Activity and Health, (6), 560–567.	Study design (not RCT)
Y-PATH Interventi on (O'Brien et al. 2013)	O' Brien W, Issartel J, Belton S. Evidence for the Efficacy of the Youth-Physical Activity towards Health (Y-PATH) Intervention. Adv Phys Educ [Internet]. 2013;03(04):145–53. Available from: http://www.scirp.org/journal/doi.aspx?DOI=10.4236/ape.2013.34024	Study design (not RCT)

S7: Email request responses

Active by Choice Today (ACT)	Positive – data received, included
Active for Life Year 5 (AFLY5)	Positive – data received but not in appropriate form, excluded
Andrade et al. (2014)	Positive – data received, included
ATLAS	Positive – data received, included
CHANGE!	Positive – data received, included
Drummy et al. 2016	Positive – data received, included
Energy Balance 4 Kids with Play	Negative – data not received, excluded
Healthy School Start 1	Positive – data received, included
Healthy School Start 2	Positive – data received, included
HEIA Study	Positive – data received, included
IMPACT	Negative – data not received, excluded
KISS	Positive – data received, included
Magnusson et al. 2011	Negative – data not received, excluded
MOVE Project	Positive – data received, included
NEAT	Positive – data received, included
PAAC	Positive – data received but not in appropriate form, excluded
Pathways	Negative – data not received, excluded
Physical Activity 4 Everyone	Positive – data received, included
SCORES	Positive – data received, included
SPACE	Positive – data received, included
Swwitch play	Negative – data not received, excluded
The Active Smarter Kids Intervention	Positive – data received, included
The Bristol Girls Dance Project	Positive – data received, included
UP 4 FUN Pilot Intervention	Positive – data received, included
Verstrate et al 2007	Positive – data requested not available, excluded

S8: Characteristics of included studies

Active by Choice Today	
Country of implementation	USA
Mean age	11.34 (0.5)
Type of school	Middle School
Number of schools total	24
Level of cluster randomization	School
Education components?	Yes
Social environment components?	Yes
Physical environment components?	No
Behavioural approach	PA only
Theory based? If yes what theory?	Yes, Self Determination Theory
Duration of intervention total	17 weeks
Follow up 1 (months)	2.25 (mid-intervention)
Follow-up 2 (months)	4.75
Gender targeted?	No
SEP targeted?	Yes, by school SEP

Andrade et al. (2014)	
Country of implementation	Ecuador
Mean age	12.9 (0.8)
Type of school	Schools (with students in 8th or 9th year)
Number of schools total	20 (18 with accelerometer measurements)
Level of cluster randomization	School
Education components?	Yes
Social environment components?	Yes
Physical environment components?	Yes
Behavioural approach	PA and other health behaviours
Theory based? If yes what theory?	Yes, Social Cognitive Theory, Information-Motivation Behavioural Skills Model, Control Theory, Trans-Theoretical Model and Theory Of Planned Behaviour were all used
Duration of intervention total	28 months (once interrupted by 2 month annual break)
Follow up 1 (months)	24 months
Gender targeted?	No
SEP targeted?	No (but in LMIC country?)

ATLAS	
Country of implementation	Australia
Mean age	12.7 (0.5)
Type of school	Primary schools
Number of schools total	12
Level of cluster randomization	School
Education components?	Yes
Social environment components?	Yes
Physical environment components?	No
Behavioural approach	PA and other health behaviours
Theory based? If yes what theory?	Yes, Self Determination Theory and Social Cognitive Theory
Duration of intervention total	20 weeks
Follow up 1 (months)	8 months

Gender targeted?	Yes at Boys
SEP targeted?	Yes by school SEP

**CHANGE!**	
Country of implementation	UK
Mean age	10.65 (0.3)
Type of school	Primary school
Number of schools total	12
Level of cluster randomization	School
Education components?	Yes
Social environment components?	Yes
Physical environment components?	No
Behavioural approach	PA and other health behaviours
Theory based? If yes what theory?	Yes, Social Cognitive Theory
Duration of intervention total	20 weeks
Follow up 1 (months)	5 months
Follow-up 2 (months)	7.5 months
Gender targeted?	NO
SEP targeted?	No

**Drummy et al. 2016**	
Country of implementation	Northern Ireland
Mean age	9.5
Type of school	Primary school
Number of schools total	7 (14 classes)
Level of cluster randomization	Classroom
Education components?	No
Social environment components?	Yes
Physical environment components?	No
Behavioural approach	Targeting PA only
Theory based? If yes what theory?	No
Duration of intervention total	12 weeks
Follow up 1 (months)	3 months
Gender targeted?	No
SEP targeted?	No

**Healthy School Start 1**	
Country of implementation	Sweden
Mean age	6.2 (0.3)
Type of school	Pre-school class
Number of schools total	14
Level of cluster randomization	Classroom
Education components?	Yes
Social environment components?	Yes
Physical environment components?	No
Behavioural approach	PA and other health behaviours
Theory based? If yes what theory?	Yes, SCT
Duration of intervention total	24 weeks
Follow up 1 (months)	6 months
Follow up 2 (months)	12 months
Gender targeted?	No
SEP targeted?	Yes, by school and community SES

Healthy School Start 2	
Country of implementation	Sweden
Mean age	6.3 (0.3)
Type of school	Pre-school
Number of schools total	13
Level of cluster randomization	Classroom
Education components?	Yes
Social environment components?	Yes
Physical environment components?	No
Behavioural approach	Multi-behavioural
Theory based? If yes what theory?	Yes. Social Cognitive theory
Duration of intervention total	6
Follow up 1 (months)	6
Follow up 2 (months)	11
Gender targeted?	No
SEP targeted?	Yes

HEIA Study	
Country of implementation	Norway
Mean age	11.2 (0.3)
Type of school	Primary schools
Number of schools total	37
Level of cluster randomization	Classoom
Education components?	Yes
Social environment components?	Yes
Physical environment components?	Yes
Behavioural approach	Multi-behavioural
Theory based? If yes what theory?	Yes. Social Ecological Framework
Duration of intervention total	5
Follow up 1 (months)	20
Gender targeted?	No
SEP targeted?	No

KISS	
Country of implementation	Switzerland
Mean age	9.25 (0.43)
Type of school	Elementary
Number of schools total	15
Level of cluster randomization	Classroom
Education components?	Yes
Social environment components?	Yes
Physical environment components?	No
Behavioural approach	Targeting PA only
Theory based? If yes what theory?	Yes. Social Ecological Theory
Duration of intervention total	9
Follow up 1 (months)	9
Follow up 2 (months)	36
Gender targeted?	No
SEP targeted?	No

MOVE Project	
Country of implementation	UK
Mean age	11.8 (0.5)
Type of school	Secondary schools
Number of schools total	60
Level of cluster randomization	Schools
Education components?	No
Social environment components?	Yes
Physical environment components?	No
Behavioural approach	Targeting PA only
Theory based? If yes what theory?	No.
Duration of intervention total	1.5
Follow up 1 (months)	3
Gender targeted?	No
SEP targeted?	No

NEAT	
Country of implementation	Australia
Mean age	13.2 (0.5)
Type of school	Secondary school
Number of schools total	12
Level of cluster randomization	Yes
Education components?	Yes
Social environment components?	Yes
Physical environment components?	No
Behavioural approach	Mulit-behavioural
Theory based? If yes what theory?	Yes. Social cognitive theory
Duration of intervention total	12
Follow up 1 (months)	12
Follow up 2 (months)	24
Gender targeted?	Yes
SEP targeted?	Yes

Physical Activity 4 Everyone	
Country of implementation	Austalia
Mean age	12.0
Type of school	Secondary schools
Number of schools total	10
Level of cluster randomization	School
Education components?	Yes
Social environment components?	Yes
Physical environment components?	No
Behavioural approach	Targeting PA only
Theory based? If yes what theory?	Yes. Social Cognitive Theory and Ecological Theory
Duration of intervention total	24
Follow up 1 (months)	24
Gender targeted?	No
SEP targeted?	

SCORES	
Country of implementation	Australia
Mean age	8.5 (0.6)

Type of school	Primary schools
Number of schools total	8
Level of cluster randomization	Schools
Education components?	Yes
Social environment components?	Yes
Physical environment components?	No
Behavioural approach	Targeting PA only
Theory based? If yes what theory?	Yes. Self-Determination Theory and Competence Motivation Theory
Duration of intervention total	12
Follow up 1 (months)	12
Gender targeted?	No
SEP targeted?	Yes

**SPACE**	
Country of implementation	Denmark
Mean age	12.5 (0.62)
Type of school	Not specified
Number of schools total	14
Level of cluster randomization	School
Education components?	Yes
Social environment components?	Yes
Physical environment components?	Yes
Behavioural approach	Targeting PA only
Theory based? If yes what theory?	Social Ecological Model
Duration of intervention total	24
Follow up 1 (months)	24
Gender targeted?	No
SEP targeted?	No

**The Active Smarter Kids Intervention**	
Country of implementation	Norway
Mean age	10.2 (0.3)
Type of school	Elementary school
Number of schools total	60
Level of cluster randomization	School
Education components?	Yes
Social environment components?	Yes
Physical environment components?	No
Behavioural approach	Targeting PA only
Theory based? If yes what theory?	No. Social Ecological Framework
Duration of intervention total	7
Follow up 1 (months)	7
Gender targeted?	No
SEP targeted?	YEs

**The Bristol Girls Dance Project**	
Country of implementation	UK
Mean age	11.5
Type of school	Secondary schools
Number of schools total	18
Level of cluster randomization	School

Education components?	No
Social environment components?	Yes
Physical environment components?	No
Behavioural approach	Targeting PA only
Theory based? If yes what theory?	Yes. Self Determination Theory.
Duration of intervention total	5
Follow up 1 (months)	5
Follow up 2 (months)	13
Gender targeted?	Yes
SEP targeted?	No

**UP 4 Fun Pilot Intervention**	
Country of implementation	Belgium
Mean age	10.9 (0.7)
Type of school	Primary schools
Number of schools total	10
Level of cluster randomization	School
Education components?	Yes
Social environment components?	Yes
Physical environment components?	No
Behavioural approach	Targeting PA only
Theory based? If yes what theory?	Yes. Social Ecological Framework.
Duration of intervention total	1.5
Follow up 1 (months)	1.5
Gender targeted?	No
SEP targeted?	No

S9: All figures 9.1 – 9.51

Sub-contents:

9.1 Main meta-analysis, fixed effects
9.2 Main meta-analysis, random effects
9.3 Main effect subgroup analysis by behavioural approach
9.4 Main effect subgroup analysis by intervention setting
9.5 Main effect subgroup analysis by risk of bias
9.6 Main effect meta-regression by sample size
9.7 Main effect meta-regression by participant age
9.8 Main effect meta-regression by intervention duration
9.9 Main effect funnel plot and eggers test

9.10 Pooled boys and girls meta-analysis and subsequent meta-regression by gender
9. 11 Pooled SEP tertiles meta-analysis and subsequent meta-regression by SEP

9.12 Girls meta-analysis, fixed effects
9.13 Girls meta-analysis, random effects
9.14 Girls effect subgroup analysis by behavioural approach
9.15 Girls effect subgroup analysis by intervention setting
9.16 Girls effect subgroup analysis by risk of bias
9.17Girls effect meta-regression by sample size
9.18 Girls effect meta-regression by participant age
9.19 Girls effect meta-regression by intervention duration

9.20 Boys meta-analysis, fixed effects
9.21 Boys meta-analysis, random effects
9.22 Boys effect subgroup analysis by behavioural approach
9.23 Boys effect subgroup analysis by intervention setting
9.24 Boys effect subgroup analysis by risk of bias
9.25 Boys effect meta-regression by sample size
9.26 Boys effect meta-regression by participant age
9.27 Boys effect meta-regression by intervention duration

9.28 Low SEP meta-analysis, fixed effects
9.29 Low SEP meta-analysis, random effects
9.30 Low SEP effect subgroup analysis by behavioural approach
9.31 Low SEP effect subgroup analysis by intervention setting
9.32 Low SEP effect subgroup analysis by risk of bias
9.33 Low SEP effect meta-regression by sample size
9.34 Low SEP effect meta-regression by participant age
9.35 Low SEP effect meta-regression by intervention duration

9.36 Middle SEP meta-analysis, fixed effects
9.37 Middle SEP meta-analysis, random effects
9.38 Middle SEP effect subgroup analysis by behavioural approach
9.39 Middle SEP effect subgroup analysis by intervention setting
9.40 Middle SEP effect subgroup analysis by risk of bias

9.41 Middle SEP effect meta-regression by sample size
9.42 Middle SEP effect meta-regression by participant age
9.43 Middle SEP effect meta-regression by intervention duration

9.44 High SEP meta-analysis, fixed effects
9.45 High SEP meta-analysis, random effects
9.46 High SEP effect subgroup analysis by behavioural approach
9.47 High SEP effect subgroup analysis by intervention setting
9.48 High SEP effect subgroup analysis by risk of bias
9.49 High SEP effect meta-regression by sample size
9.50 High SEP effect meta-regression by participant age
9.51 High SEP effect meta-regression by intervention duration

9.1 Main meta-analysis, fixed effects

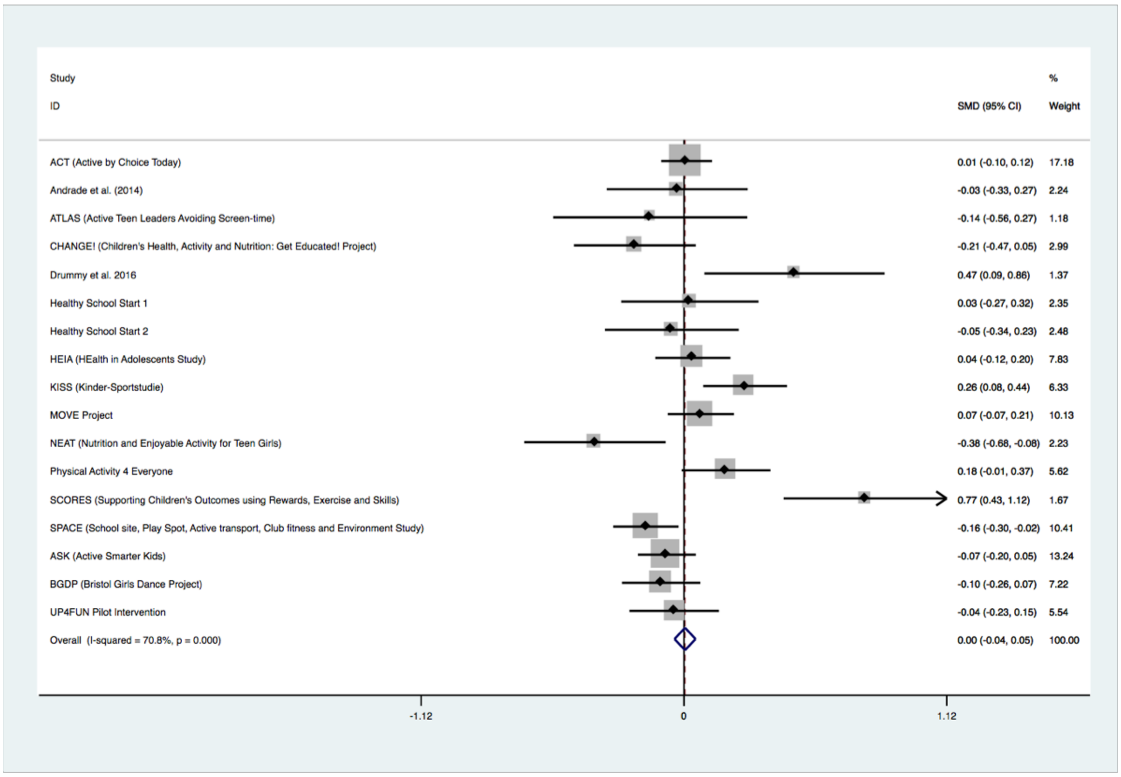

9.2 Main meta-analysis, random effects

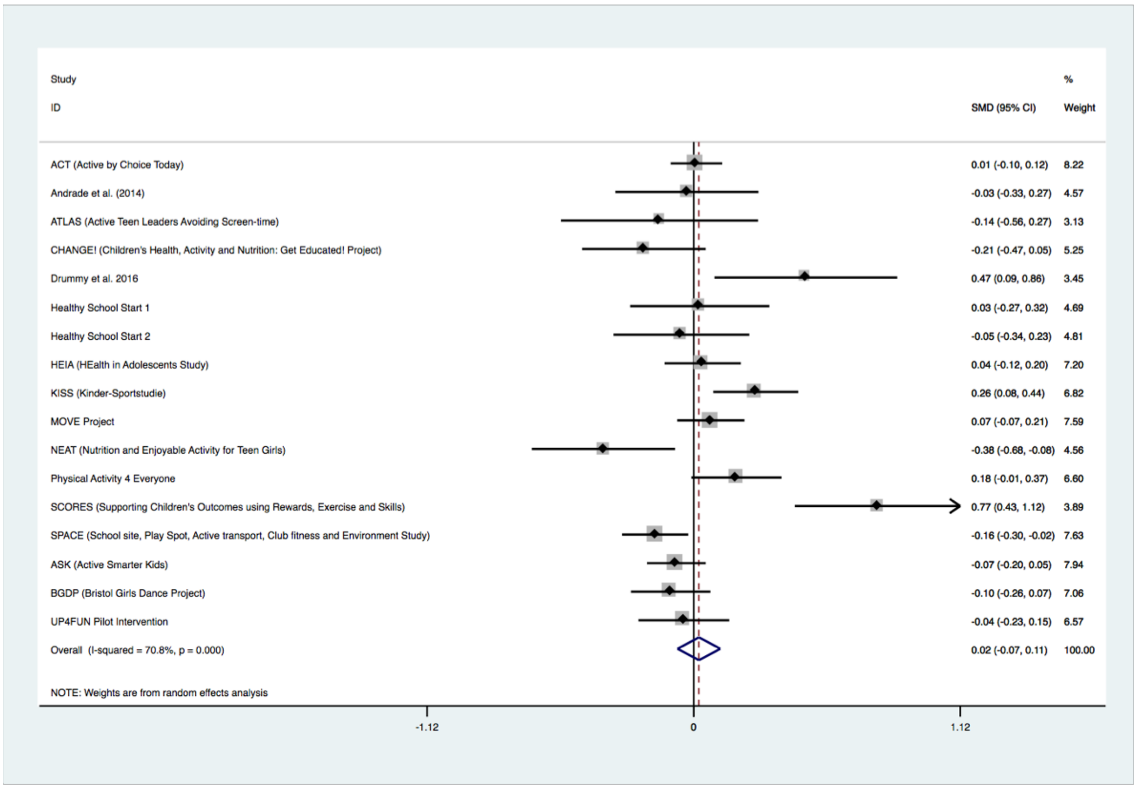

9.3 Main effect subgroup analysis by behavioural approach

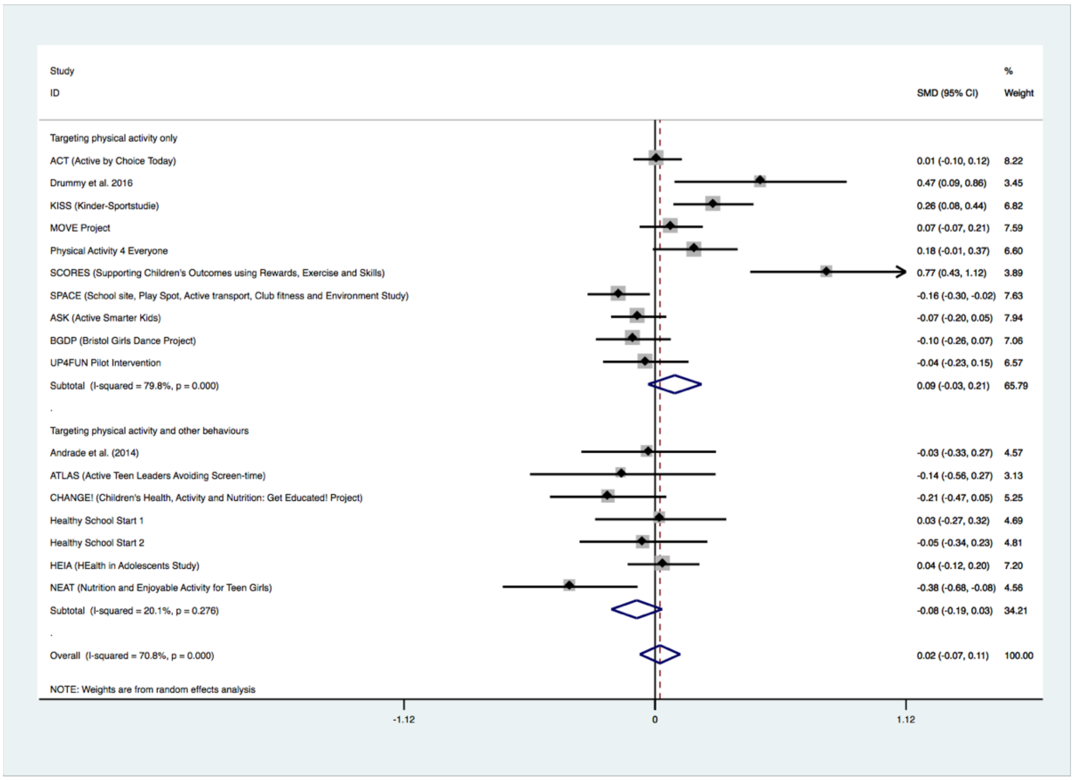

9.4 Main effect subgroup analysis by setting

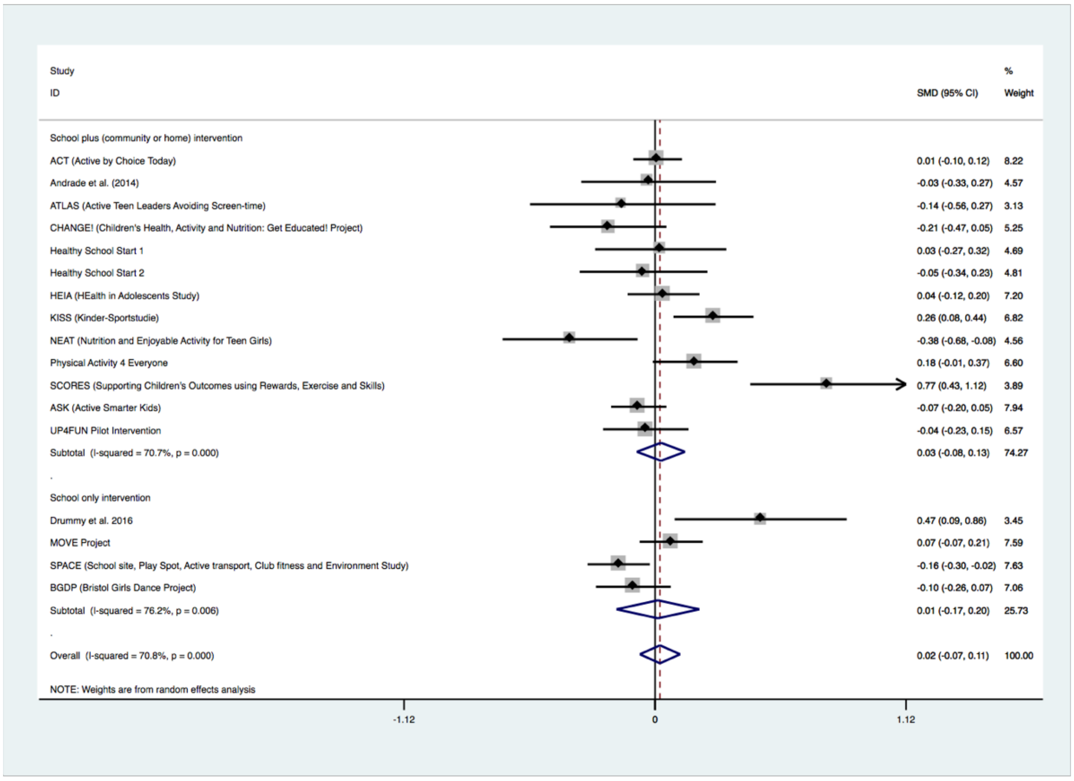

9.5 Main effect subgroup analysis by Risk of Bias Score

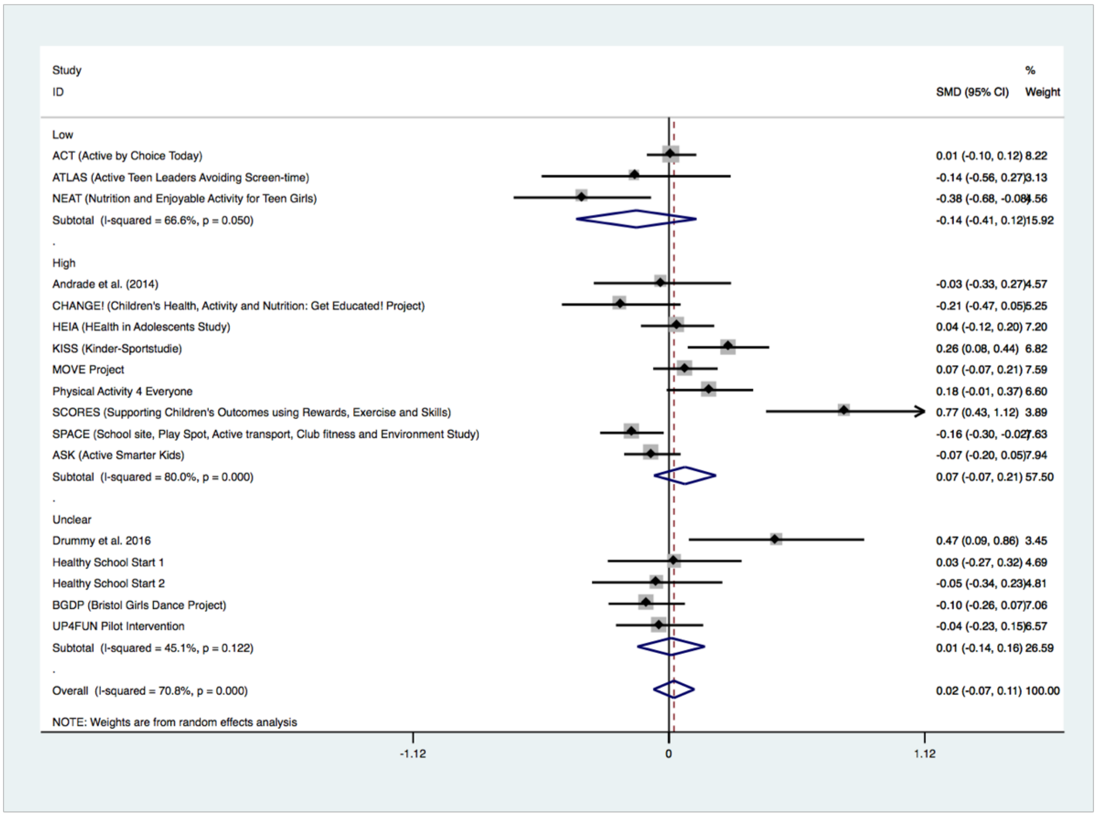

9.6 Main effect meta-regression by sample size (p-value: 0.572)

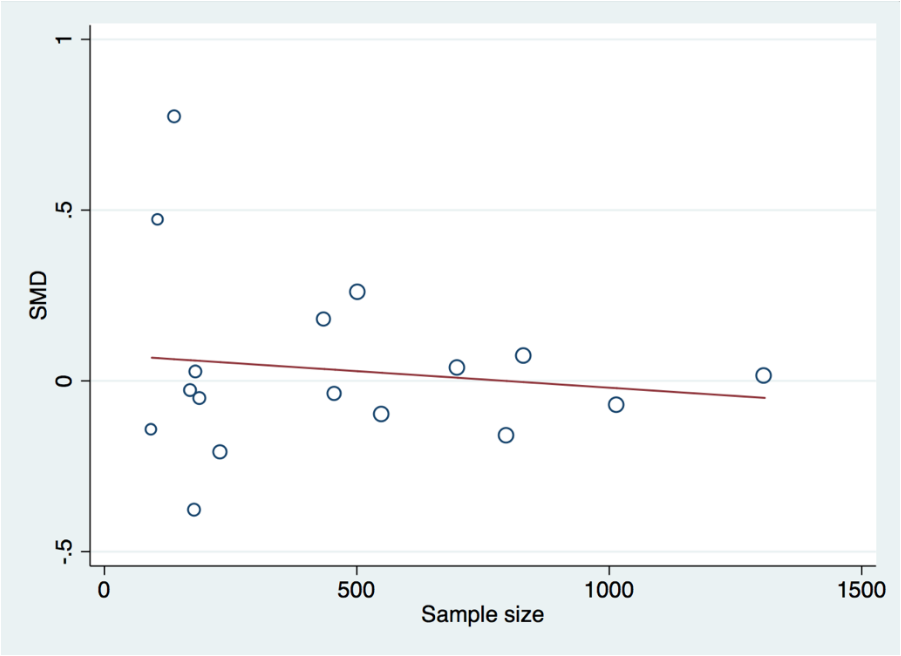

9.7 Main effect meta-regression by participant age (p-value: 0.119)

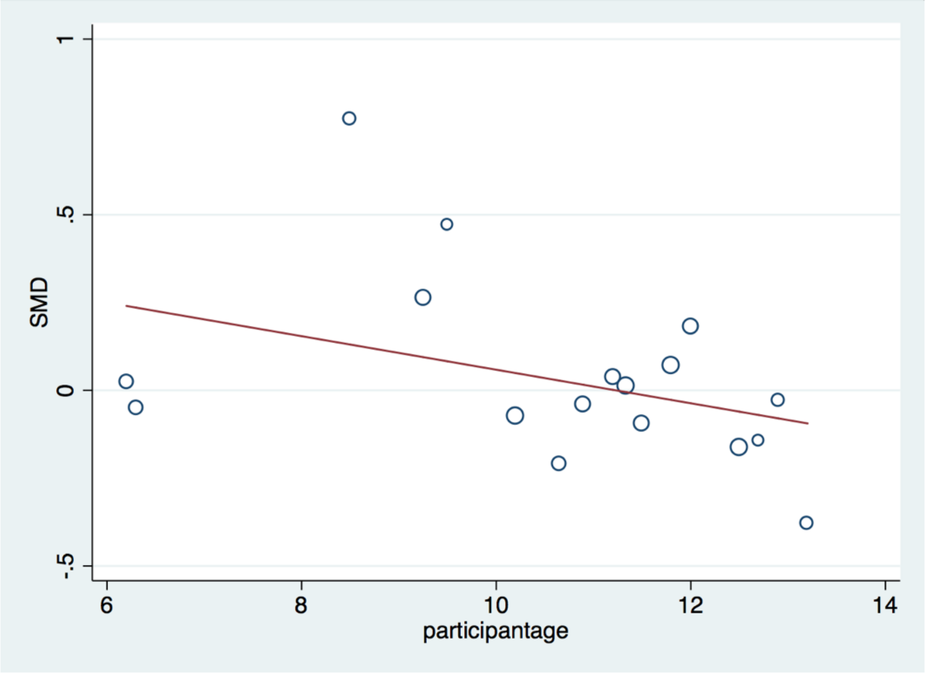

9.8 Main effect meta-regression by intervention duration (p-value: 0.975)

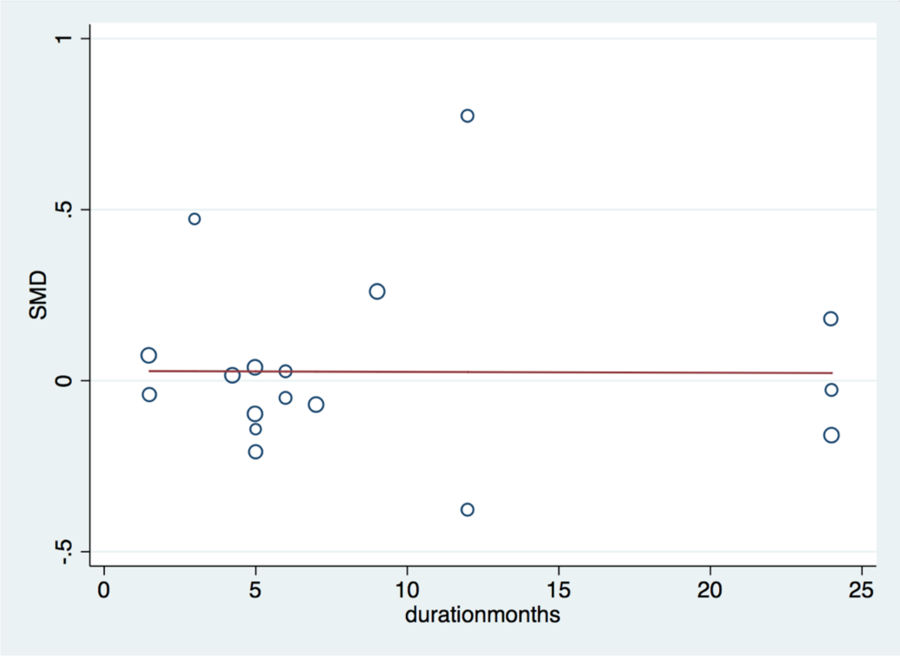

9.9 Main effect meta-analysis funnel plot

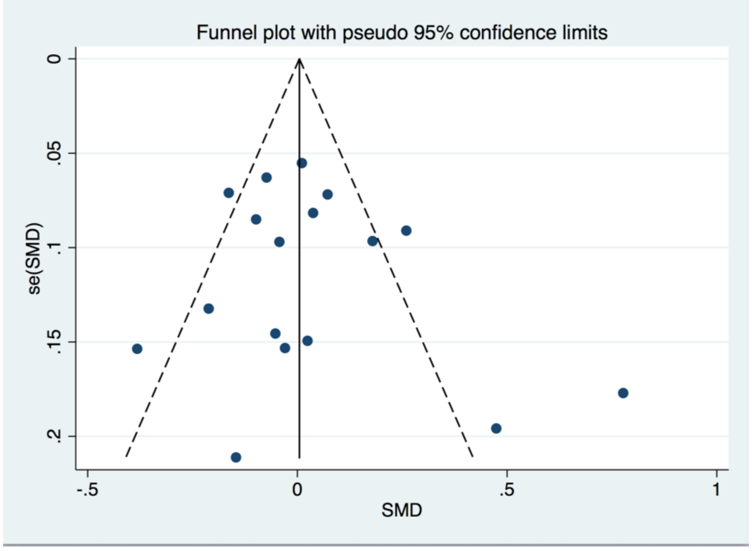

Eggers test (p-value: 0.497)

9.10 Pooled boys and girls meta-analysis and subsequent meta-regression by gender

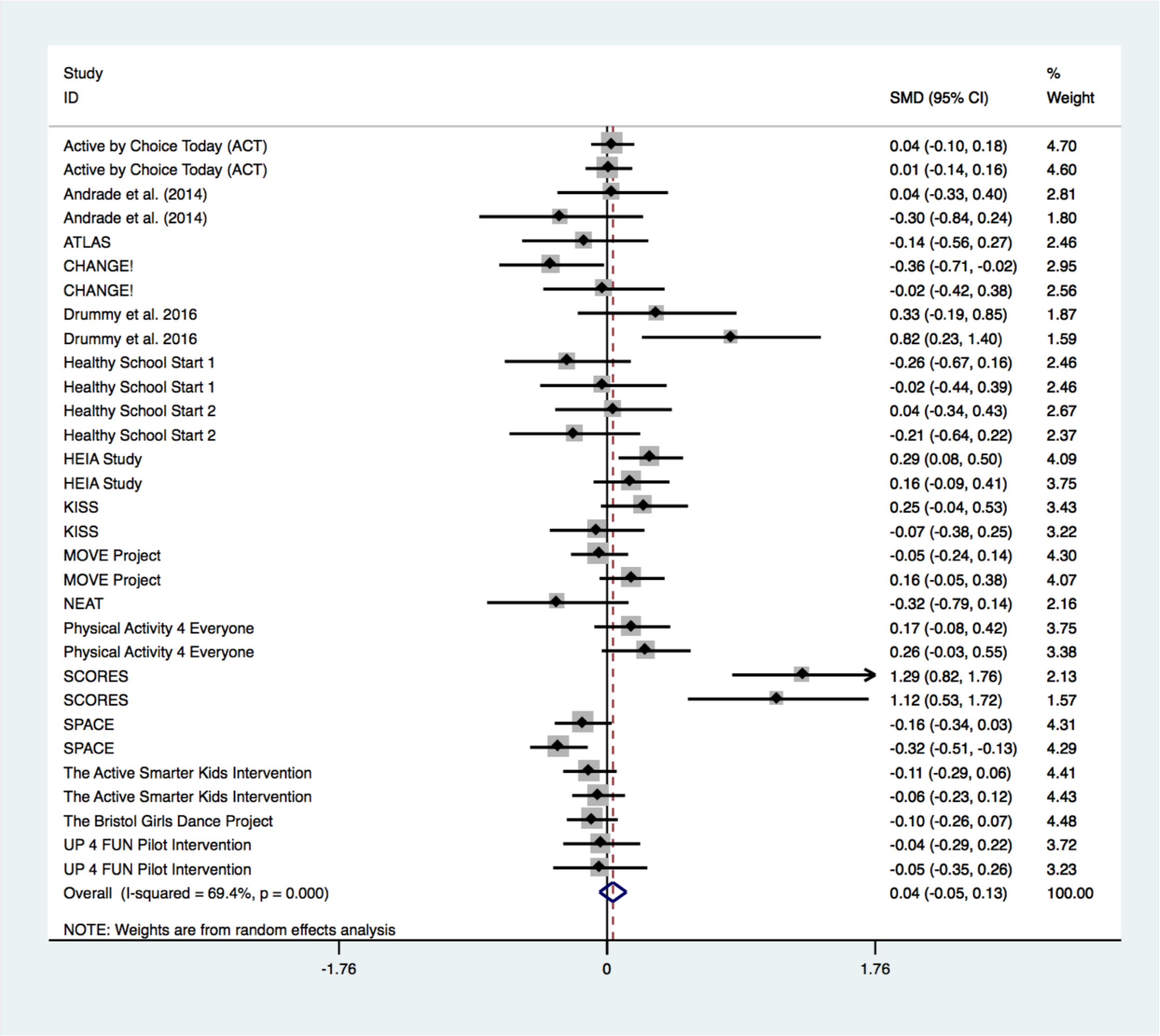

Subsequent meta-regression by gender: Coef: -0.0043184, p-value: 0.972

9.11 Pooled SEP tertiles meta-analysis and subsequent meta-regression by SEP

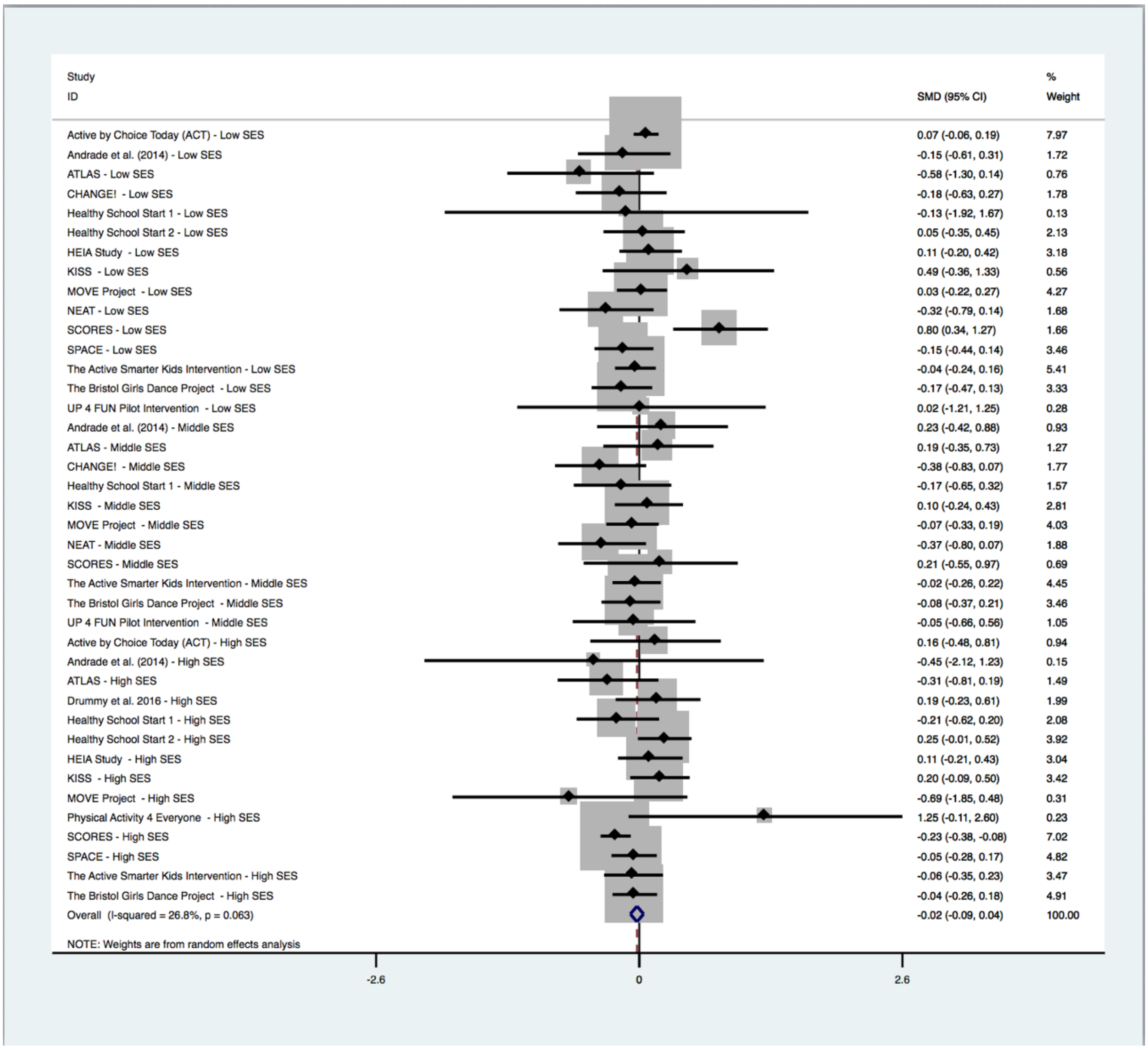

Subsequent meta-regression by SEP: Coef: -0.018218, p-value: 0.679)

9.12 Girls meta-analysis, fixed effects

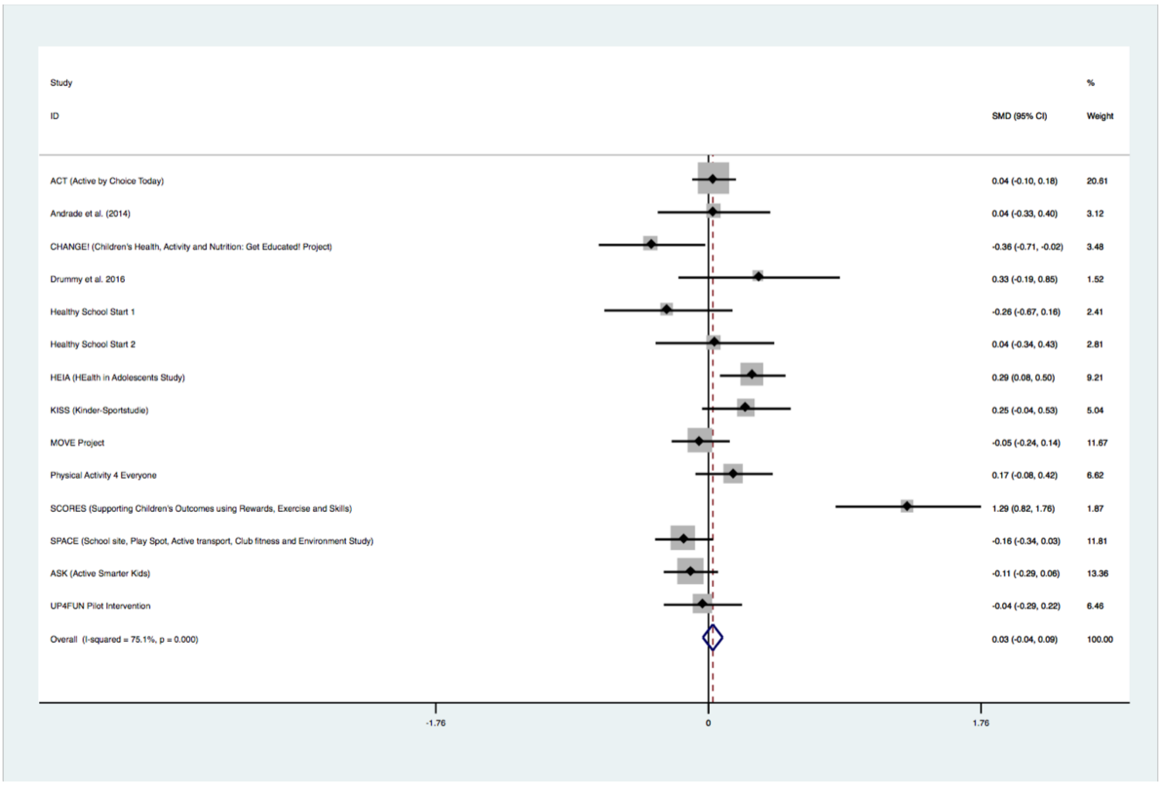

9.13 Girls meta-analysis, random effects

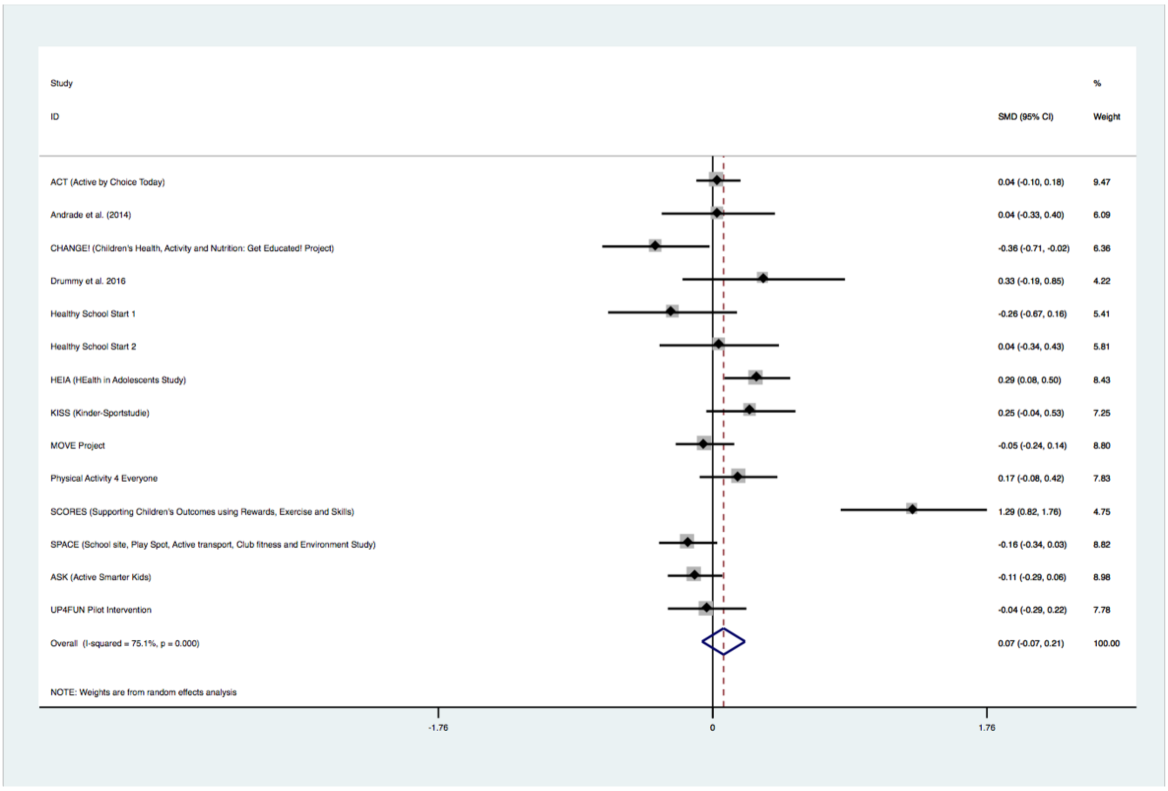

9.14 Girls effect subgroup analysis by behavioural approach

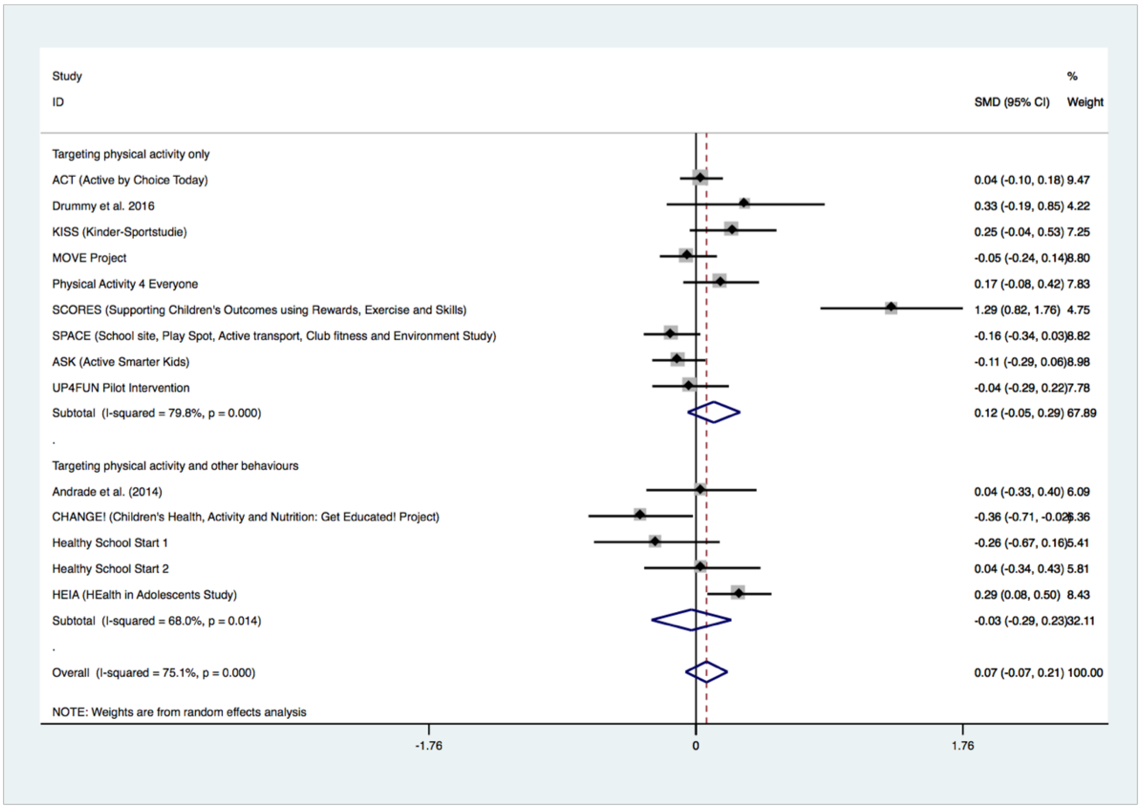

9.15 Girls effect subgroup analysis by intervention setting

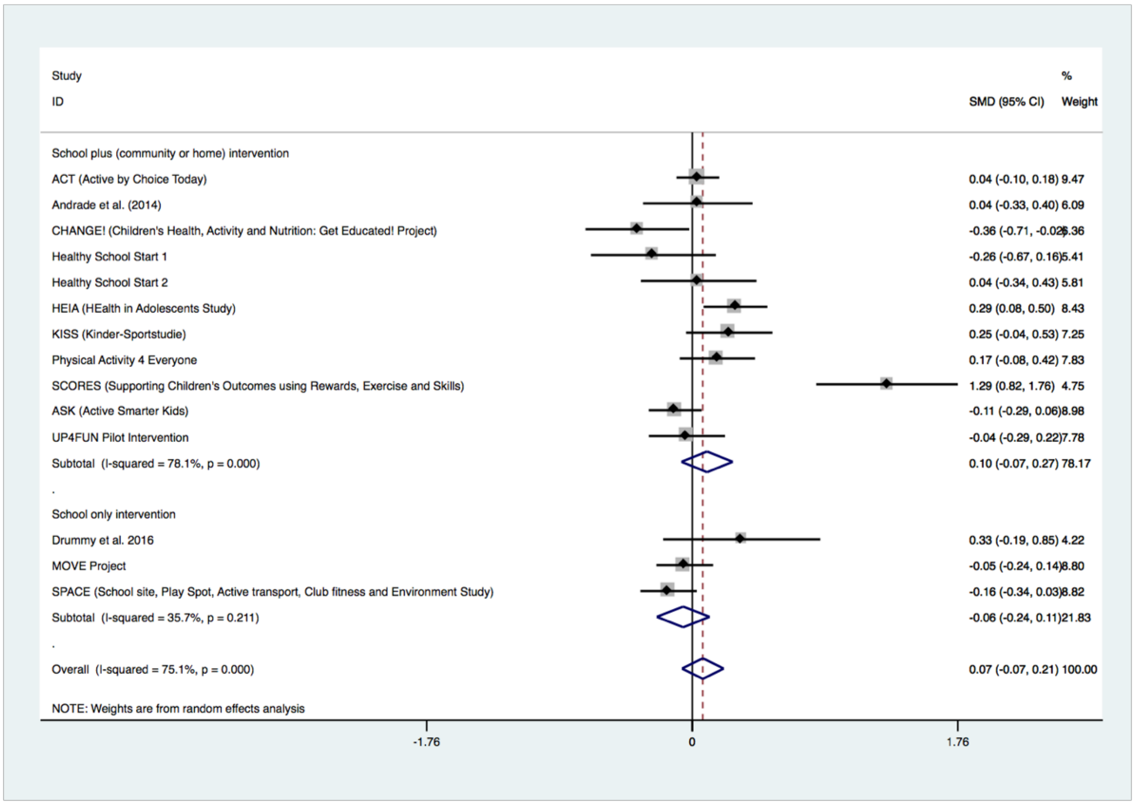

9.16 Girls effect subgroup analysis by risk of bias

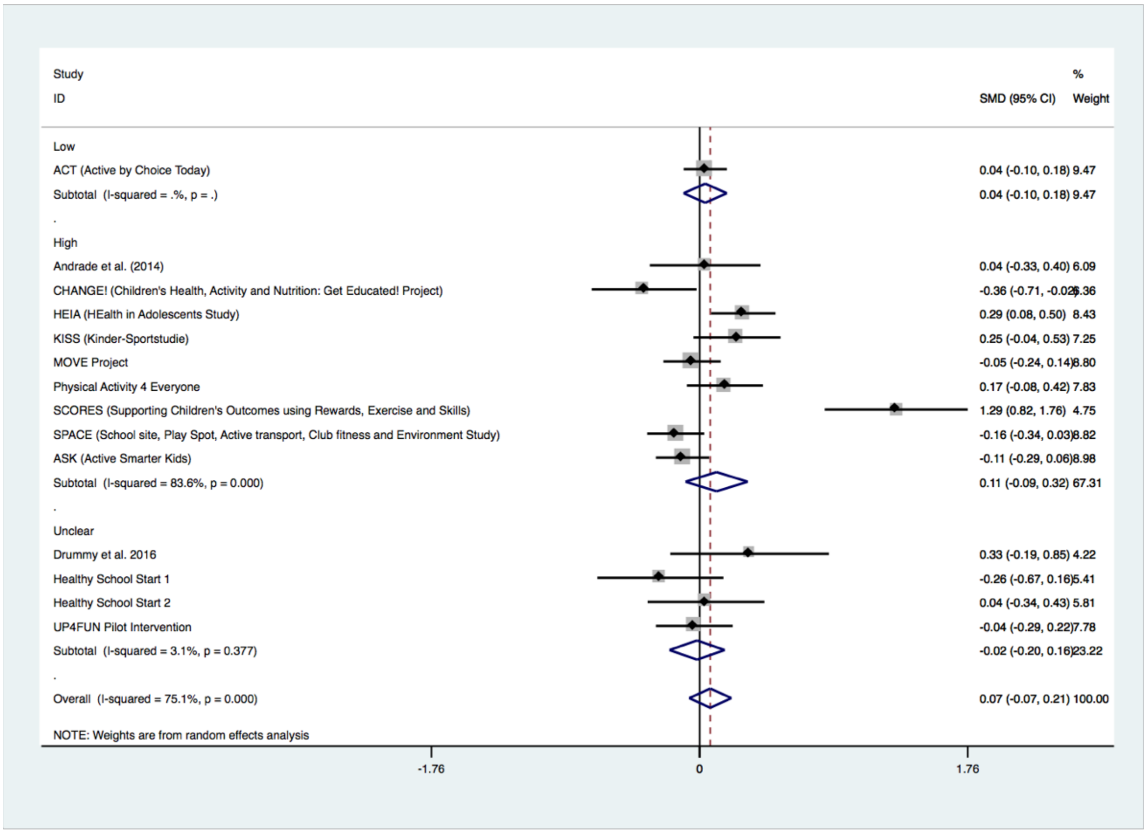

9.17 Girls effect meta-regression by sample size (p-value: 0.435)

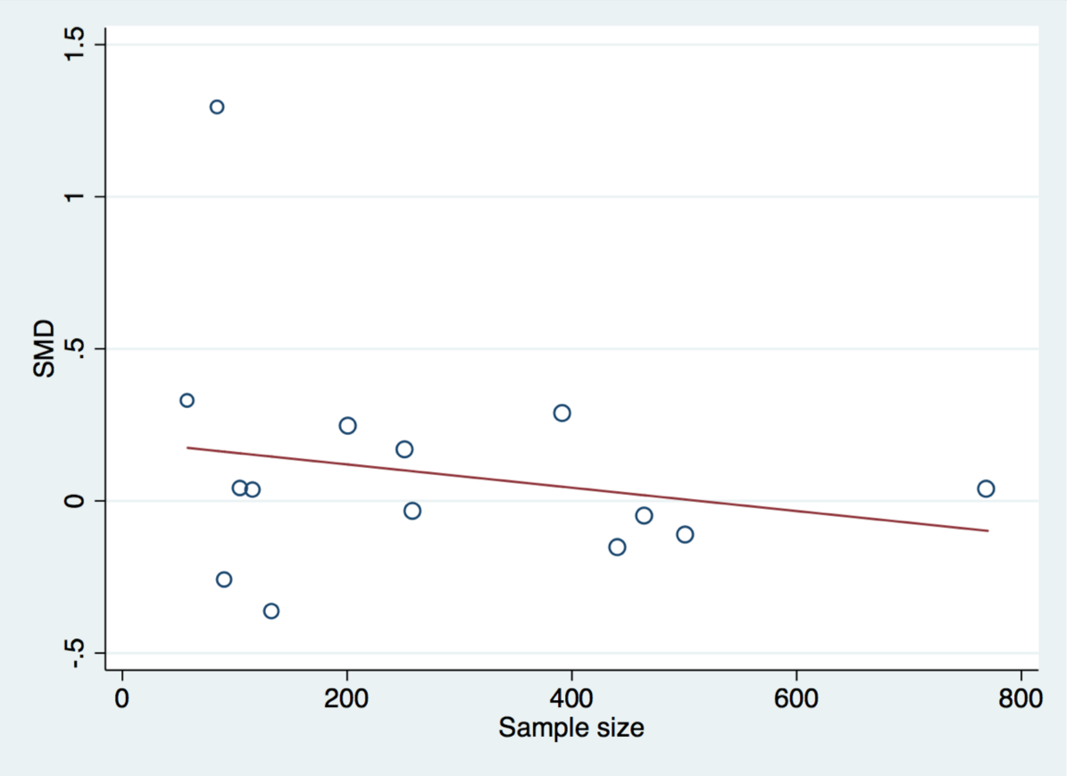

9.18 Girls effect meta-regression by participant age (p-value: 0.584)

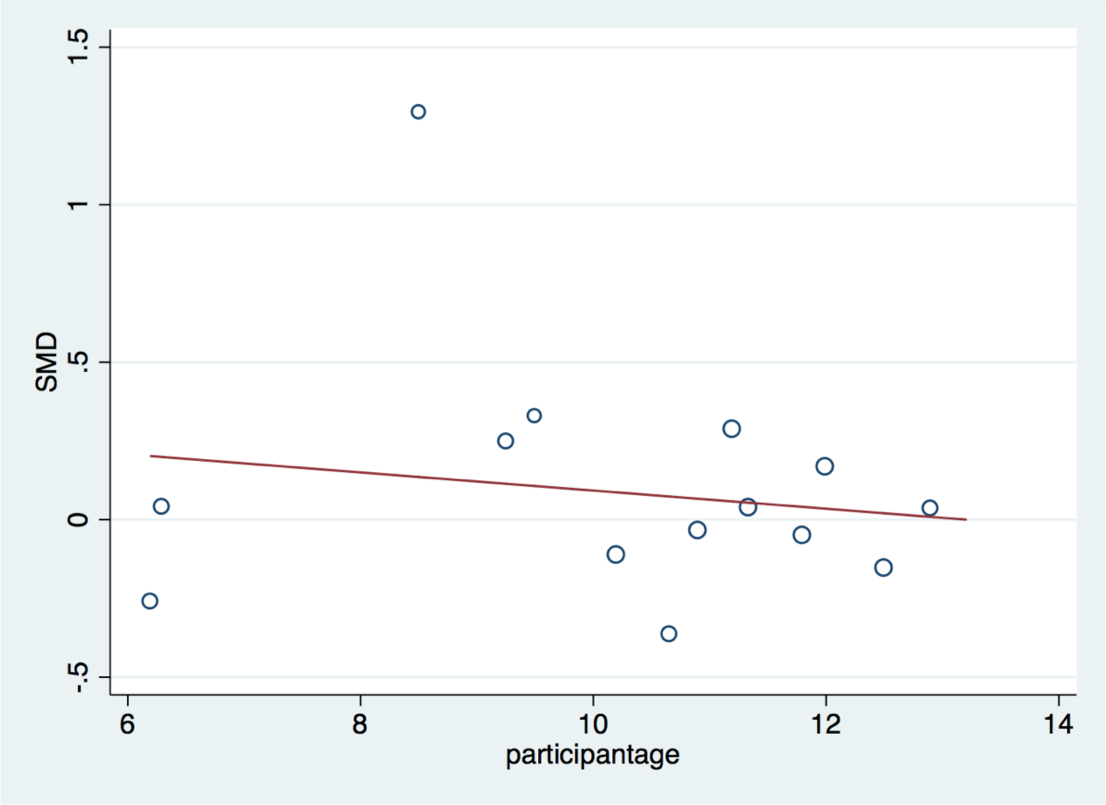

9.19 Girls effect meta-regression by intervention duration (p-value:0.804)

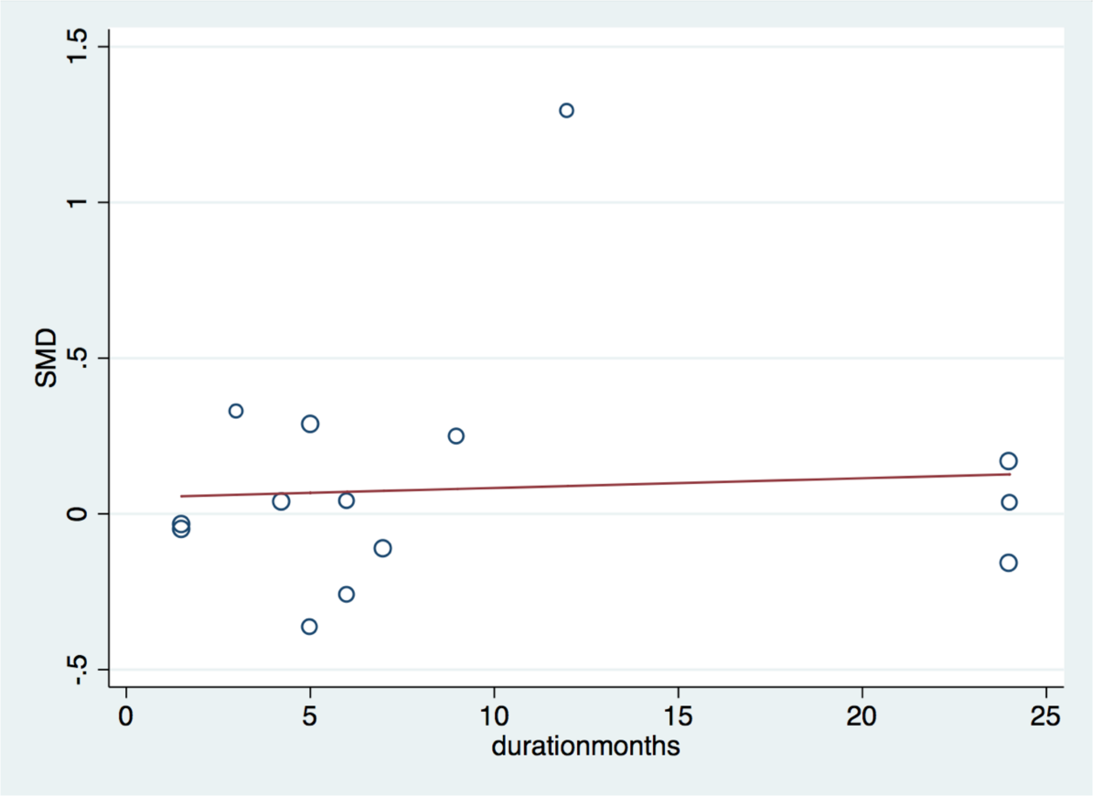

9.20 Boys meta-analysis, fixed effects

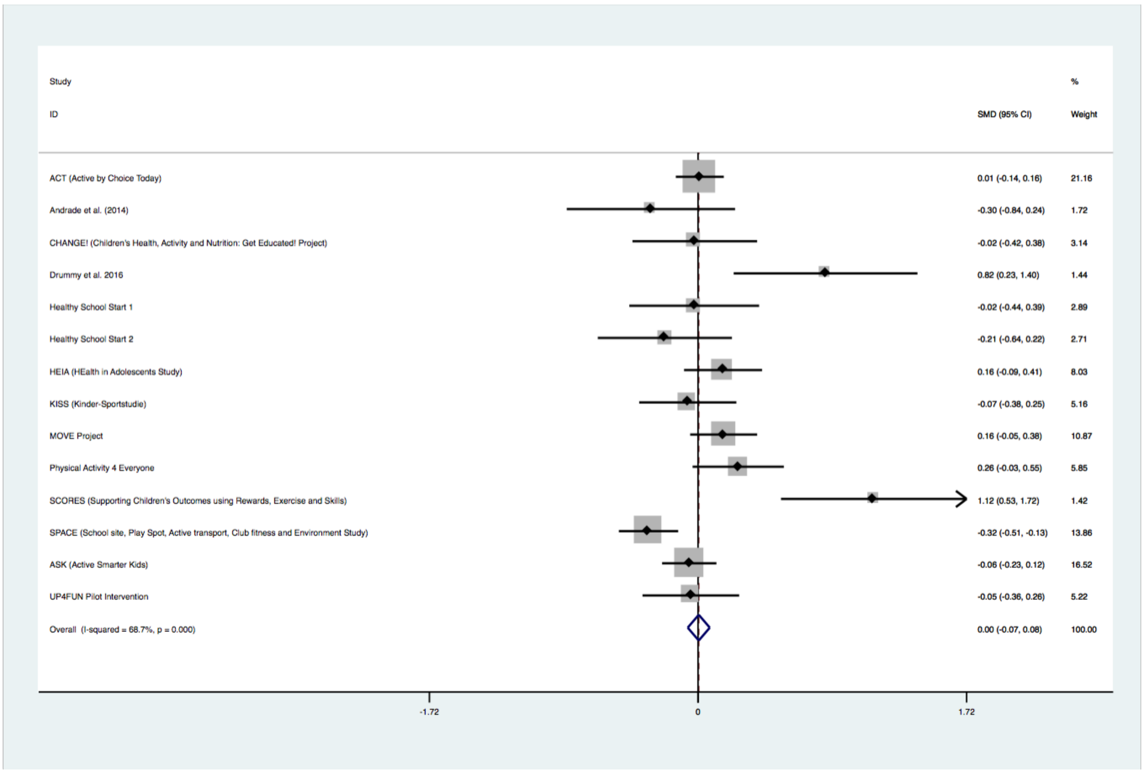

9.21 Boys meta-analysis, random effects

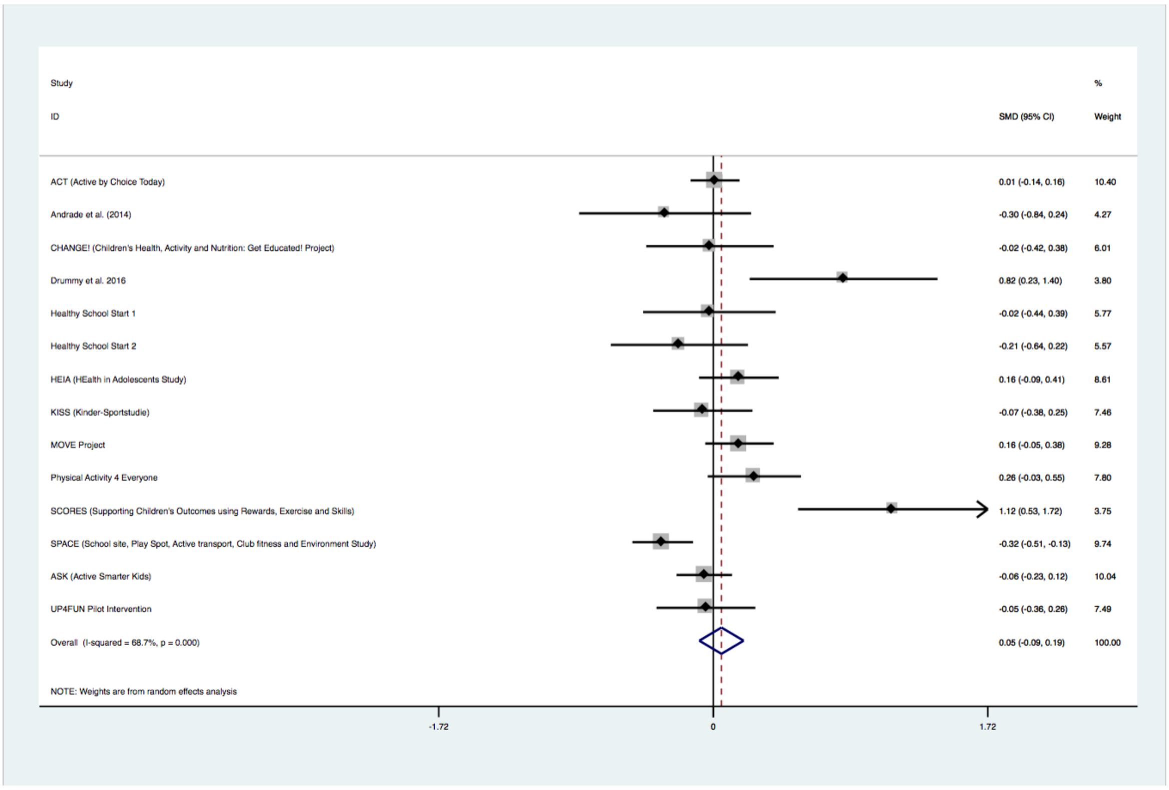

9.22 Boys effect subgroup analysis by behavioural approach

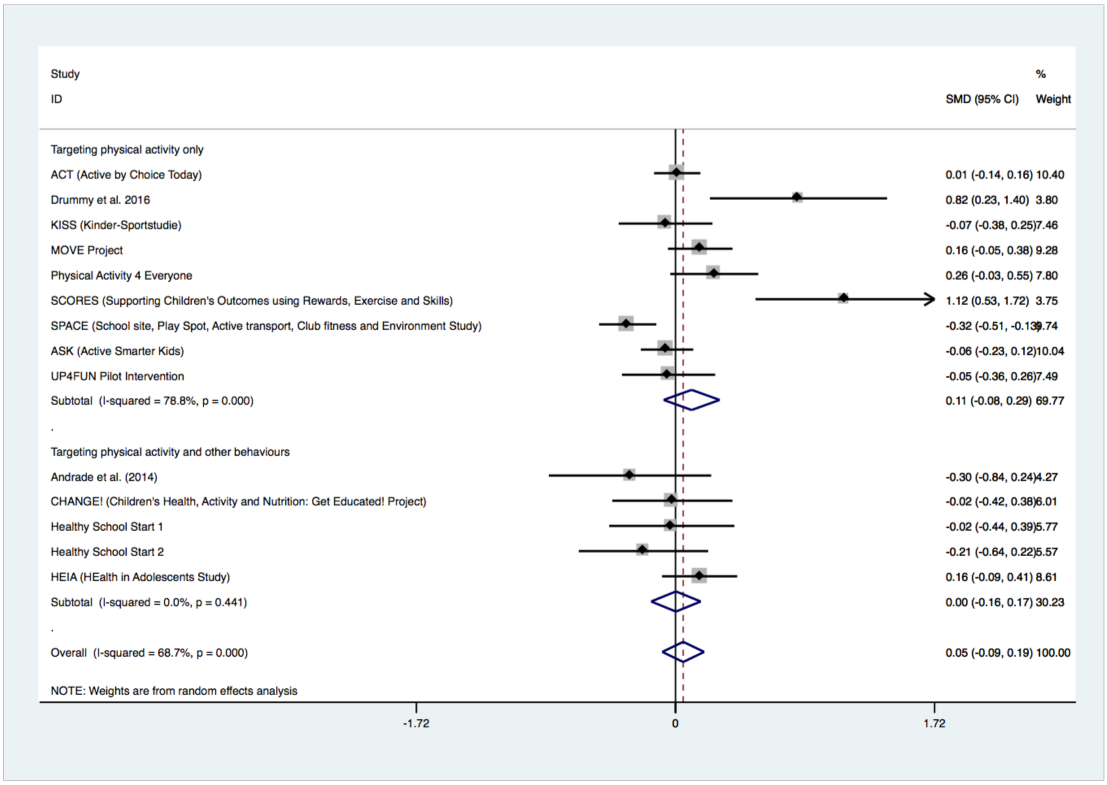

9.23 Boys effect subgroup analysis by intervention setting

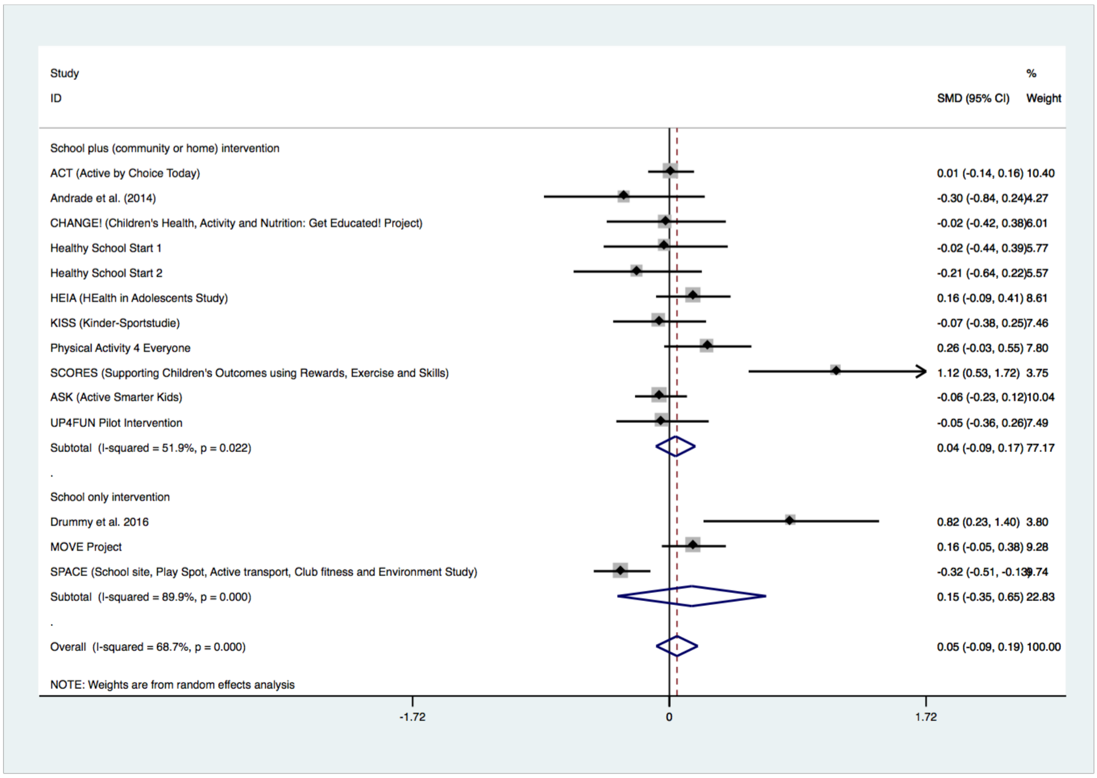

9.24 Boys effect subgroup analysis by risk of bias

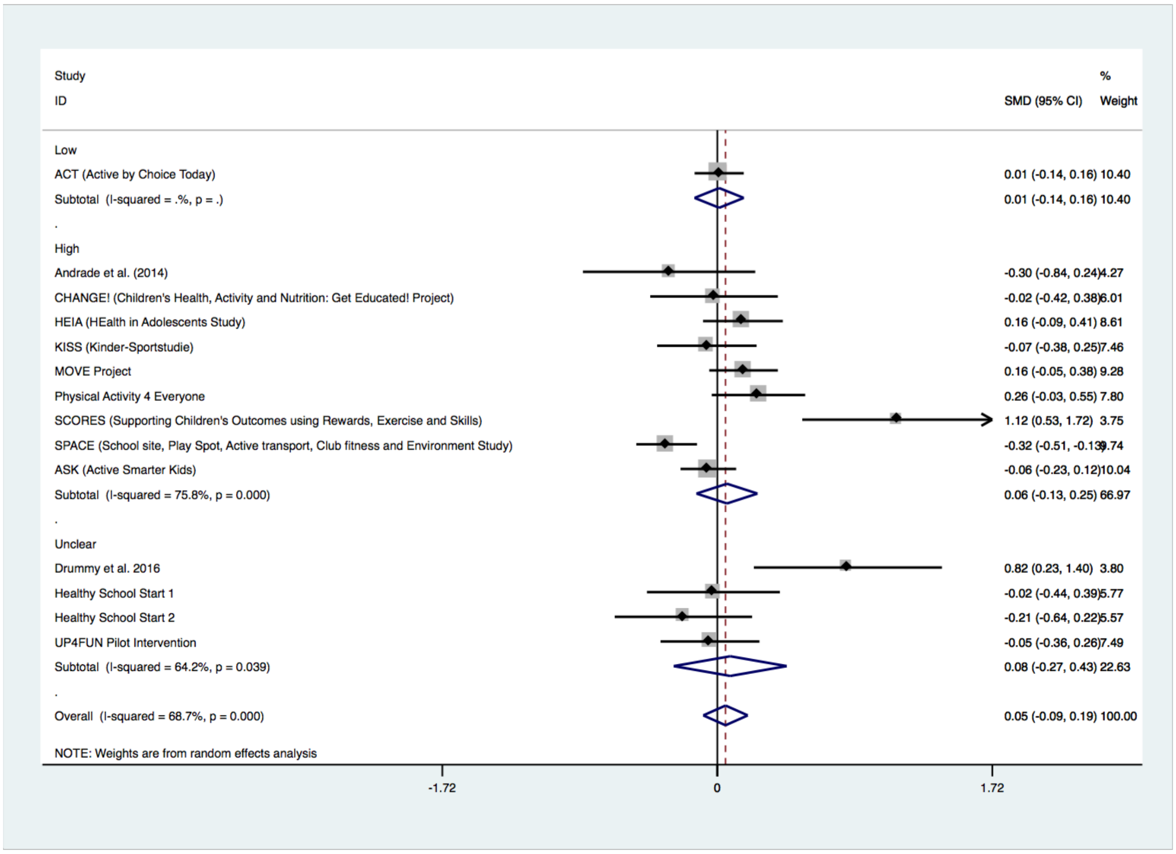

9.25 Boys effect meta-regression by sample size (p-value: 0.349)

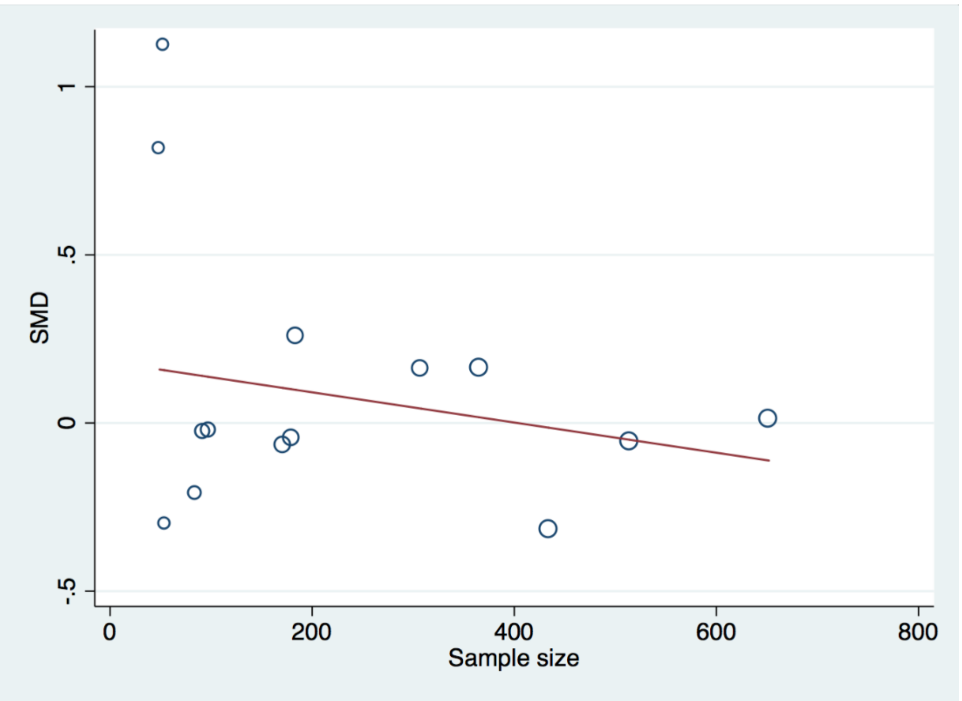

9.26 Boys effect meta-regression by participant age (p-value: 0.600)

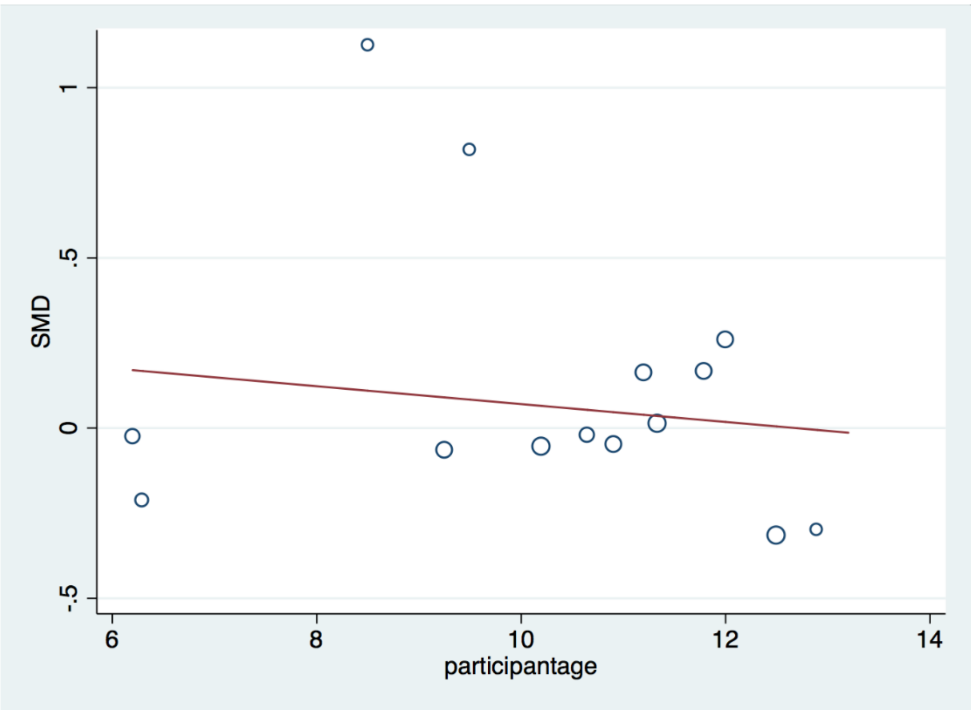

9.27 Boys effect meta-regression by intervention duration (p-value: 0.494)

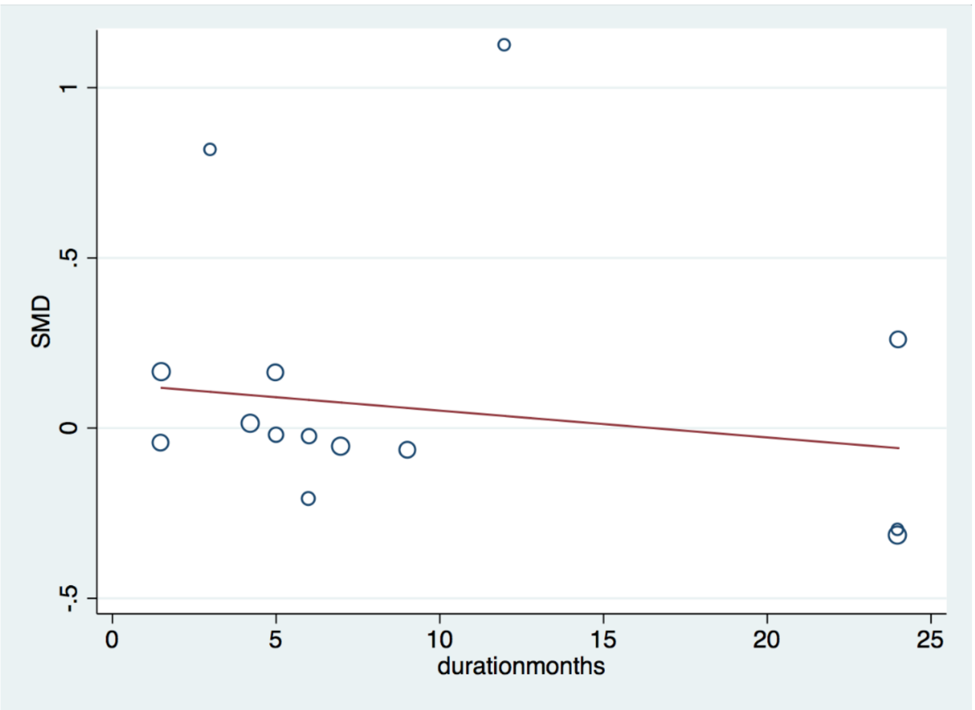

9.28 Low SEP meta-analysis, fixed effects

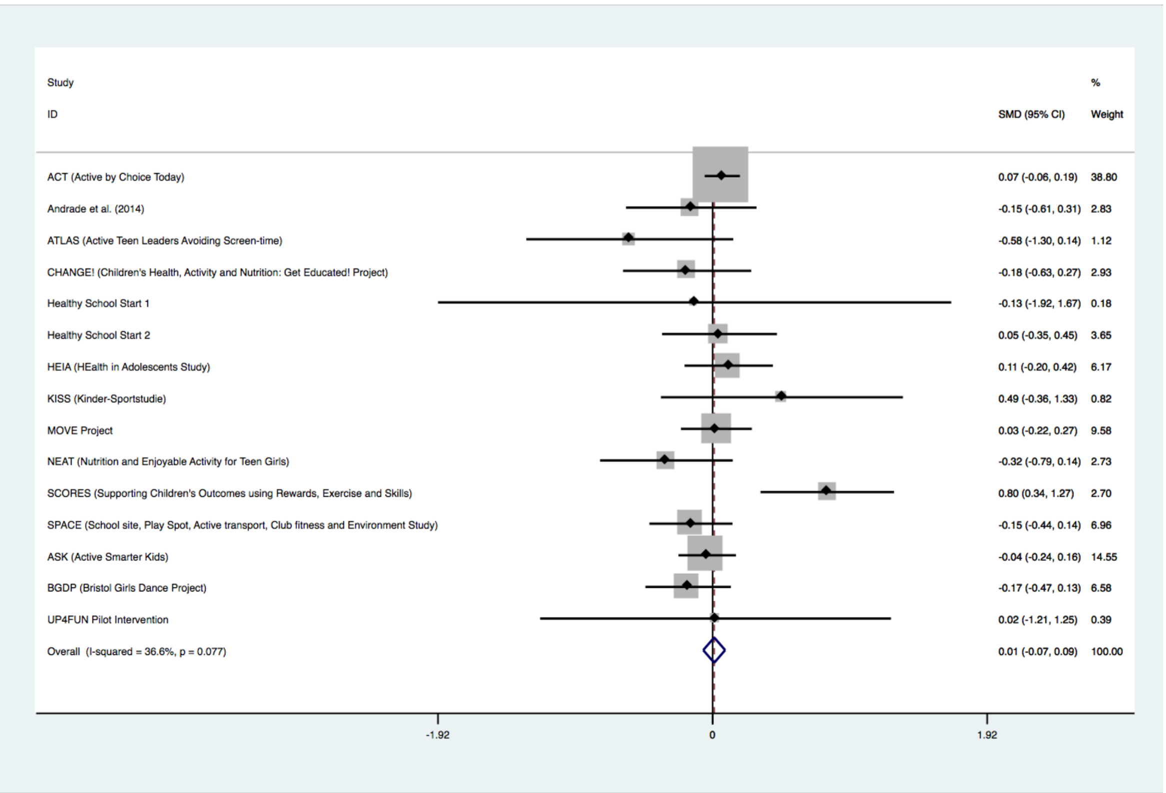

9.29 Low SEP meta-analysis, random effects

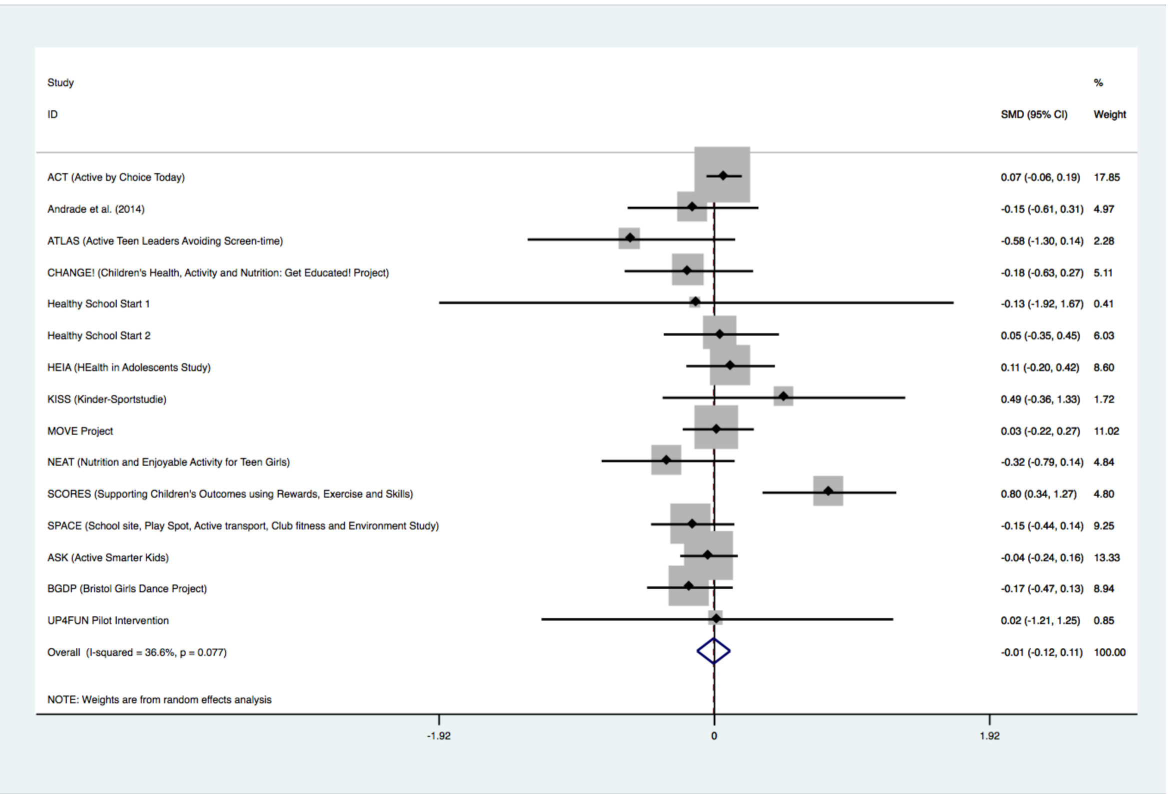

9.30 Low SEP effect subgroup analysis by behavioural approach

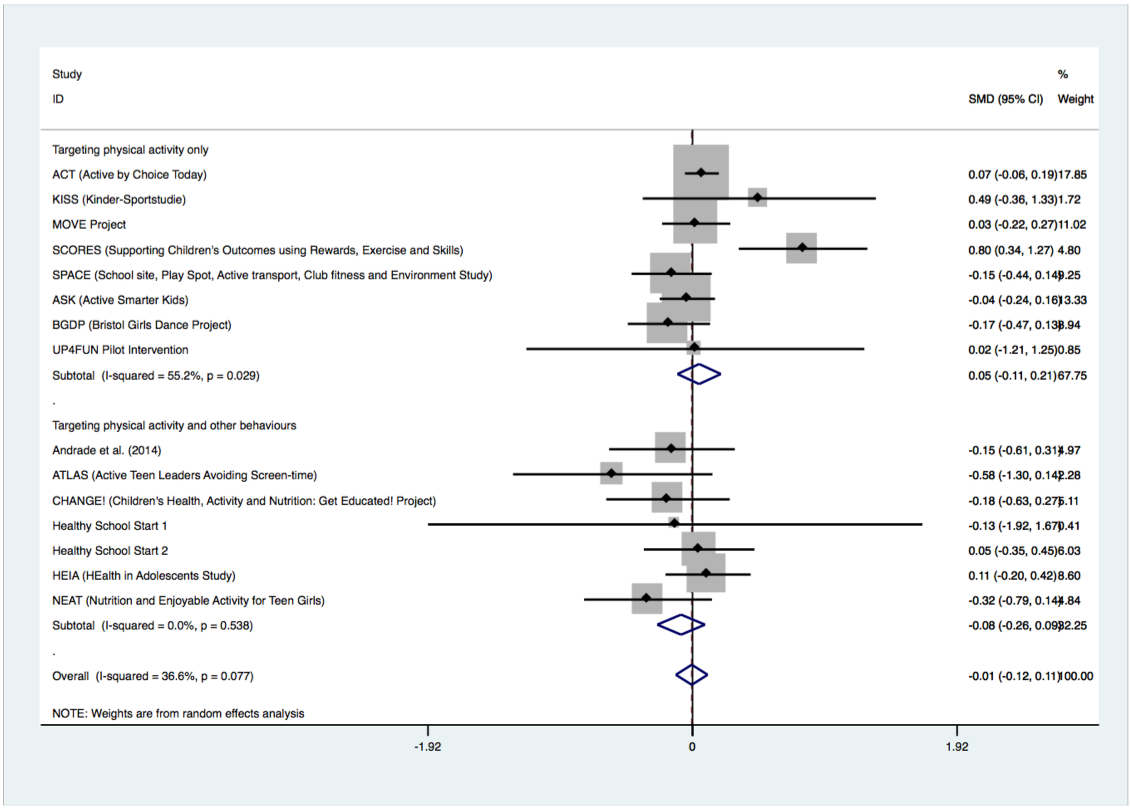

9.31 Low SEP effect subgroup analysis by intervention setting

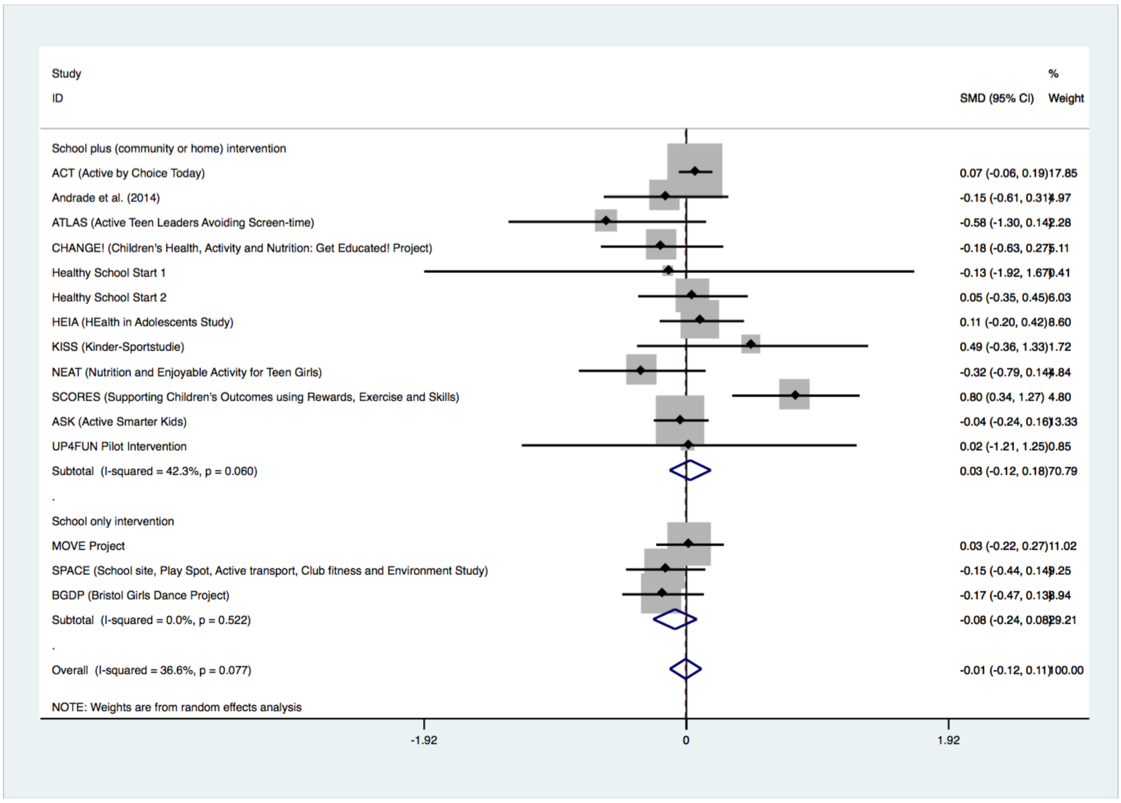

9.32 Low SEP effect subgroup analysis by risk of bias

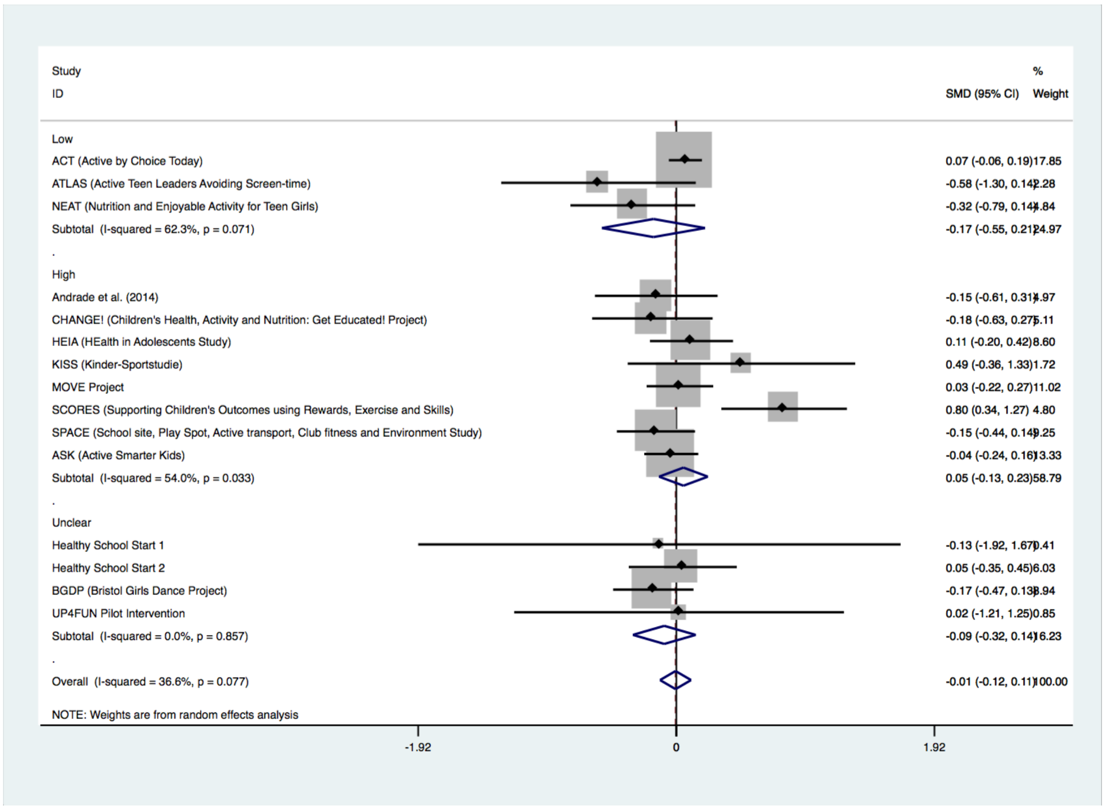

9.33 Low SEP effect meta-regression by sample size (p-value: 0.654)

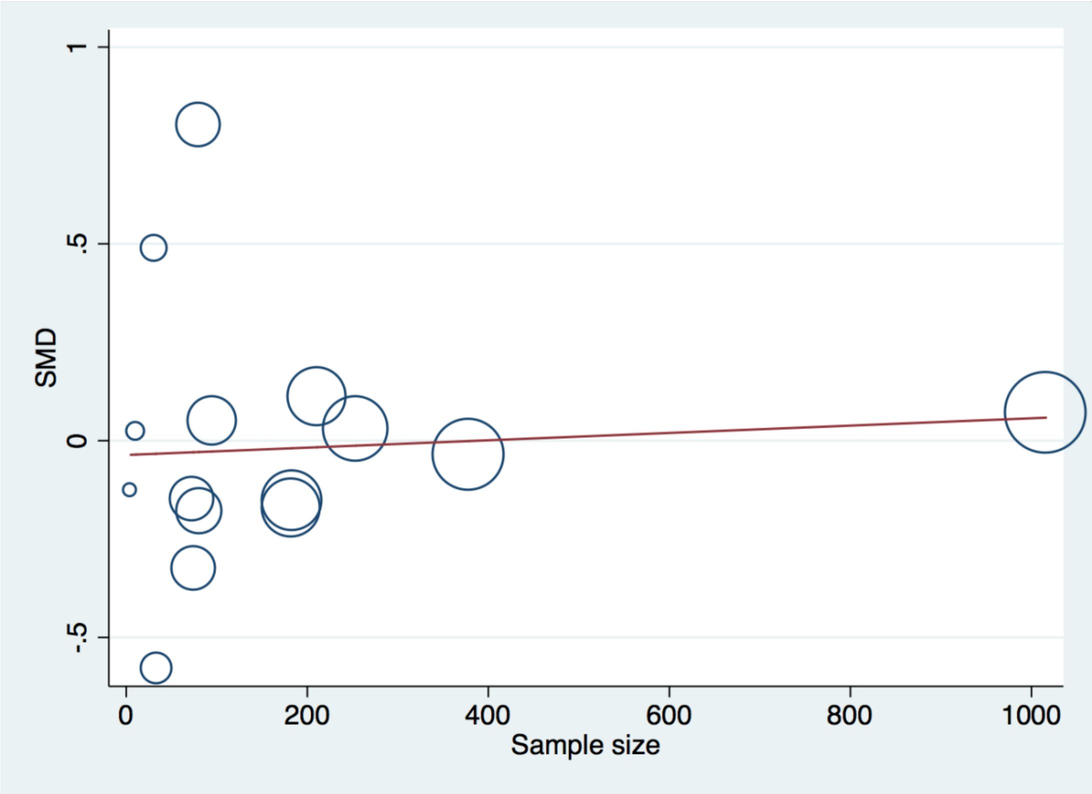

9.34 Low SEP effect meta-regression by participant age (p-value: 0.055)

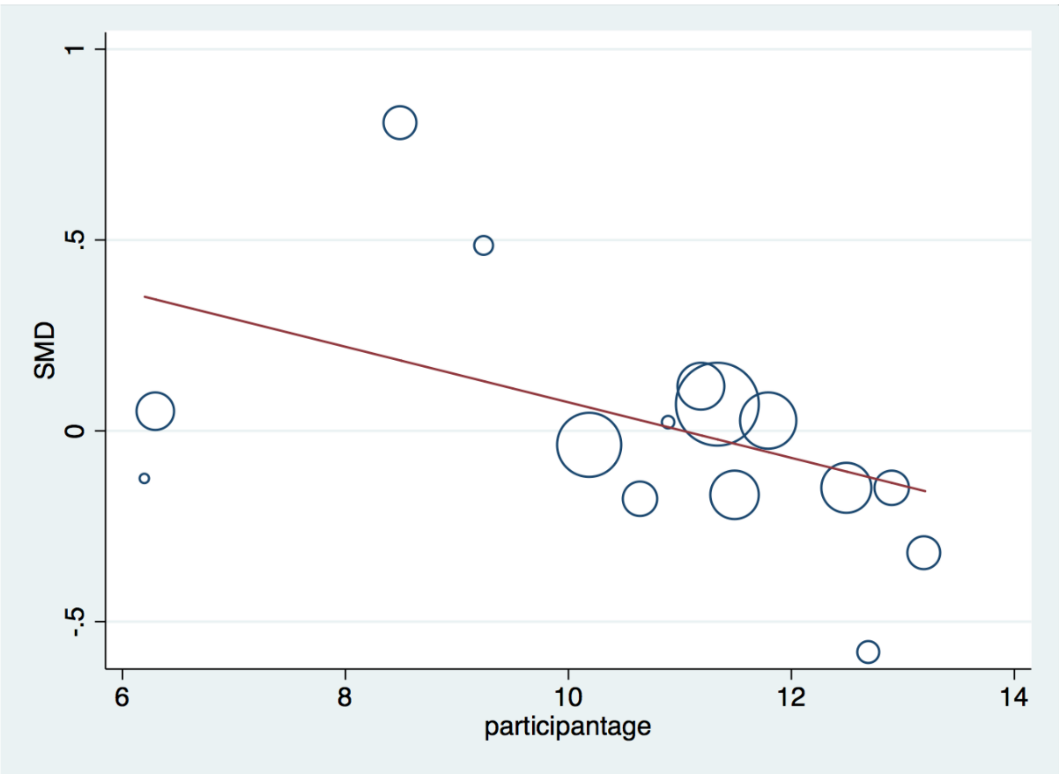

9.35 Low SEP effect meta-regression by intervention duration (p-value: 0.517)

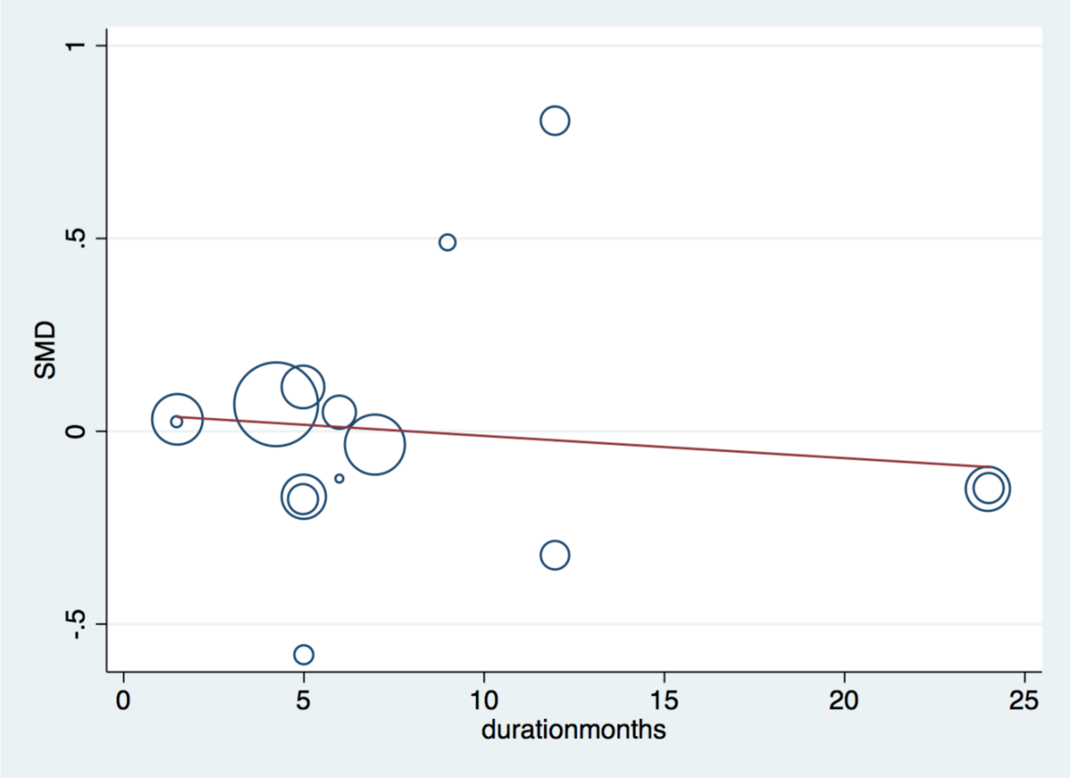

9.36 Middle SEP meta-analysis, fixed effects

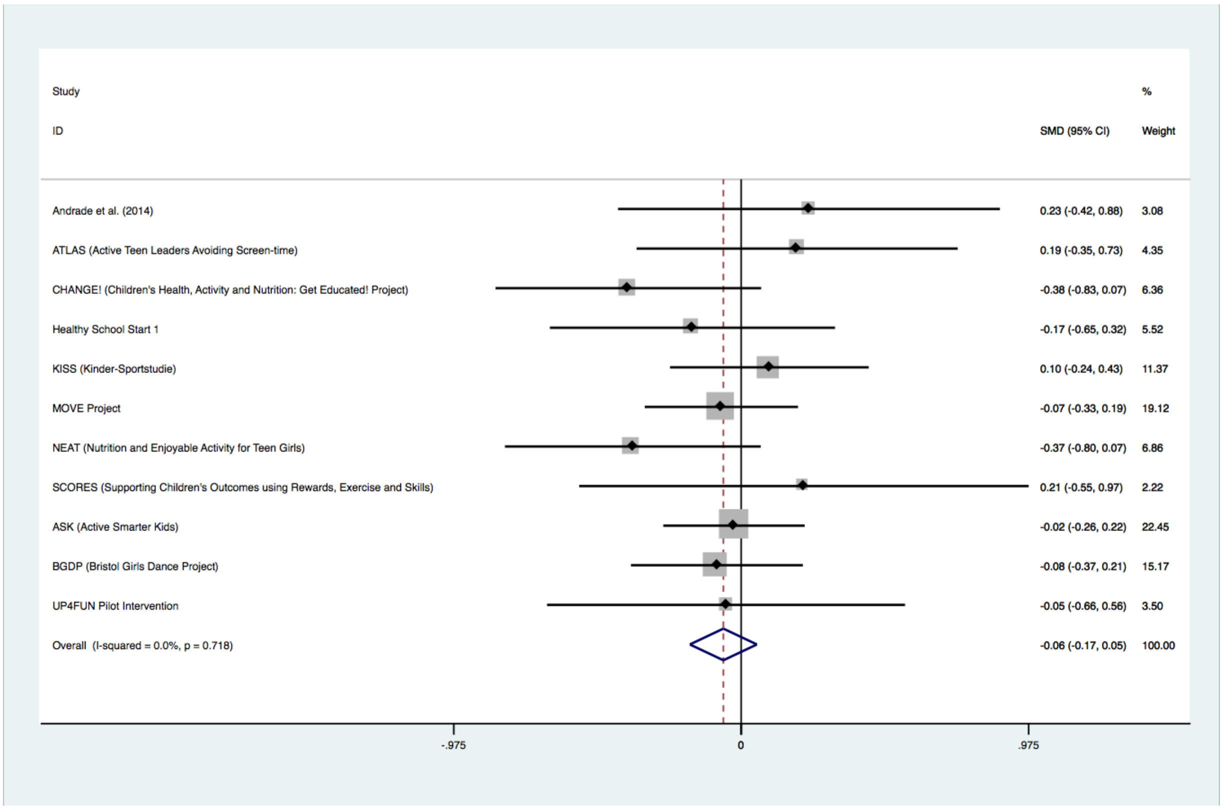

9.37 Middle SEP meta-analysis, random effects

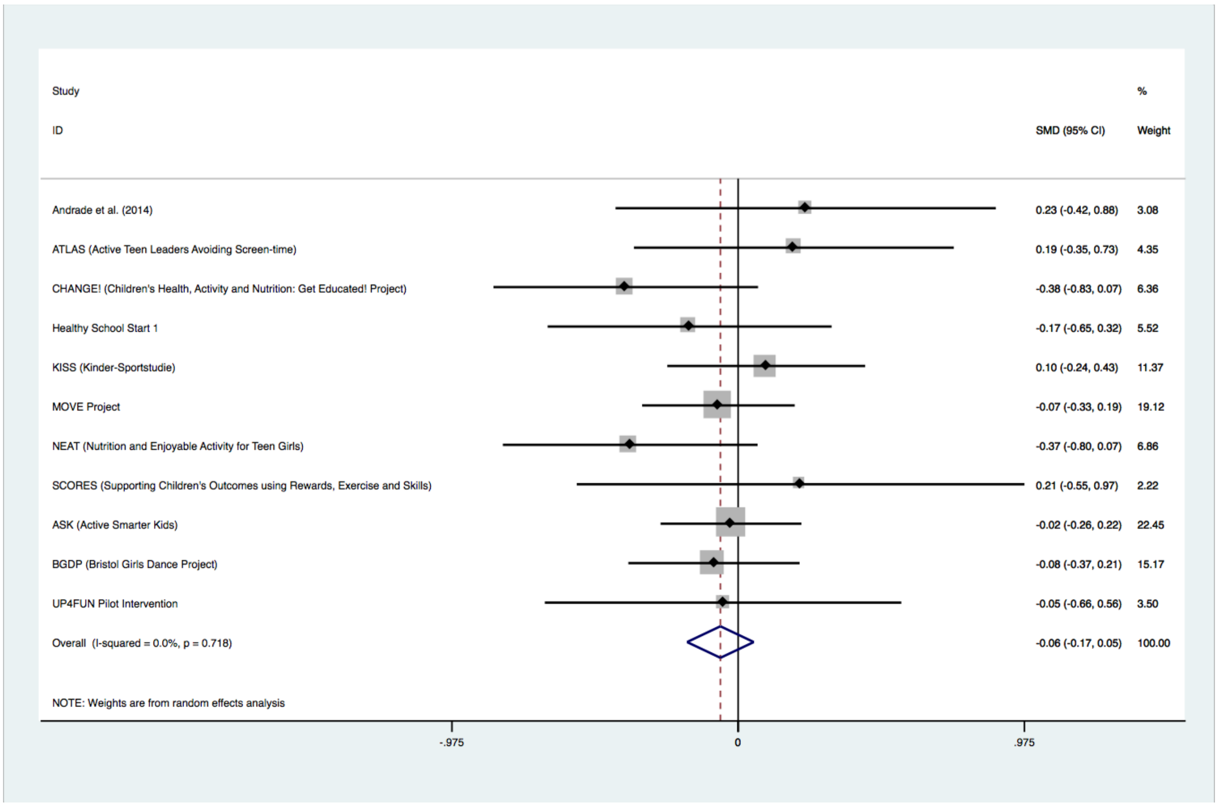

9.38 Middle SEP effect subgroup analysis by behavioural approach

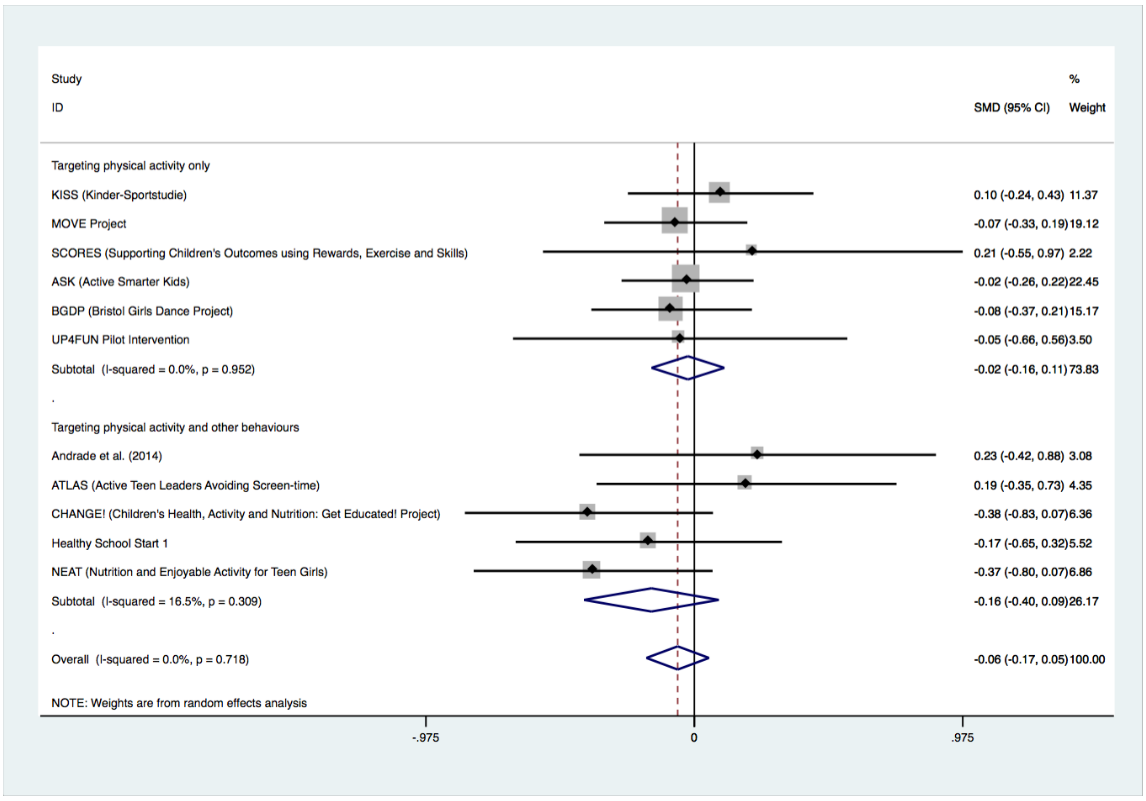

9.39 Middle SEP effect subgroup analysis by intervention setting

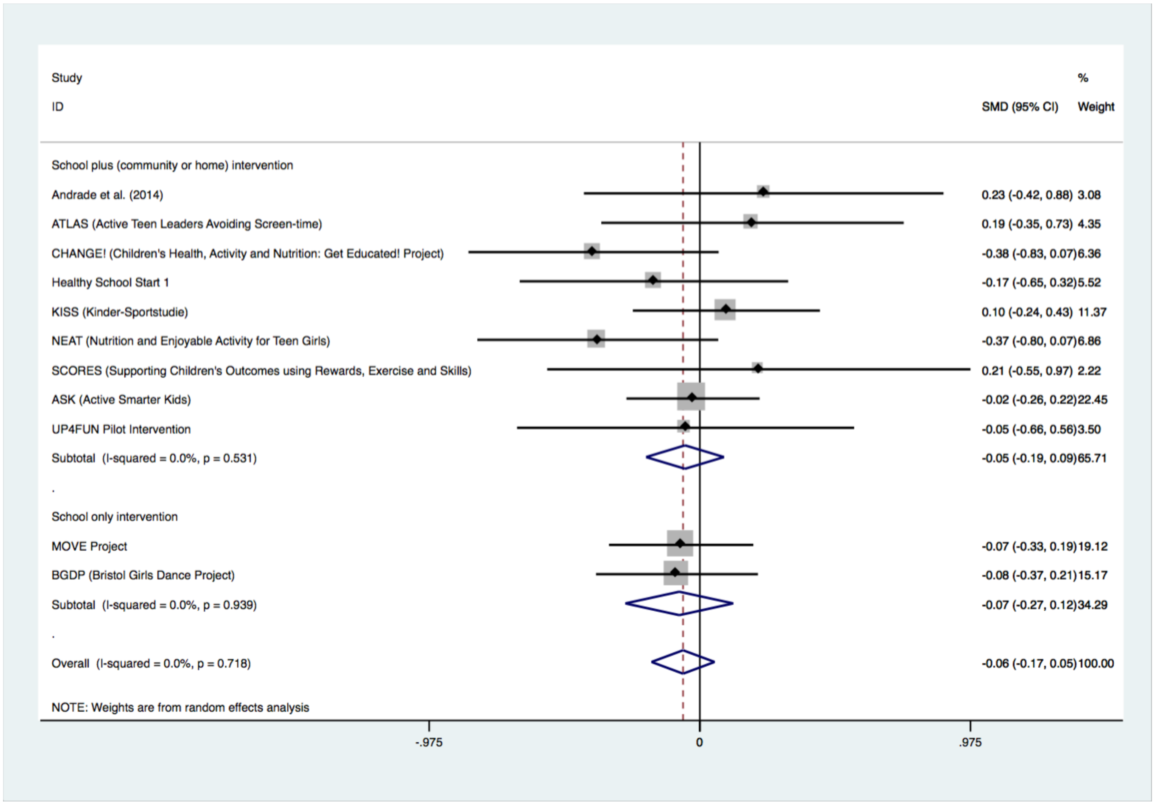

9.40 Middle SEP effect subgroup analysis by risk of bias

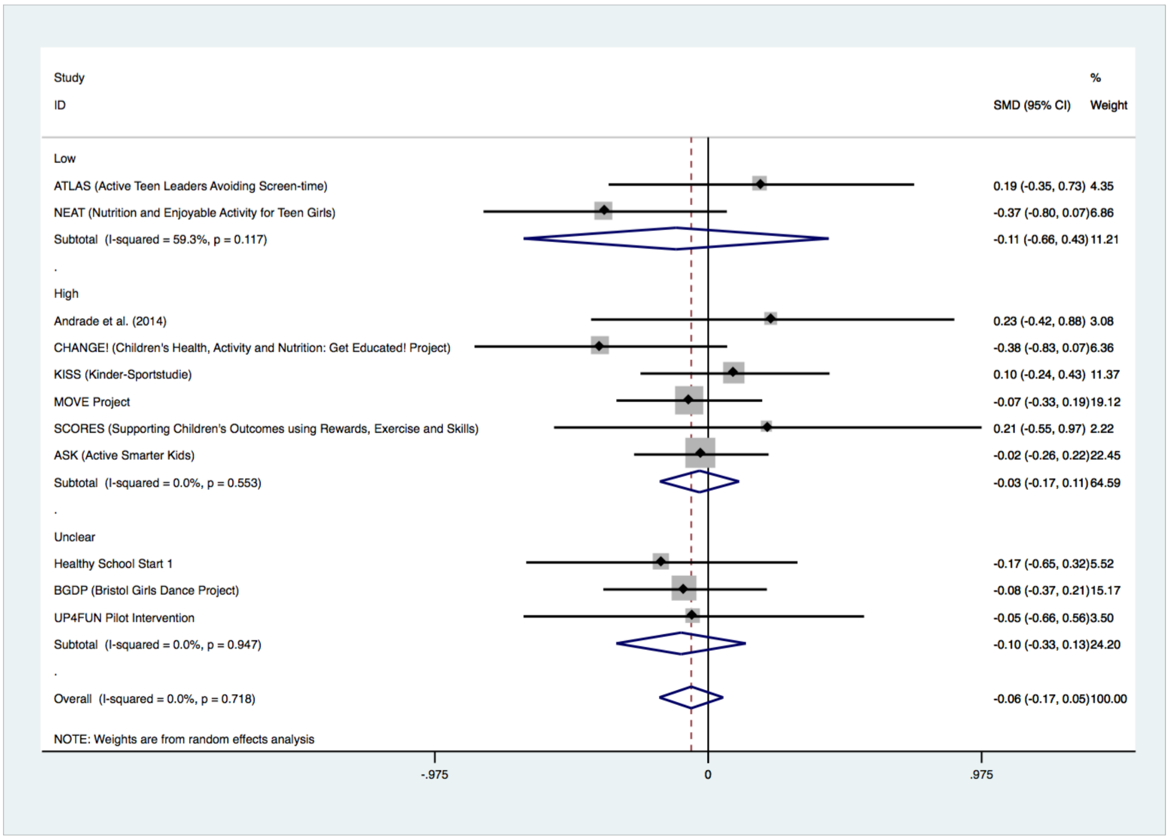

9.41 Middle SEP effect meta-regression by sample size (p-value: 0.830)

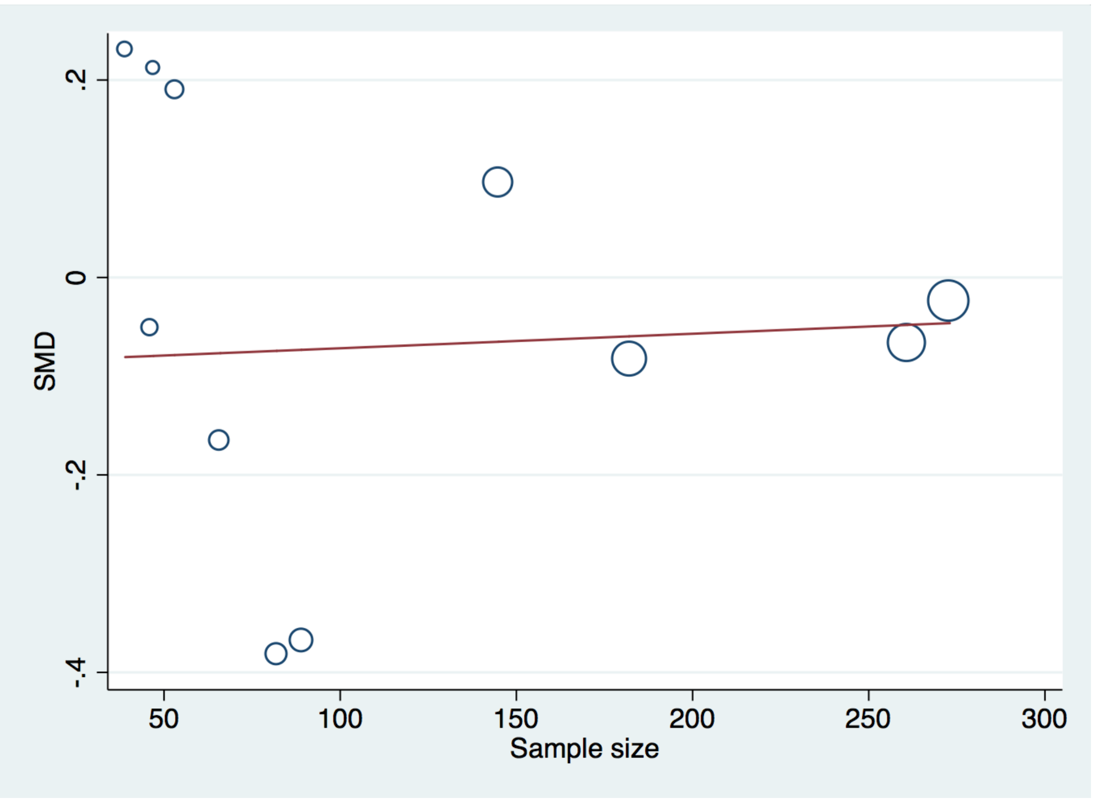

9.42 Middle SEP effect meta-regression by participant age (p-value: 0.745)

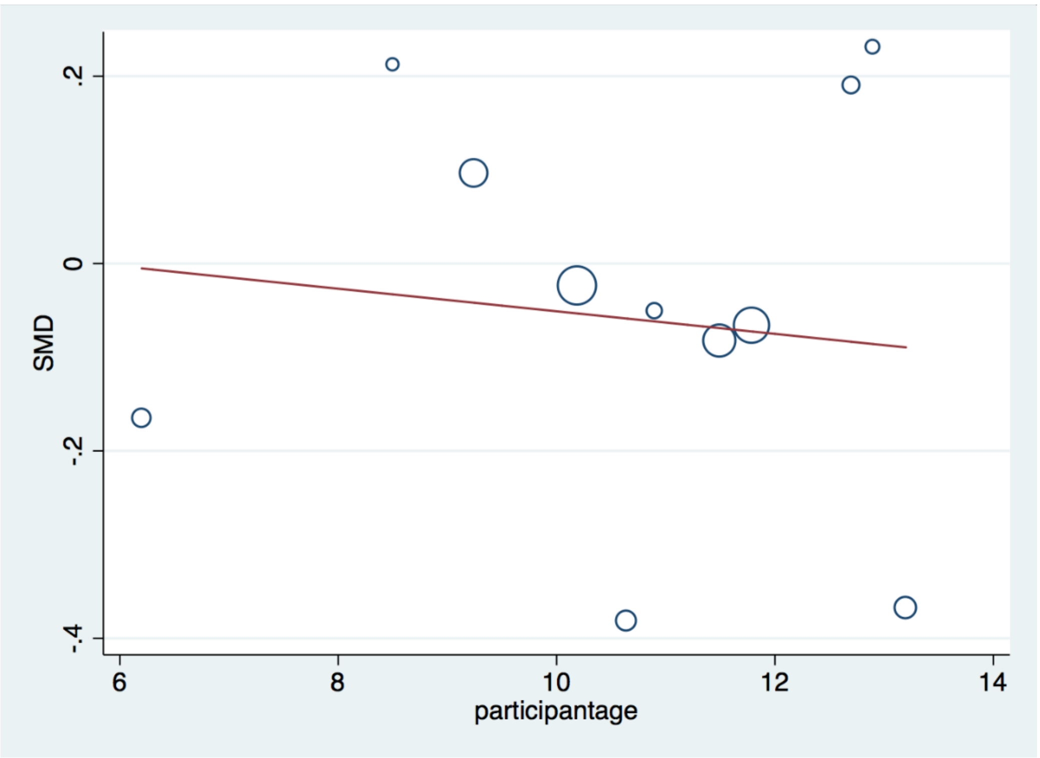

9.43 Middle SEP effect meta-regression by intervention duration (p-value: 0.570)

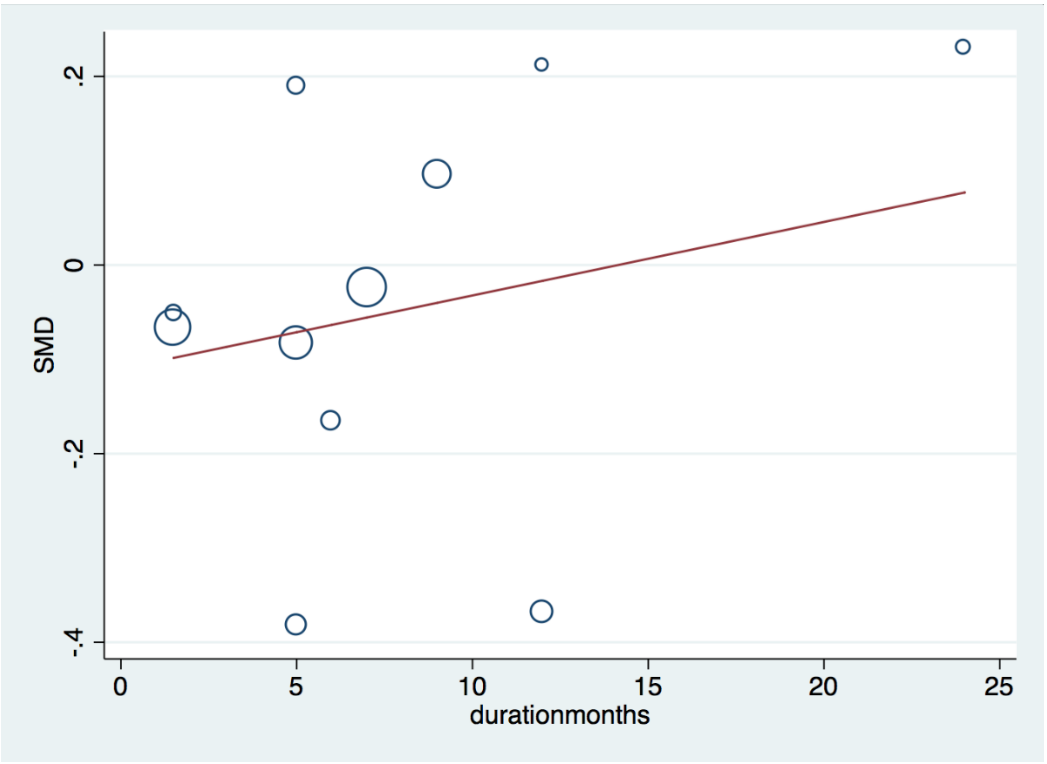

9.44 High SEP meta-analysis, fixed effects

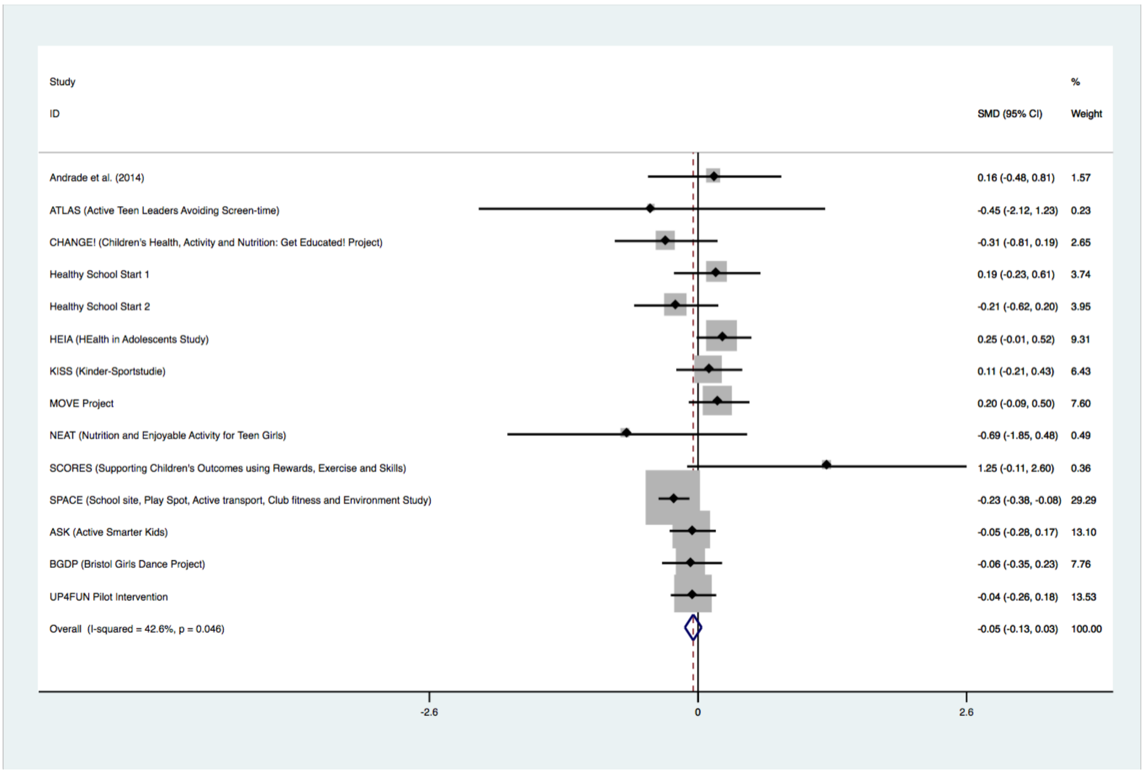

9.45 High SEP meta-analysis, random effects

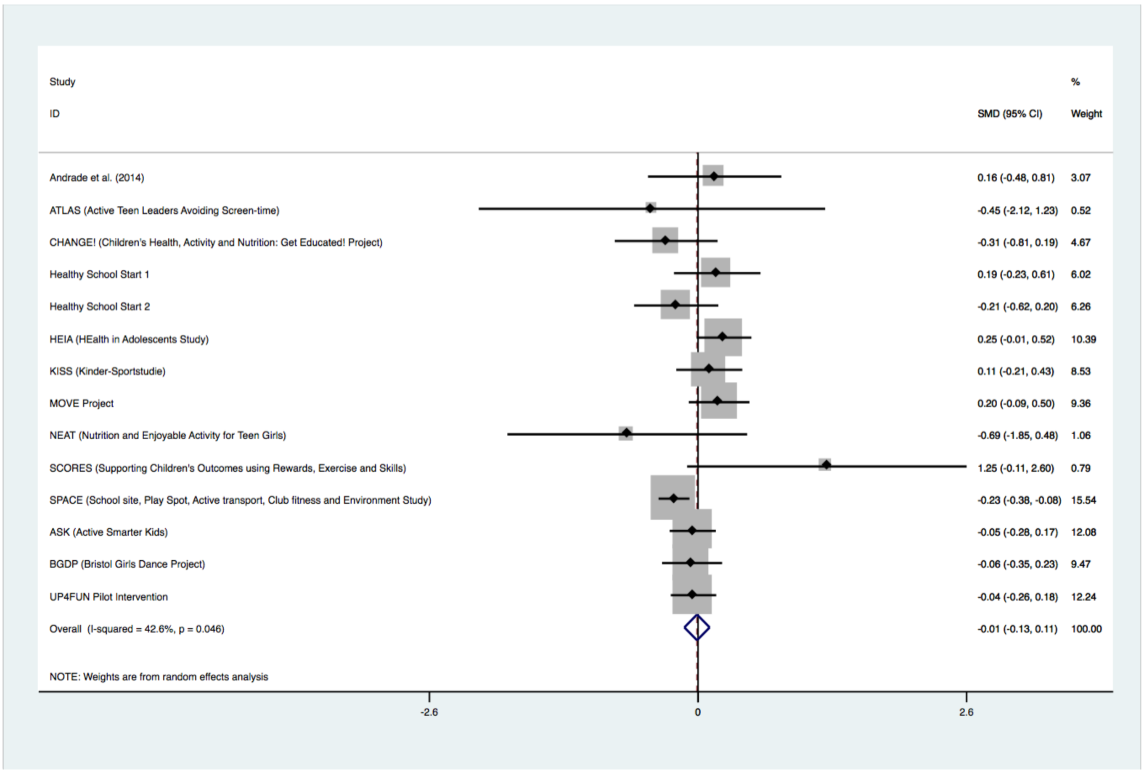

9.46 High SEP effect subgroup analysis by behavioural approach

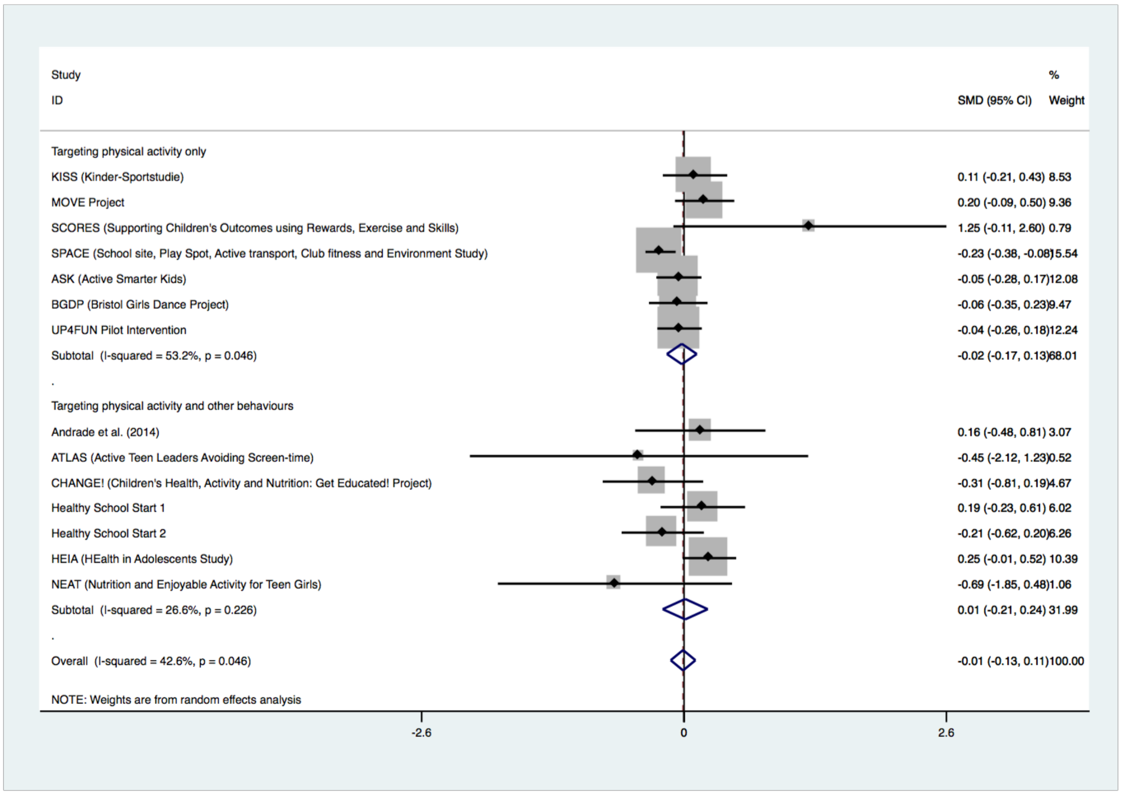

9.47 High SEP effect subgroup analysis by intervention setting

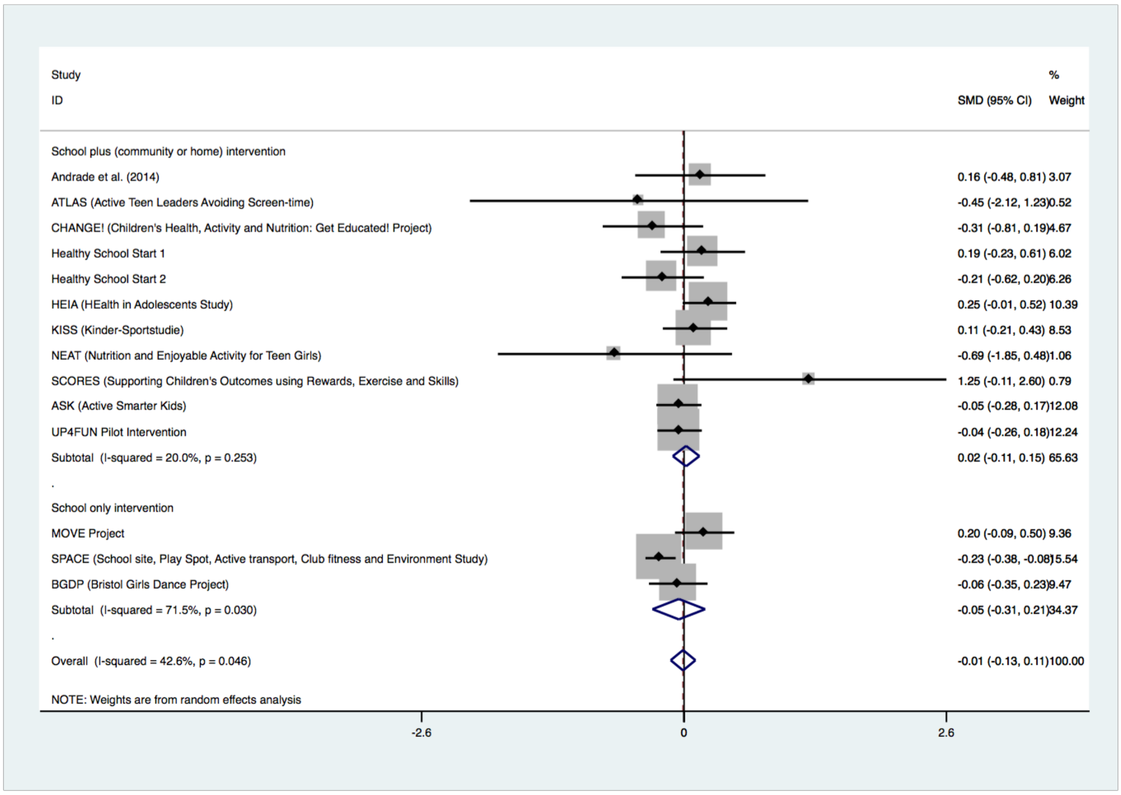

9.48 High SEP effect subgroup analysis by risk of bias

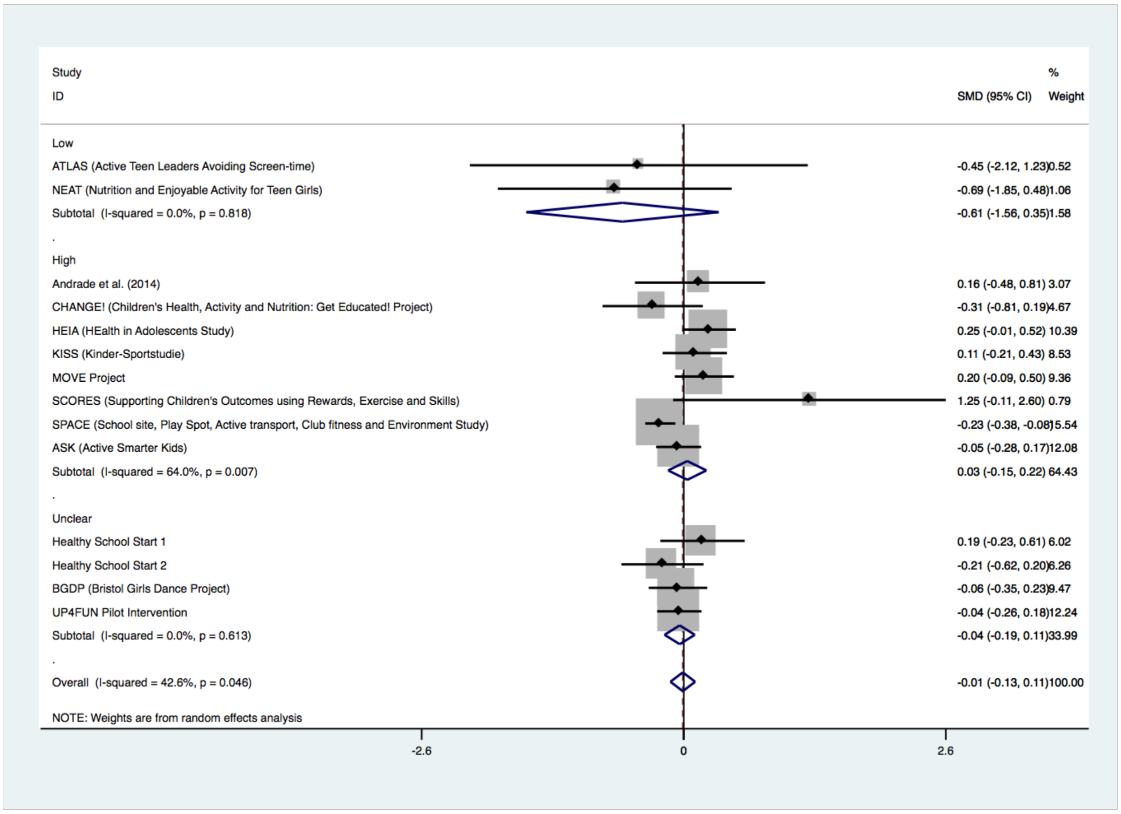

9.49 High SEP effect meta-regression by sample size (p-value: 0.029)**

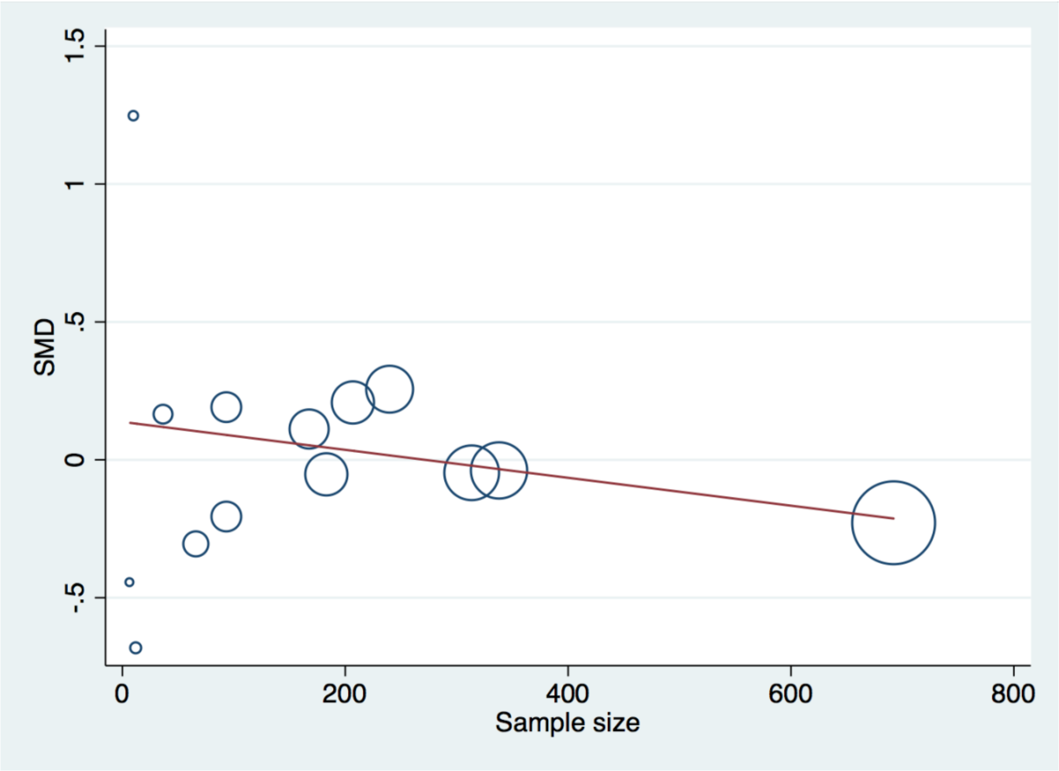

9.50 High SEP effect meta-regression by participant age (p-value: 0.542)

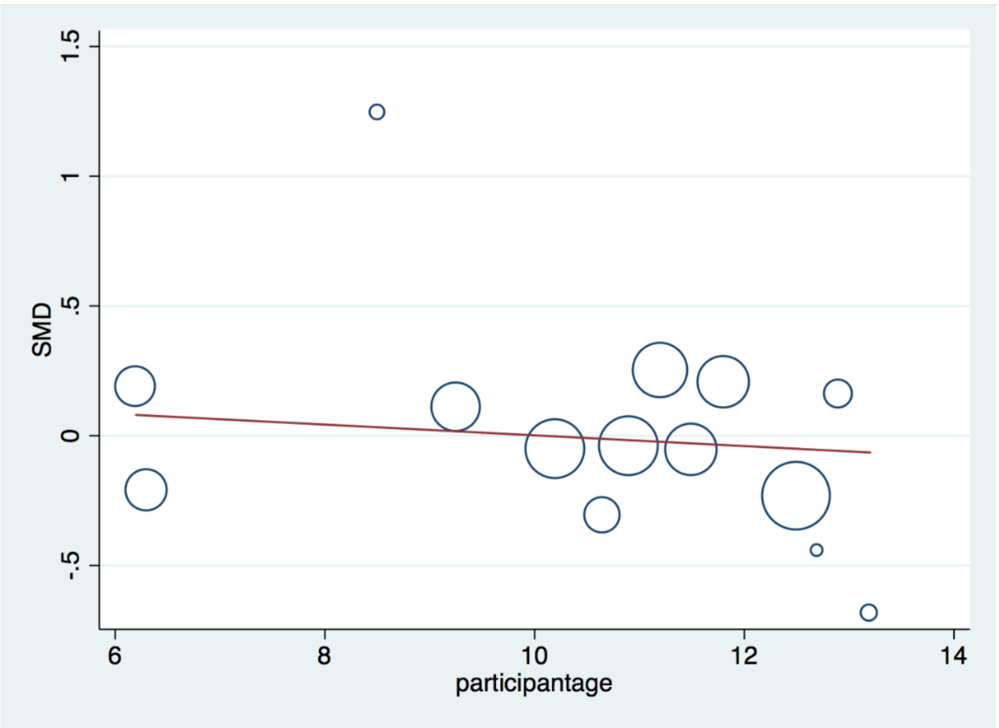

9.51 High SEP effect meta-regression by intervention duration (p-value: 0.082)

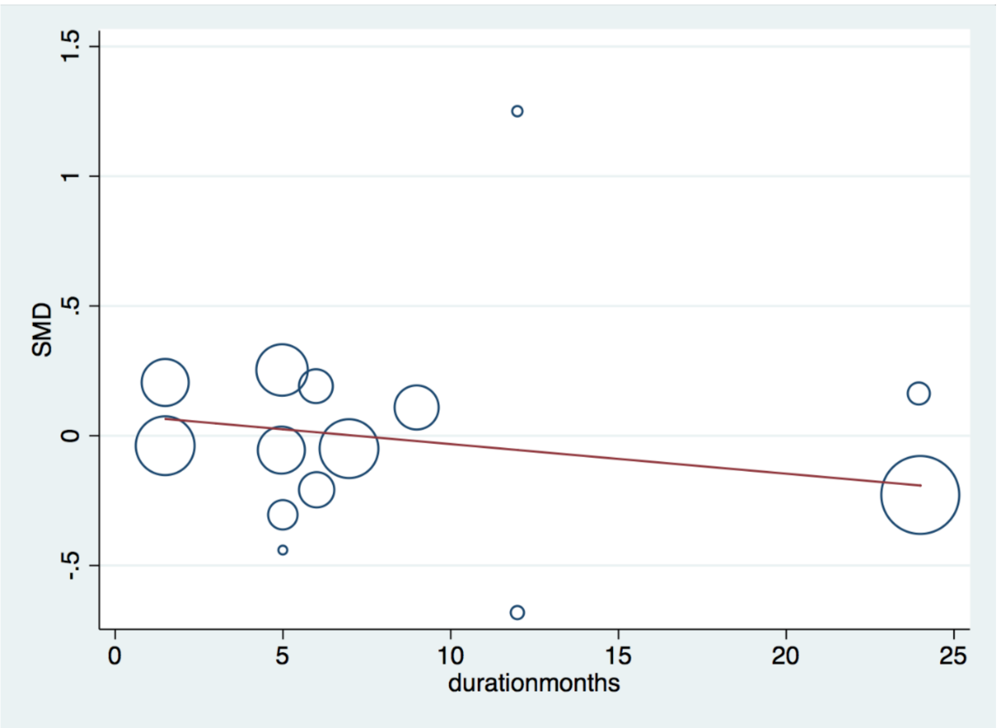

S10: Failsafe ratio of included trials

Trial	Failsafe Number
**Active by Choice Today (ACT)**	0.369393432
**Andrade et al. (2014)**	1.987772818
**ATLAS**	1.016884684
**CHANGE!**	6.007396298
**Drummy et al. 2016**	14.1020013
**Healthy School Start 1**	8.93322253
**Healthy School Start 2**	4.891004555
**HEIA Study**	-2.146305388
**KISS**	8.569520859
**MOVE Project**	18.66453466
**NEAT**	9.350694747
**Physical Activity 4 Everyone**	15.68801568
**SCORES**	19.14505705
**SPACE**	4.570687241
**The Active Smarter Kids Intervention**	11.43396927
**The Bristol Girls Dance Project**	23.05178785
**UP 4 FUN Pilot Intervention**	19.85089856

* Trials are added in the order to which they appear in the meta-analysis

S11: Risk of Bias assessment of included studies

	**Random sequence generation**	**Allocation concealment**	**Blinding of assessors at baseline**	**Incomplete outcome data**	**Selective Reporting**
Active by Choice Today (ACT)	Low risk of bias	Low risk of bias	Low risk of bias	Low risk of bias	Low risk of bias
Andrade (2014)	Low risk of bias	High risk of bias	Low risk of bias	Low risk of bias	Unclear risk of bias
ATLAS RCT	Low risk of bias	Low risk of bias	Low risk of bias	Low risk of bias	Unclear risk of bias
Change!	Low risk of bias	High risk of bias	High risk of bias	High risk of bias	High risk of bias
Drummy et al. (2016)	Unclear risk of bias	Unclear risk of bias	Unclear risk of bias	High risk of bias	Unclear risk of bias
Healthy School Start Study	Unclear risk of bias	Low risk of bias	Unclear risk of bias	Low risk of bias	Low risk of bias
Healthy School Start Study II	Low risk of bias	Low risk of bias	Unclear risk of bias	Low risk of bias	Low risk of bias
HEIA study	Low risk of bias	Low risk of bias	High risk of bias	High risk of bias	Low risk of bias
KISS	Low risk of bias	High risk of bias	Low risk of bias	Low risk of bias	Low risk of bias

MOVE Project	Low risk of bias	Low risk of bias	Unclear risk of bias	High risk of bias	Unclear risk of bias
NEAT girls	Low risk of bias	Low risk of bias	Low risk of bias	Low risk of bias	Low risk of bias
Physical Activity 4 Everyone	Low risk of bias	Low risk of bias	Low risk of bias	High risk of bias	Low risk of bias
SCORES	Low risk of bias	Low risk of bias	Low risk of bias	Low risk of bias	High risk of bias
SPACE Study	Unclear risk of bias	High risk of bias	Unclear risk of bias	Unclear risk of bias	High risk of bias
The Active Smarter Kids Intervention	Unclear risk of bias	Unclear risk of bias	Unclear risk of bias	High risk of bias	High risk of bias
The Bristol Girls Dance Project	Unclear risk of bias	Low risk of bias	Low risk of bias	Low risk of bias	Low risk of bias
UP4FUN pilot intervention (2012)	Low risk of bias	Unclear risk of bias	Unclear risk of bias	High risk of bias	Unclear risk of bias

** Note: Blinding of Outcome Assessment was removed for the included studies as we felt it was not applicable to the measurement of physical activity, objectively, through an accelerometer

S12: PRISMA Checklist

PRISMA 2009 Checklist

Section/topic	#	Checklist item	Reported on page #
**TITLE**			
Title	1	Identify the report as a systematic review, meta-analysis, or both.	1
**ABSTRACT**			
Structured summary	2	Provide a structured summary including, as applicable: background; objectives; data sources; study eligibility criteria, participants, and interventions; study appraisal and synthesis methods; results; limitations; conclusions and implications of key findings; systematic review registration number.	3
**INTRODUCTION**			
Rationale	3	Describe the rationale for the review in the context of what is already known.	4-5
Objectives	4	Provide an explicit statement of questions being addressed with reference to participants, interventions, comparisons, outcomes, and study design (PICOS).	5
**METHODS**			
Protocol and registration	5	Indicate if a review protocol exists, if and where it can be accessed (e.g., Web address), and, if available, provide registration information including registration number.	6
Eligibility criteria	6	Specify study characteristics (e.g., PICOS, length of follow-up) and report characteristics (e.g., years considered, language, publication status) used as criteria for eligibility, giving rationale.	6
Information sources	7	Describe all information sources (e.g., databases with dates of coverage, contact with study authors to identify additional studies) in the search and date last searched.	6
Search	8	Present full electronic search strategy for at least one database, including any limits used, such that it could be repeated.	S2
Study selection	9	State the process for selecting studies (i.e., screening, eligibility, included in systematic review, and, if applicable, included in the meta-analysis).	6 & 7
Data collection process	10	Describe method of data extraction from reports (e.g., piloted forms, independently, in duplicate) and any processes for obtaining and confirming data from investigators.	7
Data items	11	List and define all variables for which data were sought (e.g., PICOS, funding sources) and any assumptions and simplifications made.	7
Risk of bias in individual studies	12	Describe methods used for assessing risk of bias of individual studies (including specification of whether this was done at the study or outcome level), and how this information is to be used in any data synthesis.	7
Summary measures	13	State the principal summary measures (e.g., risk ratio, difference in means).	8
Synthesis of results	14	Describe the methods of handling data and combining results of studies, if done, including measures of consistency (e.g., I^2) for each meta-analysis.	8 & 9

Section/topic	#	Checklist item	Reported on page #
Risk of bias across studies	15	Specify any assessment of risk of bias that may affect the cumulative evidence (e.g., publication bias, selective reporting within studies).	7
Additional analyses	16	Describe methods of additional analyses (e.g., sensitivity or subgroup analyses, meta-regression), if done, indicating which were pre-specified.	8
**RESULTS**			
Study selection	17	Give numbers of studies screened, assessed for eligibility, and included in the review, with reasons for exclusions at each stage, ideally with a flow diagram.	Figure (F) 1
Study characteristics	18	For each study, present characteristics for which data were extracted (e.g., study size, PICOS, follow-up period) and provide the citations.	S8
Risk of bias within studies	19	Present data on risk of bias of each study and, if available, any outcome level assessment (see item 12).	S11
Results of individual studies	20	For all outcomes considered (benefits or harms), present, for each study: (a) simple summary data for each intervention group (b) effect estimates and confidence intervals, ideally with a forest plot.	F2-4
Synthesis of results	21	Present results of each meta-analysis done, including confidence intervals and measures of consistency.	F2-4
Risk of bias across studies	22	Present results of any assessment of risk of bias across studies (see Item 15).	S8
Additional analysis	23	Give results of additional analyses, if done (e.g., sensitivity or subgroup analyses, meta-regression [see Item 16]).	10
**DISCUSSION**			
Summary of evidence	24	Summarize the main findings including the strength of evidence for each main outcome; consider their relevance to key groups (e.g., healthcare providers, users, and policy makers).	11-14
Limitations	25	Discuss limitations at study and outcome level (e.g., risk of bias), and at review-level (e.g., incomplete retrieval of identified research, reporting bias).	11
Conclusions	26	Provide a general interpretation of the results in the context of other evidence, and implications for future research.	15
**FUNDING**			
Funding	27	Describe sources of funding for the systematic review and other support (e.g., supply of data); role of funders for the systematic review.	16

From: Moher D, Liberati A, Tetzlaff J, Altman DG, The PRISMA Group (2009). Preferred Reporting Items for Systematic Reviews and Meta-Analyses: The PRISMA Statement. PLoS Med 6(7): e1000097. doi:10.1371/journal.pmed1000097

For more information, visit: www.prisma-statement.org.
